# Transcription activation by the resistance protein AlbA as a tool to evaluate derivatives of the antibiotic albicidin†

**DOI:** 10.1039/d3sc00955f

**Published:** 2023-04-17

**Authors:** Simone Kosol, Lida Rostock, Jonas Barsig, Theresa Tabarelli, Kay Hommernick, Marcel Kulike, Tobias Eulberg, Maria Seidel, Iraj Behroz, Leonardo Kleebauer, Stefan Grätz, Andi Mainz, Roderich D. Süssmuth

**Affiliations:** a Institut für Chemie, Technische Universität Berlin Strasse des 17. Juni 124 10623 Berlin Germany roderich.suessmuth@tu-berlin.de

## Abstract

The rising numbers of fatal infections with resistant pathogens emphasizes the urgent need for new antibiotics. Ideally, new antibiotics should be able to evade or overcome existing resistance mechanisms. The peptide antibiotic albicidin is a highly potent antibacterial compound with a broad activity spectrum but also with several known resistance mechanisms. In order to assess the effectiveness of novel albicidin derivatives in the presence of the binding protein and transcription regulator AlbA, a resistance mechanism against albicidin identified in *Klebsiella oxytoca*, we designed a transcription reporter assay. In addition, by screening shorter albicidin fragments, as well as various DNA-binders and gyrase poisons, we were able to gain insights into the AlbA target spectrum. We analysed the effect of mutations in the binding domain of AlbA on albicidin sequestration and transcription activation, and found that the signal transduction mechanism is complex but can be evaded. Further demonstrating AlbA's high level of specificity, we find clues for the logical design of molecules capable of avoiding the resistance mechanism.

## Abstract 2

Exploiting the transcription regulatory activity of the AlbA resistance protein enables the design of improved albicidin analogs that can overcome the resistance mechanism.

## Graphical abstract

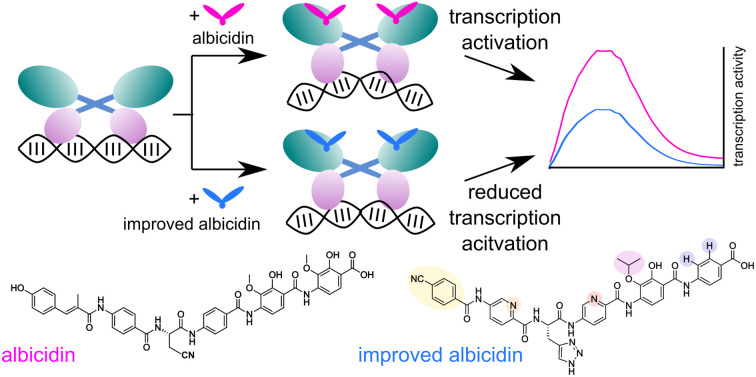

## Introduction

Antimicrobial resistance, one of the major threats to human health, presents a severe problem in the effective treatment of infections. The emergence of resistances against drugs used in the clinic, including many last resort antibiotics, incites a demand for new viable strategies to combat the looming antibiotic crisis.^1^ Combination therapy with antibiotic resistance breakers that re-sensitize resistant bacteria to antibiotics is one promising approach, as is the development of new antimicrobial analogs specifically designed to evade resistance mechanisms.^2,3^ But without doubt, new antibiotics and, ideally, new scaffolds are urgently needed.^4^ Nevertheless, to find and develop new effective antibiotics for therapeutic applications, knowing the molecular and mechanistic details of existing or emerging specific resistance mechanisms is fundamental. Equally important is the development of tools to evaluate the performance of promising drug candidates in the presence of resistance at early stages.

**Fig. 1:**
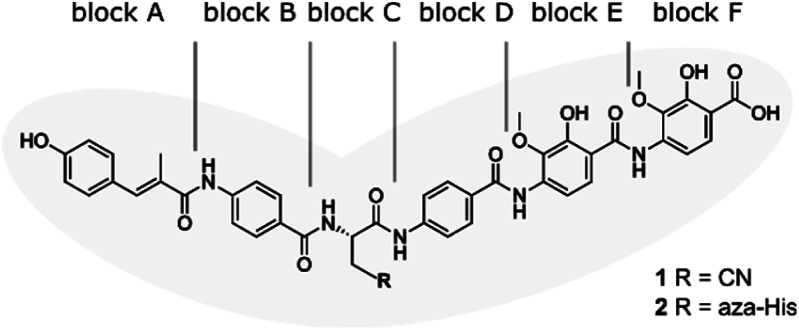
Structure of albicidin (1) and aza-His albicidin (2) with its six building blocks: methyl *p*-coumaric acid (MCA-1) in block A; a *p*-aminobenzoic acid (*p*ABA) in block B, β-cyano-l-alanine in block C (or aza-l-His in case of 2), a second *p*ABA in block D, and 4-amino-2-hydroxy-3-methoxybenzoic acids in blocks E and F. The grey frame in the background illustrates the shape of the molecule in structural studies.^9,40,41^

The peptide antibiotic albicidin, originally isolated from the plant pathogen *Xanthomonas albilineans*,^5,6^ is highly potent against a variety of Gram-negative and Gram-positive bacteria.^7^ Albicidin binds and stalls DNA gyrase (topoisomerase II) thereby blocking DNA synthesis, which leads to suppression of cell division and ultimately death.^8,9^ Notably, it has a relatively rigid structure due to its scaffold of unusual amino acids, which includes an N-terminal methyl *p*-coumaric acid, followed by two *para*-aminobenzoic acids (*p*ABA), a central l-cyanoalanine residue and two methoxybenzoic acids (*p*MBA) at the C-terminus^6^ (Fig. 1). Efforts by us and others to optimize and develop albicidins or the structurally closely related cystobactamids^10^ and coralmycins^11^ have resulted in derivatives with increased stability and potent antibacterial activity against several ESKAPE^12^ organisms, including *Acinetobacter baumannii*, *Enterococcus faecium*, *Staphylococcus aureus* and *Pseudomonas aeruginosa*.^13–19^*Klebsiella* species, on the other hand, have so far eluded most of our efforts and appear resistant against albicidin and most cystobactamids.^17^ A specific albicidin binding protein has been described in *K. oxytoca*,^20,21^ the MerR-family transcription regulator AlbA, which is at least partially responsible for the resistance. However, recent successes in creating analogues of cystobactamid with good antibacterial activity against *K. pneumoniae* by Wang *et al.*^18^ and Testolin *et al.*^19^ hold promise that it is possible to overcome this hurdle. The broad activity spectrum and its unique gyrase binding mode make albicidin an attractive lead for pharmaceutical development, despite several known (auto-)resistance mechanisms,^8,22–25^ which may be overcome by targeted engineering of the compound.^16,19,26^

**Fig. 2:**
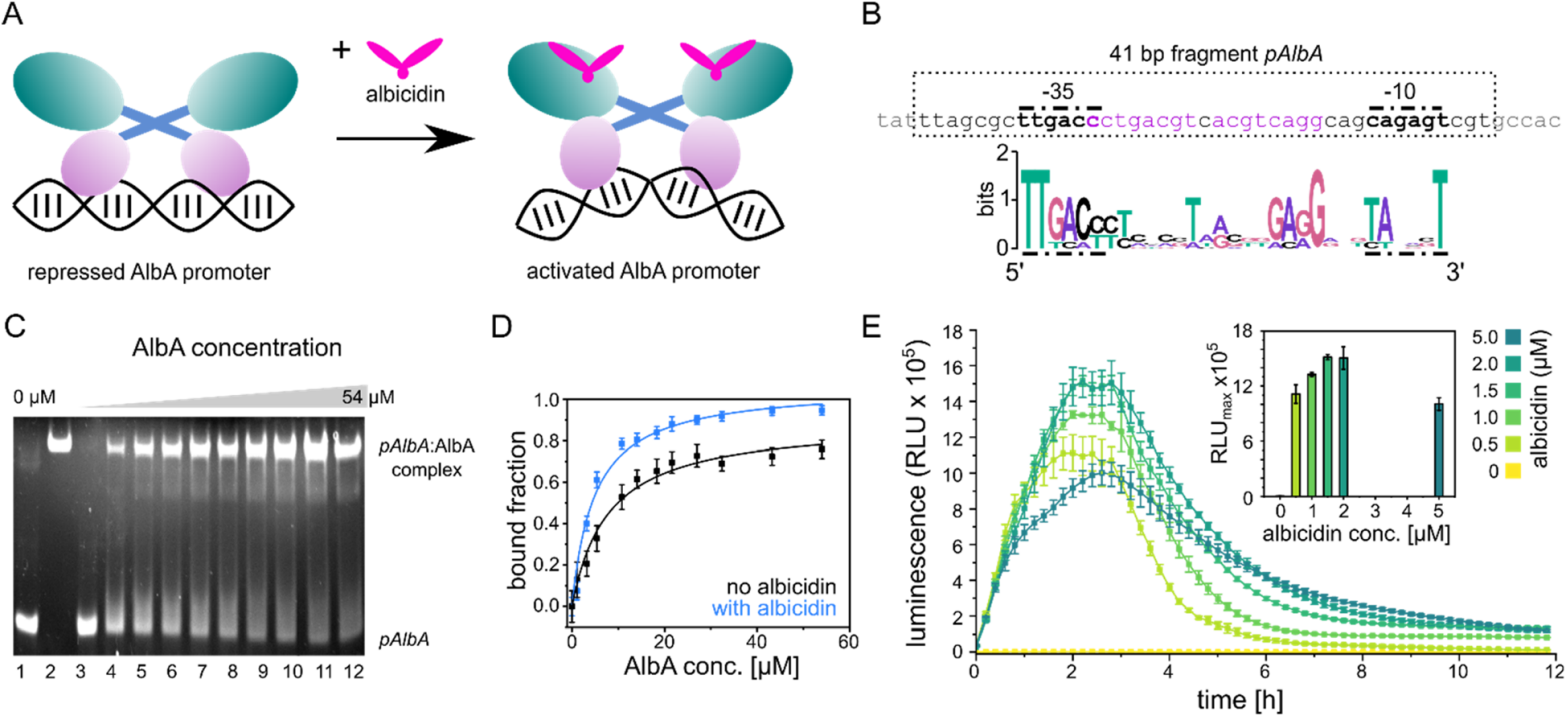
Promoter binding of AlbA. (A) MerR-type model of transcription activation by AlbA upon binding of albicidin. The LBD is coloured in teal, the coiled-coil domain in blue and the DBD is shown in purple. (B) Suggested palindromic binding site (two 8-base sequences separated by a 1-base spacer, highlighted in purple) of AlbA in the 19 bp long spacer between the −35 and −10 regions (bold nucleotides). The 41 bp fragment (*pAlbA*) containing the AlbA promoter region which was used in binding assays is indicated by a dashed box. The sequence logo below shows conserved bases in MerR-type promoters with 19 bp spacers.^30^ (C) Ethidium bromide-stained EMSA gel of the DNA fragment *pAlbA* (100 ng per lane) incubated with increasing concentrations of AlbA (lanes 3-12). Controls of *pAlbA* with 10.2 μM AlbAS (AlbA without DBD) (lane 1) and AlbA alone (lane 2, *c* = 18.4 μM) are shown on the left side of the gel. (D) Gel band intensities in the EMSA assays fitted against AlbA concentrations show binding of AlbA to *pAlbA* in absence (black) and presence (blue) of 1.5× fold excess of albicidin (gels see Fig. S1A†). The fitting resulted in dissociation constants *K*_d_ of 7.8 ± 1.1 μM and 4.9 ± 0.7 μM, respectively. Error bars represent gel band quantification errors based on noise levels. (E) Time curve of luminescence intensities during the response reporter assay after addition of 0, 0.5, 1.0, 1.5, 2.0 and 5.0 μM aza-His albicidin (2). The error bars represent the standard deviation of each data point measured in triplicate. The inset shows the maxima of each curve with the error bars representing the standard deviation of each maximum.

The resistance factor AlbA is a member of the MerR-like transcription regulator family, which typically reshape promoter DNA to induce transcription of downstream genes.^27^ Many MerR-family transcription factors act as sensors of unfavourable conditions, such as metal- or oxidative stress-response sensors and multi-drug responsive regulators, by employing a ligand-binding domain (LBD). Ligand binding, an imbalance of metal ions or oxidative stress trigger transcription activation by the MerR-proteins, which act as dimeric repressors in absence of a trigger.^28,29^ Typically, MerR promoters exceed the optimal 17 ± 1 bp spacer length between the −35 and −10 elements by two or three base pairs.^30^ When activated, MerR family regulators undergo allosteric changes that introduce kinks in the backbone of the bound DNA (Fig. 2A), which positions the −10 element so that the transcription bubble can be formed.^28,31–34^ Excellent studies with detailed structural insights into this process have recently been published for related MerR multi-drug regulators EcmrR^35^ or BmrR^31,36^ as well as for the metal stress-response factor CueR,^37,38^ which illustrate the DNA binding mode and transcription activation by MerR-family members.

Within the MerR-family, a large variety of LBDs (also called C-terminal effector domains) have been described.^34^ Of the known MerR-family members, AlbA has the highest similarity to TipA,^39^ which binds and is activated by thiostrepton.^40,41^ AlbA acts as a self-regulatory sponge, capturing albicidin with high affinity^21^ and is expressed as two translation products in cells,^20,42^ similarly to TipA, where the LBD can function independently.^41^ The longer translation product (also referred to as AlbAL) is a di-domain protein consisting of the MerR-typical N-terminal DNA-binding domain (DBD) with a helix-turn-helix motif and a coiled-coil dimerization region that is connected to an LBD. In AlbA, this C-terminal domain is twice as large compared to TipA and appears to have arisen from a gene duplication event of a domain orthologous to the thiostrepton binding domain.^39,42^ Recently, our group^39^ and the Müller lab^42^ proposed an albicidin binding mechanism that involves a subdomain motion within the LBD where both halves of its pseudo-dimer, the N-terminal domain (NTD) and C-terminal domain (CTD), act in concert to trap albicidin. Sikandar and colleagues postulated an additional function of the ligand-binding domain in reducing the activity of albicidin by promoting cyclization of the cyanoalanine side-chain.^42^

AlbA or (structural) homologs such as AlbB from *Alcaligenes denitrificans*^43^ are fairly abundant among Gram-negative bacteria, including ESKAPE pathogens such as *Pseudomonas aeruginosa*, *Enterobacter* sp. or *Acinetobacter baumanii*.^39,42^ AlbA-based resistance against albicidins and cystobactamids can therefore be anticipated and must be considered in the development of the drug scaffold for clinical use. In principle, overcoming the AlbA resistance mechanism can be achieved in two ways: either by impeding albicidin binding to the LBD or by stalling the allosteric changes that lead to transcription activation by AlbA.

Here, we identified the palindromic promoter binding region of AlbA, established a bioluminescence-based transcription reporter assay and assessed a number of novel albicidin derivatives. In addition, we screened truncated albicidin derivatives, different DNA-binders and gyrase poisons to gain insights into the target spectrum of AlbA. Moreover, to better understand how albicidin binding induces the structural changes that are required for transcription activation, we introduced several mutations in the effector binding domain, which considerably affected gene activation. We show that AlbA is quite specific and obtain hints for the rational design of more effective albicidin compounds that can evade the resistance mechanism.

## Results and discussion

### AlbA binds a palindromic promoter

The promoter sequences that are recognized by MerR-like proteins have an unusually long 19- or 20-bp spacer region (instead of the common 17 bp) between the −10 and −35 elements. The regulator binding site is typically a dyad symmetrical sequence of a 2× ∼10-bp palindrome that may be separated by a short spacer.^30^ After inspecting the upstream region of the *albA* gene in the genome of *K. oxytoca* NCTC13775 (NCBI reference NZ_UGJO01000002.1), we identified a region that fulfils these criteria and aligns with other 19-bp-long MerR-like promoters (Fig. 2B). The palindrome of the *pAlbA* operator consists of two 8-base sequences separated by a 1-base spacer where the first two bases overlap with the −35 element (Fig. 2B). The sequence was not found anywhere else in the genome of *K. oxytoca*.

To confirm that we identified the correct operator site, we annealed two complementary oligonucleotides to obtain a 41 bp DNA fragment that contained the −10 and −35 elements as well as the putative reporter binding sequence (Fig. 2B). Using electrophoretic mobility shift assays (EMSA), we verified that AlbA indeed binds the 41 bp DNA fragment (Fig. 2C), but not a DNA sequence of 41 bp randomly chosen from the genome of *K. oxytoca* (Fig. S1A and C†). In a second control reaction, no binding to *pAlbA* was observed for the shorter translation product AlbAS, which lacks the DNA binding domain (Fig. 2C). Unliganded AlbA bound to the promoter region, but stronger binding was observed in the presence of albicidin: the dissociation constant *K*_d_ improved from 7.8 ± 1.1 μM to 4.9 ± 0.7 μM when a 1.5-fold excess of albicidin was present (Fig. 2D and Fig. S1A†). This is consistent with other reports where an increase in affinities of MerR-family proteins for their cognate operator upon ligand binding has been described, for example the transcription regulators MtN,^32^ BmrR^32^ or TipA^44^ with promoter binding affinities in the low μM to nM range. However, in the case of MerR^27^ or SoxR,^45^ the binding affinity for their cognate operator sequences does not change in the presence of ligands or is lower than in the unliganded form.

### Transcription is activated at the operator *pAlbA* in the presence of albicidin

Having established the operator sequence that is recognized by AlbA, we introduced the 41-bp-long fragment (Fig. 2B) into a reporter vector that encodes the *ilux*^46^ luminescence cassette (pCS-pAlbA-ilux; Fig. S2A and B†). To create a system similar to the *albA* operon in *K. oxytoca*, the distance between the −10 region and the ribosome binding site was kept intact (16 bp). The *ilux* cassette includes the optimized bacterial luciferase genes *lux*CDABE from *Photorhabdus luminescens* as well as an additional FMN reductase to recycle FMNH_2_ faster.^46^ FMNH_2_ is oxidized by the luciferase together with an aliphatic aldehyde that is oxidized to a carboxylic acid, which results in the emission of blue-green light (*λ*_max_ = ∼490 nm). In our reporter system, the addition of albicidin to *E. coli* cells that contain the reporter plasmid and express *albA* should activate AlbA to bend the DNA so that transcription is initiated at the *pAlbA* promoter, leading to production of the ilux proteins and thus light emission.

To confirm activation of the reporter gene expression by albicidin, we transformed *E. coli* BL21(DE3) cells with the reporter plasmid and a pET15b vector encoding AlbA without any purification tags (pET15b-ΔHisAlbA, Table S4†). We monitored luminescence over a period of 12 hours after addition of albicidin (1) or aza-His albicidin (2) and observed light emission only, if 1 or 2 was added and both plasmids were present (Fig. 2E, S3A and B†). The signal started to increase within the first 30 min and the maximum was reached approximately 2 hours after exposure to albicidin. This agrees with the findings of Sikandar and colleagues, who reported upregulation of the transcription of the *albA* gene in *K. pneumoniae* between 90 and 240 min after addition of albicidin.^42^ Because of the better stability and solubility of 2, which has an aza-His residue in place of the cyanoalanine building block (Fig. 1), we decided to use 2 as a standard in our assays, which has comparable activity and binds AlbA with similar affinity.^39^

We then compared the light emission after addition of 0, 0.5, 1.0, 1.5, 2.0 and 5.0 μM of 2 and observed a higher luminescence signal at increasing effector concentrations (Fig. 2E, S3C and D†). However, the luminescence output was reduced again at concentrations of 5 μM aza-His albicidin, very likely due to its antibacterial activity. This is also represented in measured OD values, which virtually did not increase during cultivation in the presence of 2 (Fig. S3E†). In *Klebsiella*, exposure to albicidin would induce production of AlbA and its shorter variant, AlbAS, allowing the cells to trap the toxin. But since *E. coli* is susceptible to albicidin and transcription activation leads to expression of the luminescence cassette genes instead of *albA*, the cells only have limited protection from albicidin through basal *albA* expression from pET15b, without induction by isopropyl β-d-1-thiogalactopyranoside (IPTG). At all measured aza-His albicidin concentrations, light emission dropped to baseline levels after six to eight hours.

### The N-terminus of albicidin is important for AlbA transcription activation

**Fig. 3:**
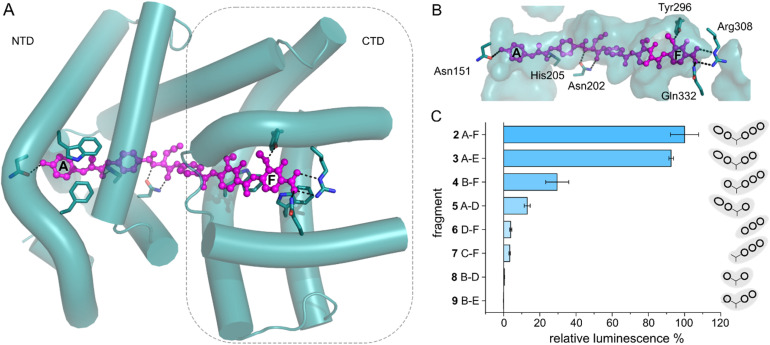
AlbA transcription activation by shorter albicidin fragments. (A) Crystal structure of the ligand binding domain of AlbA (AlbAS, cyan, PDB-ID: 6et8) with H-bonds to albicidin (magenta) shown as dashed blue lines. Side-chains of amino acid residues involved in H-bonds or π–π interactions are shown as sticks with nitrogen atoms in blue and oxygen atoms in red. (B) Surface representation of the binding pocket with bound albicidin. AlbA residues involved in hydrogen bonds with albicidin are shown as sticks. Amino acid labels correspond to their position in full-length AlbA. (C) The transcription response of AlbA upon addition of 1.5 μM of an albicidin fragment (3–9) is normalized to the full-length control (2). Fragments B-D (8), A-D (5) and 2 contain the aza-His in block C (Fig. 1 and S5†). Error bars depict the standard deviation of the mean maximum of triplicate measurements.

With the transcription reporter assay established, we set out to investigate details of the activation mechanism. Based on NMR and circular dichroism (CD) data, it had been suggested that the LBD undergoes structural changes upon albicidin binding, resulting in secondary structure with more α-helical elements compared to unliganded LBD.^39,47^ Contrary to that, the crystal structure of AlbAS without albicidin was almost identical to the liganded protein, presumably because only the closed conformation crystallized (0.8 Å RMSD between both structures).^42^ In the current model, albicidin connects the pseudodimeric C- and N-terminal halves of the LBD and stabilizes their fold^39^ (Fig. 3A, B and S4A–D†). AlbA interacts with albicidin *via* polar and hydrophobic interactions such as hydrogen bonds to blocks A, C and F as well as π–π interactions with blocks A, E and F (Fig. 3A and B). In the crystal structure, building blocks A, B, and C of albicidin are surrounded by the NTD of AlbAS, while building blocks D, E, and F are enveloped by the CTD.^39,42^ Shorter albicidin derivatives were found to be not sufficient to stabilize the NTD-CTD arrangement and bound AlbAS with a much lower affinity compared to albicidin.^39^

To investigate how binding of truncated albicidin derivatives affects transcription activation and to identify how many and which of the building blocks of albicidin are required for transcription induction, we conducted transcription activation assays with a panel of shorter albicidins (compounds 3–9 and Fig. S5†). While the absence of building block F was easily tolerated with only a small decrease in the luminescence signal, removal of building block A had a considerably more pronounced effect resulting in significantly decreased transcription activation (Fig. 3C). Little transcription activity was observed for fragments with four or fewer building blocks, particularly those lacking building block A. Despite forming a salt bridge and two H-bonds with AlbA (Fig. 3A, B and S4D†), the F building block appears less important for the induction of transcription compared to building block A, which has a hydroxy group that forms a hydrogen bond to Asn151 (Fig. 3A, B and S4D†). All fragments have a C-terminal carboxylic acid and therefore it cannot be ruled out that, in compound 3, the negative charge at block E might partially compensate for that of the missing block F. It is interesting to note that the smaller TipAS, which is engaged in thiostrepton-binding, contains the same portion of the binding pocket that block A occupies. However, fragment 5, consisting only of blocks A-D, showed only around 10% transcription activation compared to 2 (Fig. 3C). These findings support the hypothesis that AlbA transcription activation requires binding and stabilization of both LBD subdomains.

### Variations in the ligand binding site affect albicidin binding and transcription activation

The two sub-domains of the ligand binding domain interact with albicidin through six H-bonds, a salt bridge and three π–π interactions that involve almost every building block^39,42^ (Fig. 3A and B). To further investigate which contacts are important for transcription activation, we constructed several mutants of AlbA. We selected amino acids in the CTD and NTD that form contacts to albicidin (Asn202, Trp260, Trp289, Tyr296, Arg308, Gln332), and residues in the NTD that might play a role in the opening/closing of the subdomains (P219) and signal transduction to the DNA-binding domain (P209) (Fig. S4E, F and S6A†). In addition, we included a mutant of H252 which had been found to affect albicidin binding despite not being part of the binding pocket.^39,48^ Several studies in the past have used mutagenesis to analyse the role of selected residues in albicidin binding in the shorter AlbAS protein^39,42,47,48^ (Table S1†). No single mutation led to complete loss of albicidin binding, and it was suggested that several residues work in concert to trap albicidin, which was confirmed by the crystal structures of AlbA.^39,42^ However, so far, it has not been investigated how mutations in the LBD might affect transcription activity. To close this gap, we used growth inhibition assays, transcription activation assays, and Trp fluorescence quenching to determine the effects of 11 mutations (N202A, P209A/G, P219A/G, H252A, W260A, W289A, Y296A, R308A, and Q332A) on the interaction with albicidin.

**Fig. 4:**
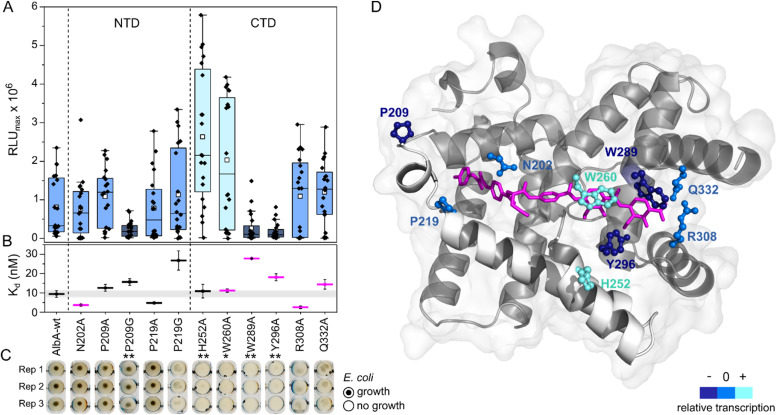
Transcription activation of AlbA variants is not coupled to tight albicidin binding. (A) Box plots and superimposed data points (black diamonds) of the luminescence signal maxima of AlbA mutants and wild-type AlbA (*n* = 20). The boxes show the interquartile range between upper and lower quartiles, the median (black line) and mean (white squares) are shown. Error bars show 1.5× interquartile range. Mutants with significantly different luminescence output are marked with one (*, Wilcoxon–Mann–Whitney test *p* ≤ 0.05) or two (**, *p* ≤ 0.01) asterisks. (B) Dissociation constants (*K*_d_) of AlbA variants and 2 (see Table S2†). Mutants with variations in the binding pocket are highlighted in magenta. The grey bar shows the fitting error interval of the wild-type protein *K*_d_ and error bars show the fitting errors. (C) *E. coli* growth inhibition assays in the presence of AlbA or a mutant protein and 2. Triplicates for each mutant are shown (Rep 1–3). (D) Structure of AlbAS (transparent grey, PDB-ID: 6et8) with bound albicidin in pink and the clamp helices shown in opaque white. Residues altered in the mutant proteins are depicted as sticks and balls coloured according to their performance in transcription activation assays compared to AlbA-WT.

For the transcription activation assays, we transformed *E. coli* BL21(DE3) cells with the pCS-pAlbA-ilux reporter vector and pET15b-ΔHisAlbA plasmids containing the mutations. Due to variations in luminescence output of the same transformant (even under identical cultivation conditions), smaller differences in transcription level cannot be attributed to the mutations. Nonetheless, we collected transcription activation data sets of all mutants as well as the wild-type and a significant difference between the performance of the mutants was apparent after 2 was added (Fig. 4A; *n* = 20; Kruskal–Wallis, *p* < 0.001). To evaluate the mutants for their ability to bind albicidin and protect the bacteria from the antibiotic, *E. coli* DSM1116 cultures were incubated over-night in 96-well plates with equimolar amounts (10 μM) of 2 and purified AlbA or mutant protein (Fig. S6B and C†). In this qualitative growth inhibition assay, cell pellet is only visible if the protein binds 2 sufficiently strong to protect the growing cells. The expression levels and secondary structure content of the mutants were comparable to that of AlbA-WT (Fig. S7A–C†). Of the eleven mutants tested, P209G, W289A and Y296A had significantly lower transcription activity compared to the wild-type protein (Wilcoxon–Mann–Whitney test, *p* ≤ 0.01, *z*-values of 2.62, 3.16 and 3.49, respectively, *versus* a critical value of *z* = 2.576). In case of P209G, 2 was still sufficiently trapped to protect *E. coli* in growth inhibition assays but the other two mutations led to strongly decreased protection (Fig. 4C). While all three mutants had dissociation constants in the low nanomolar range, W289A and Y296A bound albicidin with three- and two-fold decreased affinity, respectively, compared to AlbA-WT (*K*_d_ = 9.4 ± 1.7 nM; Table S2, Fig. 4B and S8†). Curiously, the mutants H252A and W260A, whose ability to protect cultures from albicidin was strongly diminished, had unusually high transcription regulation response (Wilcoxon–Mann–Whitney test, *p* ≤ 0.05, *z*-values of −3.57 and −1.97, respectively, *versus* a critical value of *z* = 1.96; Fig. 3A and B) and dissociation constants very similar to that of AlbA-WT (*K*_d_ = 10.9 ± 3.5 nM for H252A and *K*_d_ = 11.2 ± 0.9 nM for W260A; Table S2, Fig. 4B and S8†). Only three mutants (N202A, P209A, P219A) performed similarly to wild-type AlbA in growth inhibition assays as well as transcription activation assays. Mutations affecting the residues R308A and Q332A, which form H-bonds to albicidin's F-block (Fig. 3A, B and 4D), did not change the luminescence output but disturbed the binding mechanism so the cells were not fully protected despite maintaining low affinity constants. These results agree well with reported data that residues H252, W289, R308 and Q332 play a role in trapping albicidin^39,47,48^ (Table S1†). This suggests that interactions with the EF-region of albicidin are important for its capture, which is further supported by the observation that the mutants Y296A, which forms an H-bond with the methoxy group on building block F and W260A involved in π–π interactions with block E, also failed to trap albicidin in growth inhibition assays (Fig. 3A, B, 4C and D).

However, the ability to trap albicidin does not appear to correlate with the transcription activation potential or binding affinity. On the contrary, the two mutants that have shown the strongest transcription activation, H252A and W260A, did not trap 2 sufficiently to protect cells but had similar affinities for 2 as the wild-type. In case of H252A, this might be because the residue is not located in the binding pocket and does not directly interact with albicidin^39^ (Fig. 4D). H252 sits at the end of helix I′, the first helix of the C-terminal half of the pseudodimer, which together with helices V and VI connects the two subdomains like a clamp (Fig. 4D and S6A†). The imidazole side-chain can potentially form critical contacts to stabilize either the open or closed conformation and relay the allosteric changes required for transcription activation. This is also consistent with the pH-dependence of albicidin binding and the associated conformational changes.^21,47^ That the three-helix clamp plays a role in stabilizing the transcription activating conformation of AlbA is further supported by the strongly diminished transcription response when P209, which is located on helix V at the other end of the clamp (Fig. 4D and S6A†), was replaced with a glycine. Generally, the Trp fluorescence quenching measurements resulted in *K*_d_ values in the low nanomolar range which is in good agreement with the strong binding affinities reported before.^39,42^ Mutant proteins that were able to trap albicidin efficiently in growth inhibition assays (N202A, P209A/G, P219A) had *K*_d_ values similar to the wild type (Fig. 4B). Surprisingly, the replacement of N202 with alanine to remove the H-bonds with the backbone amide and carbonyl of the C block did not affect transcription activity or albicidin sequestration and binding affinity was even improved. Rostock *et al.* had observed reduced albicidin neutralization by the triple mutant AlbAS-N202A/R308A/Q332A,^39^ which in light of these results was likely caused by the loss of the arginine and glutamine residues. The data also supports the hypothesis that the mechanism of albicidin capture is more complex and that the conformational dynamics of AlbA play an important role. More information about the binding kinetics is required to fully understand the effect of each mutation on *k*_on_ and *k*_off_ rates and to gain further insights into the mechanism. However, our data also showed that signal transduction is possible when albicidin is not fully trapped (*k*_signal_ ≫ *k*_off_) but also that albicidin can be trapped without activating transcription (*k*_signal_ ≪ *k*_off_). This promises that even if some albicidin derivatives might not be able to evade capture, they might overcome AlbA resistance by uncoupling transcriptional control.

### Screening of potential AlbA effector molecules

**Fig. 5:**
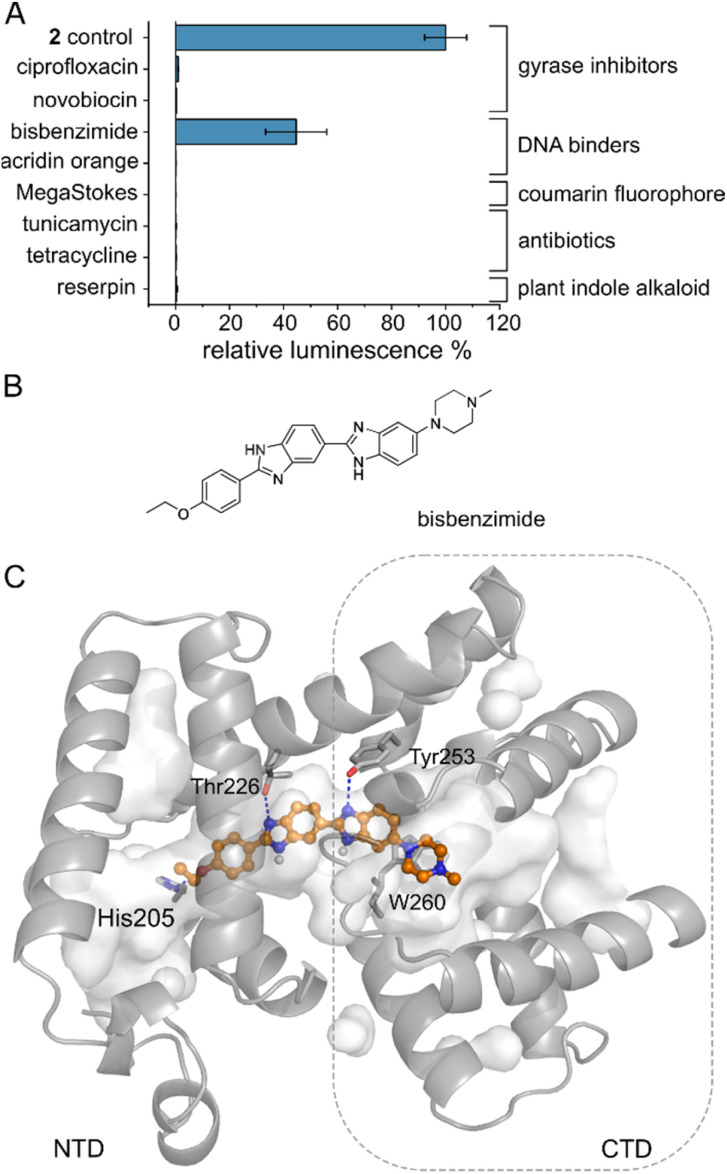
The AlbA binding pocket is specific. (A) Relative luminescence in transcription activation assays with DNA gyrase inhibitors as well as DNA binders. Error bars represent the standard deviation of the mean maximum from triplicate measurements. See Fig. S9† for the chemical structures. (B) Structural formula of bisbenzimide (Hoechst33342) and (C) AutoDock Vina^50^ model of bisbenzimide docked in the AlbA binding pocket. Sidechains of residues interacting with bisbenzimide *via* H-bonds (dashed blue lines) or π–π interactions are shown as sticks. Oxygen atoms are indicated in red, nitrogen is depicted in blue and polar hydrogens are shown as white balls.

Several MerR-family members are multidrug sensors but AlbA has so far only been associated with albicidin binding and resistance.^42,47^ To test if other biologically active molecules with aromatic building blocks would bind AlbA and induce transcription, we selected the gyrase inhibitors ciprofloxacin and novobiocin, the DNA-binding dyes bisbenzimide (Hoechst 33342) and acridin orange, a coumarin fluorophore (Azid MegaStokes), the antibiotics tunicamycin and tetracycline and the plant alkaloid reserpine (Fig. S9†). We added the compounds to transcription activation assays and normalized the luminescence output to that of 2 (Fig. 5A). Of the eight tested molecules, only the minor groove-binder bisbenzimide^49^ elicited a transcription response (Fig. 5A and S3F†). However, the luminescence output was lower compared to 2 although a higher concentration (5 μM) of bisbenzimide was used. Although shorter than albicidin, docking models showed that bisbenzimide may occupy the binding pocket of AlbA, forming polar and hydrophobic contacts with residues located in the NTD and the CTD. The two benzimidazoles may form H-bonds with Thr226 and Tyr253 as well as a π–π interaction with Trp260 (Fig. 5C, S10†). Additionally, His205 could potentially form an H-bond with the ether group which replaces the carbonyl of albicidin's building block A as H-bond acceptor (Fig. 3A, B and S4D†). Residue Tyr253, which, despite not forming a direct contact, plays an important role in trapping albicidin^47^ and is located adjacent to the above discussed His252. As a result, the bisbenzimide molecule may bridge the NTD and CTD similarly to albicidin and fulfil the necessary interactions to achieve signal transduction. It appears that the substrate scope of AlbA is restricted by complex structural requirements. Even so, the observed transcription activation by bisbenzimide shows that the ligand does not have to be peptidic or acidic in nature. With the shape of bisbenzimide resembling that of albicidin, it seems likely that aromatic building blocks, a certain length and planarity are prerequisites for recognition by AlbA.

### Albicidin derivatives can escape AlbA

We set out to test a set of albicidin analogues with a variety of modifications (10–22 and Fig. S11†) to further investigate AlbA ligand specificity and find leads for compounds that can evade the resistance mechanism but retain their antibacterial activity. The derivatives contained variations of the A block in place of the cinnamoyl moiety (10, 11, 13), a different C block (17, 18, 20, 22), amide bond isosteres of the peptide bond between blocks D and E (12, 19, 21), or modifications of the methoxy groups on blocks E and F (11, 13–16). The activity of each compound was tested in minimal inhibitory concentration (MIC) and DNA gyrase inhibition assays. To evaluate the performance of the derivatives in the presence of AlbA, we again combined *E. coli* growth inhibition assays with transcription activation assays.

**Fig. 6:**
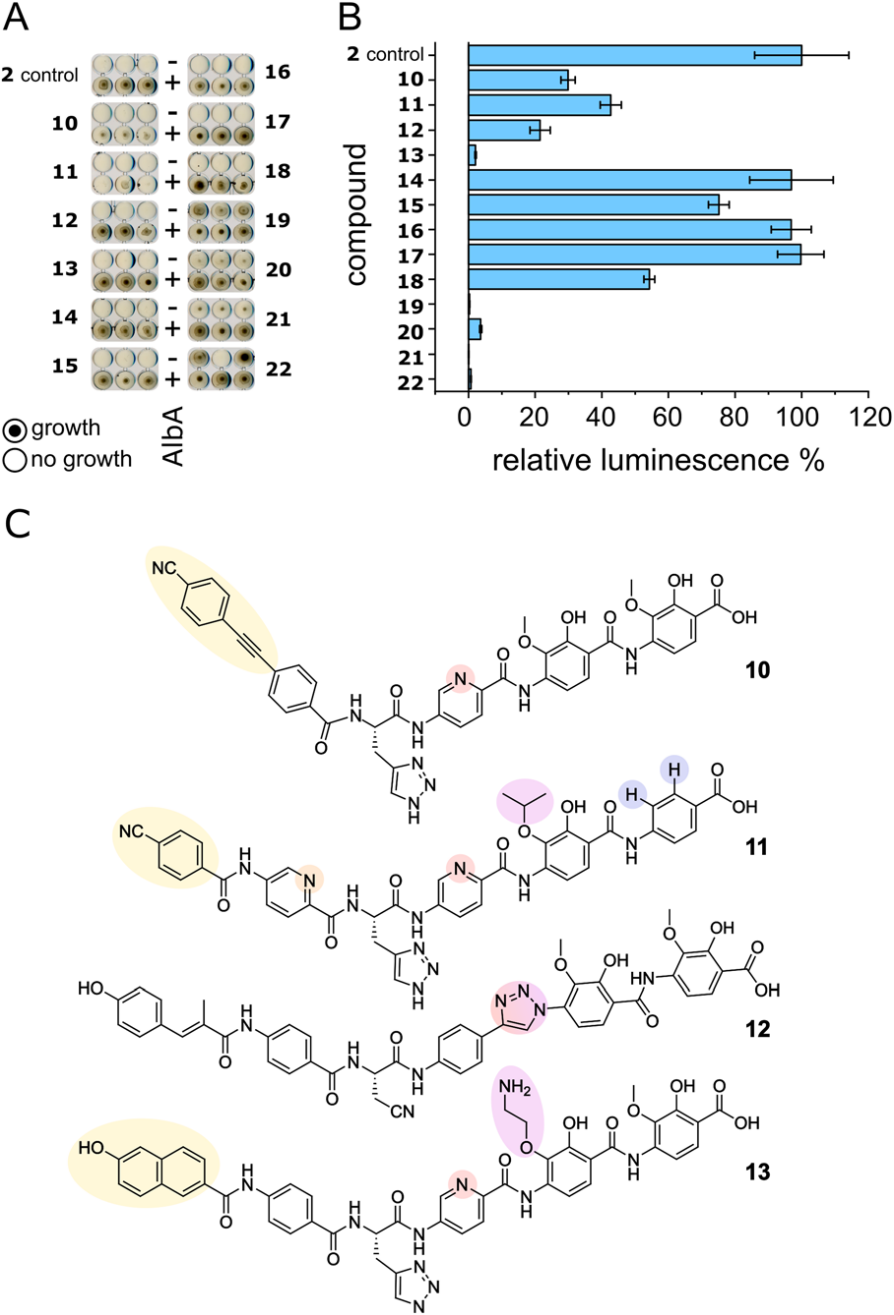
AlbA binding and transcription response of albicidin derivatives. (A) *E. coli* growth inhibition assays employing albicidin derivatives 10–22 (Fig. S11†) preincubated with AlbA. In the upper rows (−), only the derivative was added to monitor the antibiotic activity and in the bottom rows (+), an equimolar mixture of the derivative and AlbA was added. Triplicates for each derivative are shown. (B) Relative luminescence in transcription activation assays with albicidin derivatives 10–22. Error bars represent the standard deviation of the mean maximum from triplicate measurements. (C) Chemical structures of albicidin derivatives 10–13 with low transcription activation and antibacterial activity. Variations of building blocks are highlighted.

Of the 13 tested derivatives, only analogues 10 and 11 evaded capture by AlbA sufficiently to inhibit *E. coli* growth, albeit not completely (Fig. 6A and S12†). Encouragingly, the two analogues also performed very well in MIC assays (Table S3†) and showed significantly reduced transcription activation (Fig. 6B). Several other derivatives (12, 13, 19–22) showed strongly diminished transcription activation but did not perform as well in growth inhibition assays and had lower antibacterial activity compared to albicidin. It is likely that in case of the l-hydroxyproline-containing compound 22, the *N*-methylated analogue 19 and the sulfonamide-containing 21, the geometry and planarity of the molecule change drastically, interfering with gyrase inhibition (Table S3†) and perhaps also cellular uptake by the nucleoside transporter Tsx.^23^ The triazole-containing analogue 12, compound 13 with a naphthol in block A and an aminoethoxy modification on block E as well as guanidino-albicidin 20 showed little transcription activation but unfortunately also low antibacterial activity (Fig. 6A and B). That the two best performing derivatives contain modifications of the A block further corroborates our hypothesis that the N-terminus is important for inducing transcription and suggests that A block modifications could lead to analogues that can evade AlbA resistance. The A block has also been shown to be critical for DNA binding during gyrase poisoning,^9^ making it an ideal target for engineering. However, all analogues, with the exception of 22, still bound AlbA, albeit most bind with lower affinity (Fig. S13†). Analogue 11 was perhaps performing particularly well because it contains an isopropoxy group on block E, similar to cystobactamid which is isopropoxylated on blocks E and F. Cystobactamid has been reported to bind AlbAS but is not sufficiently trapped in agar diffusion assays to protect *E. coli* and it fails to induce transcription of the AlbA gene in *K. pneumoniae*.^42^ Since no such benefit was observed for analogue 16 which performed similarly to 2 in all assays, a combination of isopropoxylation and A block variation might be promising adjustments for future albicidins. Indeed, a recently described cystobactamid analogue which showed good activity against *K. pneumoniae* contained an A block modification (l-Phe instead of the *p*-nitro-benzoic acid).^18^

## Conclusions

We developed a tool to evaluate the performance of albicidin derivatives in the presence of the resistance factor AlbA and showed that the transcription activation of AlbA is not directly coupled to high affinity binding or the ability to protect cells from albicidin. This might be due to induced folding of the ligand binding domain upon albicidin binding as observed in NMR spectroscopic studies.^39^ However, to fully understand the allosteric coupling between albicidin binding and activation of transcription further studies are required. We demonstrated that it is possible to create highly potent albicidins that can evade both aspects of the AlbA resistance mechanism. Together with SAR studies, our assay allows us to screen new analogues directly against the resistance mechanism and inform the design of novel derivatives.

## Experimental

Experimental descriptions can be found in the ESI.†

## Data availability

The datasets supporting this article have been uploaded as part of the ESI.†

## Author contributions

SK, AM and RDS designed the study, analysed the data and wrote the manuscript. SK, LR and AM designed and conducted experiments. MS conducted and analysed antibiotic activity assays. TT, JB and TE carried out molecular biological and biophysical experiments. KH, MK, IB, LK and SG designed and conducted the chemical synthesis of albicidin and its derivatives. All authors contributed to the final manuscript.

## Conflicts of interest

There are no conflicts to declare.

## Supplementary Material

SC-014-D3SC00955F-s001
