# Supplementary material for: Transcription activation by the resistance protein AlbA as a tool to evaluate derivatives of the antibiotic albicidin
Source: Chem Sci. 2023 Apr 17;14(19):5069–78. doi: 10.1039/d3sc00955f (PMC10189885; doi:10.1039/d3sc00955f)
Supplement: SC-014-D3SC00955F-s001 [file SC-014-D3SC00955F-s001.pdf]

Electronic Supplementary Material (ESI) for Chemical Science.
This journal is © The Royal Society of Chemistry 2023

Supplementary Information

Transcription activation by the resistance protein AlbA as a tool to evaluate derivatives of the antibiotic albicidin

Simone Kosol, Lida Rostock, Jonas Barsig, Theresa Tabarelli, Kay Hommernick, Marcel Kulike, Tobias Eulberg, Maria Seidel, Iraj Behroz, Leonardo Kleebauer, Stefan Grätz, Andi Mainz and Roderich D. Süssmuth

Corresponding author: Roderich D Süssmuth
Email: roderich.suessmuth@tu-berlin.de

Supplementary Information 1
1. **Supplementary methods** 2
   1.1. *General experimental details* 2
   1.2. *Bacteria, media and antibiotics* 2
   1.3. *Experimental Methods* 3
   1.4. *Synthesis of albicidin derivatives* 6
2. **Supplementary figures** 31
3. **Supplementary tables** 42
4. **Spectra** 47
5. **References** 71

1. Supplementary methods

1.1. General experimental details

Commercially available reagents (Carl Roth GmbH and Co. KG, Karlsruhe, Germany; Sigma-Aldrich, Taufkirchen, Germany; Iris Biotech GmbH, Marktredwitz, Germany; Orpegen, Heidelberg, Germany; ABCR, Karlsruhe, Germany; Alfa Aesar, Karlsruhe, Germany; Merck, Darmstadt; Germany and Acros, Geel, Belgium) and solvents (Fisher Scientific-Acros, Schwerte, Germany) were used without further purification. If necessary, reactions were carried out under an atmosphere of argon or nitrogen and in dry solvents. Analytical thin layer chromatography was carried out using aluminium-backed plates coated with Macherey-Nagel silica gel (60, F254). Analysis was performed by visualizing under UV light (λ= 254 nm) and by staining with $KMnO_4$ solution (3 g $KMnO_4$, 20 g K_2CO_3, 300 mL dist. water, 5ml NaOH solution (5 %)) and with ninhydrin-solution (ninhydrin (0.3 g), AcOH (3 mL), nBuOH (100 mL). Flash chromatography was carried out with silica gel (particle size 40-63 µm, VWR Chemicals, Darmstadt, Germany). Preparative HPLC was carried out on a 1260 Infinity (Agilent Technologies, Waldbronn, Germany) system with a polymeric reversed phase column (PLRP-S 100A) 300 x 50 mm, particle size 10 µm, Agilent Technologies, Waldbronn, Germany). ^{1}H and ^{13}C spectra were recorded at 298 K using Bruker Avance-II 400 MHz, Bruker Avance-III, 500 MHz or Bruker Avance-III 700 MHz instruments (Bruker, Karlsruhe, Germany). The chemical shifts are reported in ppm using the residual solvent peak (DMSO-d_6, $CDCl_3$ or THF-d_8) as an internal reference. Multiplicity (br = broad singlet, s = singlet, d = doublet, dd = doublet of doublet, t = triplet, q = quartet, m = multiplet) and coupling constants (J = Hz) are quoted where possible. PLC-HRMS spectra were recorded on a QTrap LTQ XL (Thermo Fisher Scientific, Waltham, Massachusetts, USA) with an Agilent 1200 Series HPLC-System (Agilent) Technologies, Waldbronn, Germany) with a C18 column (50 x 2 mm, particle size 3 µm). Luminescence measurements were carried out on a TECAN Infinite 200 Microplate Reader.

1.2. Bacteria, media and antibiotics

E. coli DSM1116, *E. coli* BW25113, *S.* Typhimurium TA100, *B. subtilis* DSM10, *M. luteus* DSM1790, *M. phlei* DSM750 were used for minimal inhibitory concentration (MIC) assays. *E. coli* strain DSM 1116 was used for all susceptibility testing. *E. coli* TOP10 or BL21 Star (DE3) cells (Invitrogen) were used for cloning and protein expression. The plasmids used in this study are listed in table S1. LB medium (Lysogeny broth: 10 g/L peptone, 5 g/L yeast extract, 5 g/L NaCl) was used as broth or in agar plates for all experiments with the exception of MIC assays where MHBII (BBLTM Mueller-Hinton Broth II, Becton, Dickinson and Company, New Jersey, USA) was used. Incubation steps took place at 37°C and 180 rpm shaking if not stated otherwise. Throughout the study, aza-His albicidin (compound **2**) was used as albicidin standard due to its better stability and similar activity compared to natural albicidin[1]. All oligonucleotides and restriction enzymes were purchased from Thermo Scientific and all DNA sequencing reactions were performed by Microsynth AG.

1.3. Experimental Methods

Electrophoretic mobility shift assay (EMSA)

The EMSA assays were performed with purified AlbA and the 41 bp oligonucleotide sequence *pAlbA*, which represents the sequence between the -35 and -10 promoter regions. To obtain the *pAlbA* region, 41 bases long complementary oligonucleotides were purchased and hybridized in hybridization buffer (10 mM Tris-HCl, 50 mM NaCl, 1 mM EDTA, pH 7.5) in a PCR thermocycler to obtain double stranded oligonucleotide by following the protocol of Merck KGaA. As negative control, a randomized region of 41 bp was selected in the *Klebsiella oxytoca* genome, hybridized and used in the same experiments (Figure S1). Purified AlbA with TEV-cleaved His-tag was added in varying concentrations (from 0 to 54 µM) to 0.54 µM of the 41 bp fragments and incubated for 2 h on ice in the dark in reaction buffer (5% glycerol, 40 mM Tris-HCl, 20 mM sodium acetate, 1 mM EDTA, pH 7.8). In case of albicidin addition, the protein-albicidin ratio was set to 1:1.5 to ensure albicidin saturation on the protein (final concentration of 5% (v/v) DMSO in samples). For analysis, 10 µL of each reaction mixture was directly loaded on polyacrylamide gels (separating gel: 9 % acrylamide/5 % bis-acrylamide; stacking gel: 4 % acrylamide/2 % bis-acrylamide). The separation was performed at 250 V over 15 min at 4°C in EMSA running buffer (40 mM Tris-HCl, 20 mM sodium acetate, 1 mM EDTA, pH 7.8). Afterwards, the gels were directly incubated for 30 min in an EtBr solution. Analyzation and documentation of the EtBr gels was performed under UV light at the gel documentation station. To determine the intensity of DNA-bands and to calculate the binding affinity, the gel pictures were evaluated with the software Fiji ImageJ 1.53q using the gel band analysis tool to quantify the intensity for all free DNA bands and background signal. The normalized change of intensity upon addition of protein was plotted and fitted with the Hill equation (1) to determine the binding affinity,

$$Y = B_{max} \frac{[P]^n}{K_d^n + [P]^n} \quad (1)$$

[P] is the protein concentration used in the reaction mixture and n was set to 1. The uncertainty of each measurement point was determined from the normalized background signals in each gel lane.

Cloning of the transcription reporter system

For the reporter plasmid backbone, we chose the vector pCS-PesaRlux[2] which was a gift from Cynthia Collins (Addgene plasmid #47640). Using Gibson assembly[3] the PesaR promoter was removed and the lux reporter gene cassette was replaced with the improved ilux[4] operon from the vector pGEX-iluxCDABE-frp, which was generous gift from Carola Gregor. The ilux operon contains a FMN reductase to faster recycle the necessary co-factor to generate a strongly enhanced luminescence signal[4]. The pCS vector backbone was amplified by PCR using the primers pCS-fw and pCS-rev (Table S5) and the ilux cluster was amplified using the primer pair Ilux-fw and Ilux-rev at annealing temperatures of 64.5 °C and 63.6 °C respectively. The two resulting fragments were purified from gel and incubated at a 1:4 ratio (backbone to ilux cluster fragment) in Gibson mix for one hour at 50°C before transformation of TOP10 cells and selection on LB agar plates with 50 µg/mL kanamycin. A cloning site and ribosomal binding site (RBS) were inserted in the new vector pCS-ilux via site-directed ligase-independent mutagenesis[5] using the primer pairs pCS-MCS-RBS-fwt/pCS-MCS-RBS-rev and pCS-MCS-RBS-fw/pCS-MCS-RBS-revt (annealing temperatures of 61.9 °C and 67.3 °C, respectively), yielding the construct pCS-MCS-RBS-ilux which contained restriction sites for the enzymes BamHI and XhoI upstream of the RBS and ilux cassette (vector map Figure S2A). To generate the reporter plasmids, we then inserted the pAlbA promoter or, as a test system, the T7 promoter. The promoters were obtained as oligonucleotides with *BamHI* and *XhoI* restriction sites adjacent to the promoter regions (see Table S5). The promoter oligonucelotides were dissolved in buffer (10 mM Tris, 50 mM NaCl, 1 mM EDTA, pH 7.5) to reach a concentration of 100 µM and annealed at 95 °C for 5 min followed by slow cooling to RT to yield double-stranded DNA. The promoter fragments and pCS-MCS-RBS-ilux were treated with *BamHI* and *XhoI* according to the manufacturer's protocol, purified using a DNA purification kit (Thermo) and ligated with T4 ligase to yield the plasmids pCS-pAlbA-ilux and pCS-T7-ilux (Figure S2B). The ligation mixtures were added to TOP10 cells and positive clones were selected on LB agar plates with 50 µg/mL kanamycin.

For co-expression with the reporter plasmid, AlbA was cloned into pET15b (Novagen) with an N-terminal His-tag. The AlbA gene was amplified from the expression vector pET28a-AlbAL[6] using the primers AlbA_XhoI_fw and AlbA_BamHI_rev containing the corresponding restriction sites. Restriction cloning was performed as described above and positive clones were on LB agar plates with 100 µg/ml ampicillin. After test measurements suggested that the N-terminal His-tag might affect DNA binding of AlbA, site-directed ligase-independent mutagenesis with the primer pairs deltaHis-AlbA-fwt/deltaHis-AlbA-rev and deltaHis-AlbA-fw/deltaHis-AlbA-revt was performed to yield the expression construct pET15b-ΔHisAlbA. The correct sequence of all constructs was confirmed by plasmid sequencing.

Mutagenesis
All AlbAL mutants were generated using the site-directed ligase-independent mutagenesis strategy[5] with either pET28a-AlbAL[6] or pET15b-ΔHis-AlbA as PCR template. Primer pairs with overhangs containing the mutations were designed for each mutant (Table S6). The ligation mixtures were added to TOP10 cells and positive clones were selected on LB agar plates with 50 µg/mL kanamycin for pET28a constructs and 100 µg/mL ampicillin in case of pET15b based constructs. The correct sequence of all constructs was confirmed by plasmid sequencing.

Protein expression and purification
N-terminally His-tagged AlbA was expressed using a pET28a vector as described previously[6]. In brief, *E. coli* BL21(DE3) cells were transformed with pET28-AlbA plasmid and grown in LB medium with kanamycin. LB broth supplemented with 50 µg/mL kanamycin was inoculated from over-night cultures and grown at 37 °C until an OD_{600} of 0.6 was reached. After induction with 0.2 mM isopropyl β-D-1-thiogalactopyranoside (IPTG) the proteins were expressed at 18 °C for 20 hrs. The cells were harvested by centrifugation (4,000 x G, 20 min), resuspended in lysis buffer (50 mM Tris, pH 8.0, 500 mM NaCl, 10% glycerol) and processed in a cell disruptor (Constant Systems Ltd) at 25 kPsi before centrifugation at 50,000 x G, at 4 °C for 45 min. The lysate was incubated with Pure Cube Ni-NTA agarose (Cube Biotech, Germany) at 4 °C for 20 min while shaking. The slurry was transferred to empty gravity-flow columns (ThermoFisher) and after a washing step with lysis buffer, the protein was eluted in two steps with 250 mM imidazole and 500 mM imidazole in the lysis buffer, respectively. His_6-AlbA was buffer exchanged on PD10 Desalting Columns in assay buffer (50 mM sodium phosphate, pH 7.0, 100 mM NaCl) and the purity of the proteins was assessed on SDS-PAGE (Figure S7). All AlbA mutants were purified following the same protocol. Concentrations were determined with a nano-photometer P330 (Implen, Munich, Germany) spectrophotometer using extinction coefficients calculated with the ProtParam tool[7] before storage at -80 °C.

Transcription activation assay
E. coli BL21(DE3) were transformed with pET15b-AlbA or pET15b-ΔHisAlbA and pCS-pAlbA-ilux. An over-night culture was grown in LB medium with kanamycin and ampicillin to inoculate three 50 mL cultures of BL21(DE3):pET15b-AlbA + pCS-pAlbA-ilux or BL21(DE3):pET15b-ΔHisAlbA + pCS-pAlbA-ilux in LB medium with 50 µg/ml kanamycin and 100 µg/ml ampicillin. The cultures were grown to OD_{600} 0.4-0.6 and transferred to white, clear bottom 96-well plates (Corning Inc.). To 190 µL culture in each well, 10 µl DMSO with albicidin or one of its derivatives were added. In assays to test concentration dependence of the promoter, aza-His albicidin (**2**) was added to reach final concentrations of 0, 0.5, 1.0, 1.5, 2.0 and 5 µM. In transcription response assays with albicidin fragments and albicidin derivatives, 1.5 µM or 2.0 µM final concentration was added of each compound. In assays with ciprofloxacin, novobiocin, acridin orange, Azid MegaStokes, tunicamycin, tetracycline and reserpine, each compound was dissolved in DMSO and added to cultures at final concentrations of 15 µM. Bisbenzimide (Hoechst 33342) was dissolved in DMSO and added to cultures at a final concentration of 5 µM. Sixty points were measured on a TECAN Infinite 200 microplate reader, after orbital shaking for 10 min, with an amplitude of 3 mm, and a wait time of one minute between each measurement. At each time point, the absorbance at 600 nm (9 mm bandwidth and 25 flashes) and the luminescence (500 ms integration time, no attenuation) were measured. Each assay was measured in triplicate at 30 °C. The luminescence data was normalized to the OD before the background signal (wells with no albicidin added) was subtracted and the mean and standard deviation of each measurement point was calculated. Each measurement series was normalized to **2** as a standard, yielding relative luminescence curves of which the maximum within the first five hours of measurement was extracted to compare the luminescence output for each compound.
To compare the transcription activation of AlbA mutants, pET15b-ΔHis-constructs of each mutant together with pCS-pAlbA-ilux were used to transform *E. coli* BL21(DE3). BL21(DE3):pET15b-ΔHisAlbA-mutants + pCS-pAlbA-ilux were grown over-night in 250 µL cultures in 96-well round bottom plates with LB medium with kanamycin and ampicillin (4 replica per mutant per plate) to inoculate an equal number of 250 µL cultures with a starting OD_{600} of 0.02. After six hours, 2 x 100 µL of each culture were transferred to a clear bottom plate and **2** dissolved in 100% DMSO or DMSO was added to a concentration of 1.5 µM (1% DMSO final concentration). Luminescence and absorbance were measured as described above but with 40 time points instead of 60 time points. The measurement was repeated with four more plates, yielding data of 20 biological replica. For each well, the luminescence data was normalized to the OD before the background signal (wells with no albicidin added) was subtracted. The maxima of all twenty luminescence curves of each mutant were determined and differences between the transcription activity of the AlbA mutants and AlbA wild-type were analysed in Origin2022, academic version 9.9.0.225. Wilcoxon-Mann-Whitney tests for statistical evaluation of the data were performed using the Triola Statdisk 13.0 package using the Wilcoxon test for independent samples with a significance of $p \leq 0.01$ or $p \leq 0.05$.

E. coli growth inhibition assay
To test the antibacterial activity of albicidin derivatives in presence and absence of AlbA, susceptibility assays in liquid culture were conducted. An overnight culture of *E. coli* DSM 1116 was used to inoculate 50 mL LB which was grown to an OD ~1.0. The culture was diluted in LB medium to using 0.5 McFarland standard (1.5 x 10^8 cells/mL) and added to a clear round bottom 96-well plate (microdilution tray) with LB to obtain 1 x 10^6 cells/mL. Each well contained 85 µL inocolum to which either 10 µL AlbA (final concentration 10 µM) or buffer (50 mM sodium phosphate pH 7.0, 100 mM NaCl) were added. The albicidin derivatives were dissolved in 100% DMSO and 5 µL were added to the wells to obtain a final concentration of 10 µM compound and 5% (v/v) DMSO in a final culture volume of 100 µL. For the assays with mutants, 10 µM of the mutant proteins were added instead of wild-type AlbA and *E. coli* growth in presence of 10 µM aza-His albicidin (**2**) was measured. All assays were set up in triplicates. The plates were incubated at 37 °C in the dark without shaking for 20 h before documentation using a biostep scanner (Epson) with argusX.

MIC determination
Minimal inhibitory concentration (MIC) values were determined according to the ninth edition of the Approved Standard M07-A9 in microdilution assays. The test was carried out for six different bacterial strains (Gram-negative: *E. coli* DSM1116, *E. coli* BW25113, *S.* Typhimurium TA100; Gram-postive: *B. subtilis* DSM10, *M. luteus* DSM1790, *M. phlei* DSM750). 20 mL LB medium were inoculated with 20 µL of cryo stock of each strain and incubated overnight at 37 °C, 200 rpm shaking. The test inoculum was adjusted by the 0.5 McFarland Standard (OD_{625} from 0.08 to 0.1). Within 15 min of preparation, the adjusted inoculum suspension was diluted in MHBII medium so that each well contained approximately 5 × 10^5 cells/mL in a final volume of 100 µL. 95 µL of the inoculum were applied per well and 5 µL of the albicidin derivative solutions were added. Previously, the dry antibiotic compounds were dissolved in DMSO (100%) with a concentration of 2560 µg/mL and then further diluted in DMSO (100%) for testing. 5 µL of each antibiotic dilution were applied to the microdilution tray to reach final concentrations of 8 µg/mL to 0.016 µg/mL. One row of each 96-well plate served as a growth control without antibiotic substances and another row of the microdilution tray served as sterility control (only MHB II-media). The antimicrobial effect of the solvent (DMSO) was tested by adding 5 µL DMSO to several wells. Purity checks and cell titer controls were performed according to International Standard M07-A9 on Mueller-Hinton II Agar. Both microdilution trays and agar plates were incubated at 37 °C for 20 h and subsequently analyzed by naked eye and documented using a biostep scanner (Epson) with argusX.

DNA-Gyrase inhibition testing
DNA-supercoiling experiments with DNA-gyrase were performed using an *E. coli* gyrase supercoiling kit (Inspiralis Limited, Norwich, UK) following the manufacturer's instructions. A total volume of 30 µL gyrase buffer contained 0.5 µg relaxed pBR322 plasmid DNA (inspiralis Limited), 1 U DNA-gyrase (6 U/µL) (Inspiralis Limited) and the albicidin derivatives **1**, **2** and **10−22** at a final concentration of 45 nM. The final DMSO concentration was 3%. Samples were incubated at 37 °C for 30 min and subsequently loaded on an agarose gel. Electrophoretic analysis was performed using a 1% agarose gel (100 V, 90 min). DNA bands were stained with ethidium bromide, visually analyzed and documented with a Biostep BioView transilluminator and INTAS GelDoc.

CD spectroscopy
The native fold of the purified AlbA mutants was evaluated using CD spectroscopy. The measurements were conducted on a J-815 CD spectrometer (Jasco, Groß-Umstadt, Germany). AlbA and its mutants were diluted in buffer (25 mM sodium hydrogen phosphate, pH 7.0) to reach a concentration of 5 µM. Far-UV spectra were acquired at 20 °C and 30 °C between 190-260 nm with a path length of 0.1 cm and a bandwidth of 1 nm at a scanning speed of 50 nm/min in 5 accumulations. The data pitch was set to 0.1 nm. The spectra were processed with Spectra Manager (JASCO) and the mean residue ellipticity (MRE) was calculated

$$MRE = \frac{\theta * 0.1}{l * c * n} \quad (2)$$

Where θ is the ellipticity and l, c, and n denote the path length, molar concentration and number of amino acids.

Tryptophan fluorescence quenching
Purified His-tagged AlbA and mutant proteins were diluted to a concentration of 50 nM in 200 µl binding assay buffer (50 mM sodium phosphate pH 6.8, 150 mM NaCl). Aza-His albicidin (**2**) solutions with concentrations of 78 nM, 156 nM, 312.5 nM, 625 nM, 2.5 µM, 5 µM, 20 µM and 40 µM were prepared in 100% DMSO. Of each dilution, 2 µl were added to the protein samples (1% DMSO final concentration) before incubation for 20 min at RT in the

dark. The samples were transferred to a Hellma quartz fluorescence cuvette with a path length of 10 mm and tryptophane fluorescence was measured on a PerkinElmer LS 55 fluorescence spectrometer. The excitation was set to λ_{ex} = 282 nm and emission was recorded between 300 nm and 450 nm with a scanning speed of 60 nm s^{-1}. The slit width was set to 8 nm for excitation and 4 nm for emission, respectively. All measurements were performed in triplicate and standard deviations are given for the K_d and Hill factor n. The quenching was determined from the normalized integrated emission band (300-400 nm) of each titration step subtracted from the emission band of the protein without ligand. The normalized tryptophan fluorescence emission was fitted to the equation (3) using Excel Solver 21 and Origin2022:

$$y = B_{max} \frac{[L]^n}{K_D^n + [L]^n} \qquad (3)$$

[L] is the total ligand concentration, n is the Hill coefficient, and B_{max} is the maximum binding capacity of the protein.
To test the binding of the compounds **10**-**22** to AlbA, 50 nM compound were combined with 50 nM AlbA in 200 µl binding assay buffer (with 1% DMSO final concentration). Compound **2** was measured as a control. Tryptophan fluorescence quenching was measured in triplicate as described above after incubation for 20 min at RT in the dark. The quenching was determined from the integrated emission bands (300-400 nm) and calculated as the ratio between free AlbA (I_0) and liganded AlbA (I).

Molecular docking
Using UCSF Chimera[8] and AutoDock Vina[9], AlbAS[6] (PDB-ID: 6et8) was docked with the bisbenzimide structure calculated by pubchem (CID:1464, simulated according to Kim et al.[10]). A monomer molecule of AlbAS without albicidin was prepared as the receptor and docking was conducted using standard parameters with the receptor search volume covering the albicidin binding pocket. The docking results were manually inspected and the highest scoring complex was selected for comparison to the albicidin-AlbA complex.

AlbA structure modelling
AlphaFold v2.1.0[11,12] was used to predict the structure of full-length AlbA. The program was run in the ColabFold[13] notebook (AlphaFold.ipynb) with MMseqs2 and HHsearch to generate sequence alignments and templates and the implemented Amber software to relax the best-ranked structure. For modelling of full-length AlbA monomers, default parameters were used. For modelling dimers, the program was run using template_mode pdb70, the multiple sequence alignments were set to unpaired_paired mode and alphafold2_multimer_v3 was chosen as modelling type with auto settings on recycling parameters.

1.4. Synthesis of albicidin derivatives
Details of the synthesis routes for albicidin and the described albicidin analogues and the identifying MS and NMR spectra are given in the following publications and patents. The total synthesis of albicidin has been described by Kretz et al.[14]. The syntheses of compounds **2**, and **18** have been described by Grätz et al.[15] and compounds **12**, **14**, **15**, **19**, **21** have been described by Behroz et al.[1]. Procedures for the synthesis of albicidin fragments **3**, **4**, **6,** **7** and **9** have been described in Vieweg et al.[16], and compounds **10**, **11** and **13** are described in Michalczyk et al.[17]. The syntheses of the AB-PCP ester and C-terminally allyl-protected tripeptide are described in Behroz et al[1]. Compounds **5**, **8**, **16**, **17**, **20** and **22** were synthesized as follows:

ABCD fragment (5)

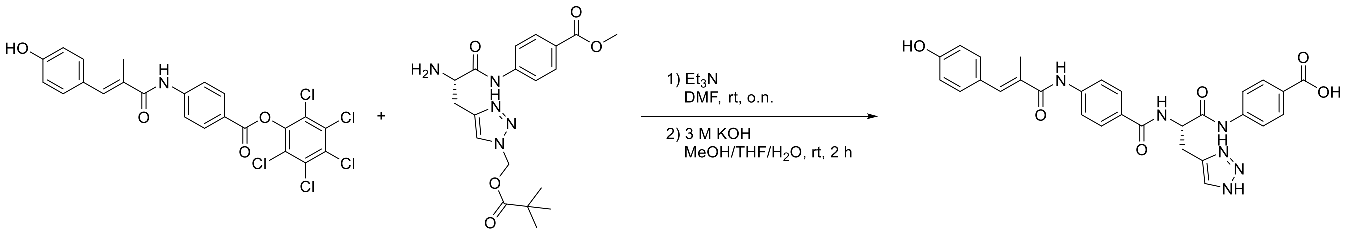

5a **5b** **5**

Scheme 1: Synthesis scheme of ABCD fragment **5** via the coupling of activated AB building block **5a** and dipeptide **5b**.

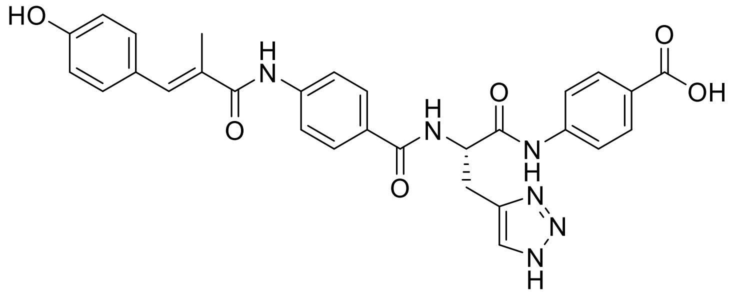

5

$C_{29}H_{26}N_6O_6$

554.1914

The dipeptide **5b** (101 mg, 250 µmol, 1.00 eq.) and PCP activated AB building block[1] (**5a**, 150 mg, 275 µmol, 1.10 eq.) were dissolved in anhydrous DMF and Et_3N (174 µL, 5.0 eq.) was added. The reaction mixture was stirred at room temperature for 16 h. All volatiles were removed in vacuo and the residue was taken up in a mixture of THF and MeOH (1:1 v/v), and 3 M $KOH_{(aq.)}$ was added dropwise at 0 °C. The ice bath was removed and after 15 min of stirring the suspension was acidified to pH ≈ 2 by the addition of 3 M $HCl_{(aq.)}$. The resulting suspension was concentrated under reduced pressure and the crude material was dissolved in DMSO and purified by HPLC (PLRP-S column, CH_3CN in H_2O). BCD fragment **5** (11.2 mg, 7 % over two steps) was obtained as white powder.

^{1}H NMR (DMSO-d$_6$, 500 MHz): δ = 2.11 (1H, s), 2.95 (1H, s), 3.27 (1H, s), 4.89 (1H, s), 6.84 (1H, d, *J* = 7.00 Hz), 7.26 (1H, s), 7.34 (1H, d, *J* = 6.44 Hz), 7.78 (1H, dd, *J* = 16.69 Hz), 7.87 (1H, dd, *J* = 16.00 Hz), 8.72 (1H, s), 9.80 (1H, s), 10.09 (1H, s), 10.57 (1H, s)

H,C-HSQC (DMSO-d$_6$, 126 MHz): δ = 134.2, 131.8, 131.0, 130.7, 128.7, 128.7, 128.2, 119.5, 119.5, 119.1, 119.0, 115.9, 54.9, 45.9, 27.6, 14.9 ppm.

HRMS (ESI): m/z calculated for $C_{29}H_{27}N_6O_6$ $(M+H)^+$ 555.1987, found 555.1986.

BCD fragment (8)

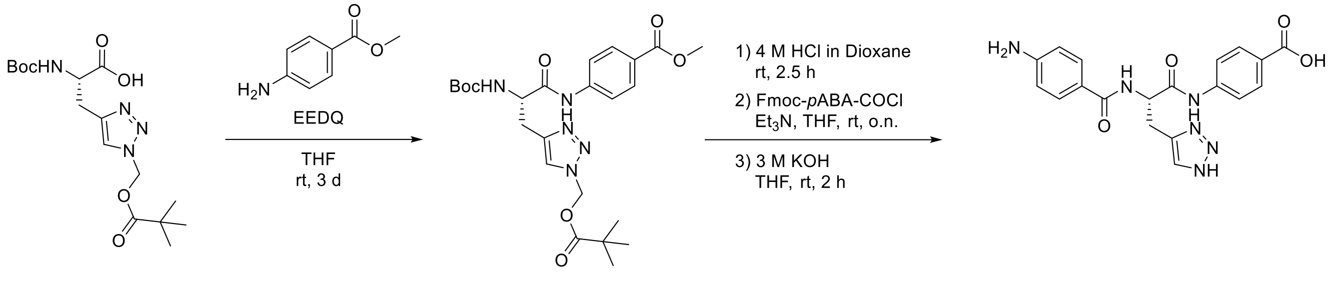

8a **8b** **8**

Scheme 2. Synthesis scheme of BCD fragment **8** starting from the azaHis-C building block **8a**.

Boc(C, Pom)-D-OMe (8a)

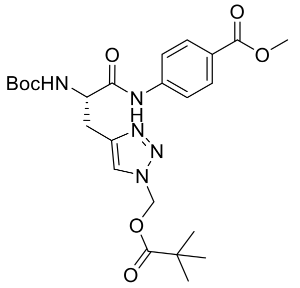

10

$C_{24}H_{33}N_5O_7$

503.2380

To a solution of the amino acid **8a** (800 mg, 2.16 mmol, 1.50 eq.) in dry THF was added EEDQ (570 mg, 2.30 mmol, 1.60 eq.) and the reaction mixture was stirred at room temperature for 15 min. A premixed solution of methyl 4-aminobenzoate (218 mg, 1.44 mmol, 1.00 eq.) in dry THF was added slowly and the reaction mixture was stirred at room temperature for 72 h. The organic solvent was evaporated under reduced pressure and the residue was partitioned between EtOAc and 1 M $HCl_{(aq.)}$. The organic layer was washed with 1 M $HCl_{(aq.)}$ (2x), brine, dried over anhydr. Na_2SO_4, and concentrated *in vacuo* to afford the crude product, which was purified by column chromatography (SiO_2, Hexane/EtOAc – 5:1). Compound **8b** (310 mg, 43 %) was obtained as yellow solid.

^{1}H NMR (DMSO-d$_6$, 500MHz): δ = 1.09 (1H, s), 1.36 (1H, s), 3.00 (1H, t, *J* = 7.30 Hz), 3.10 (1H, d, *J* = 4.85 Hz), 3.83 (1H, s), 4.40 (1H, d, *J* = 5.17 Hz), 6.28 (1H, s), 7.20 (1H, d, *J* = 7.92 Hz), 7.74 (1H, d, *J* = 8.79 Hz), 7.92 (1H, d, *J* = 8.80 Hz), 7.97 (1H, s), 10.44 (1H, s)

^{13}C NMR (DMSO-d$_6$, 126 MHz): δ = 176.9, 171.3, 166.3, 155.8, 143.7, 130.7, 124.5, 119.2, 78.8, 70.3, 55.4, 52.3, 28.6, 26.9 ppm.

HRMS (ESI): m/z calculated for $C_{43}H_{42}N_9O_{12}$ $(M+H)^+$ 504.2453, found 504.2446.

BCD fragment (8)

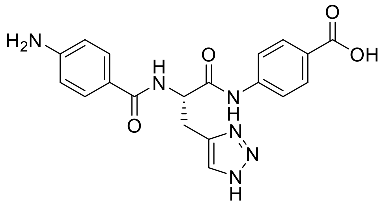

$C_{19}H_{18}N_6O_4$
394.1390

The dipeptide **8b** (250 mg, 497 µmol, 1.00 eq.) was dissolved in 4 M HCl in 1,4-dioxane and stirred at room temperature for 2.5 h. Subsequently, all volatiles were removed under reduced pressure to obtain the crude product, which was taken up in H_2O/CH_3CN and freeze-dried to afford the Boc-deprotected product as slightly yellow powder. The unprotected dipeptide (114 mg, 284 µmol, 1.00 eq.) and Fmoc-protected acid chloride (150 mg, 397 µmol, 1.40 eq.) were dissolved in anhydrous THF and Et_3N (395 µL, 10.0 eq.) was added. The reaction mixture was stirred at room temperature for 16 h. All volatiles were removed *in vacuo* and the residue was taken up in a mixture of THF and MeOH (1:1 v/v), and 3 M $KOH_{(aq.)}$ was added dropwise at 0 °C. The ice bath was removed and after 15 min of stirring the suspension was acidified to pH ≈ 2 by the addition of 3 M $HCl_{(aq.)}$. The resulting suspension was concentrated under reduced pressure and the crude material was dissolved in DMSO and purified by HPLC (PLRP-S column, CH_3CN in H_2O). BCD fragment **8** (7.53 mg, 7 % over three steps) was obtained as white powder.

^{1}H NMR (DMSO-d$_6$, 500MHz): δ = 3.23 (1H, s), 4.63 (1H, s), 4.84 (1H, d, *J* = 0.69 Hz), 6.68 (1H, d, *J* = 0.38 Hz), 7.66 (1H, s), 7.73 (1H, s), 7.87 (1H, s), 8.40 (1H, s), 10.51 (1H, s)

H,C-HSQC (DMSO-d$_6$, 126 MHz): δ = 130.6, 129.4, 118.9, 114.4, 54.7, 27.6 ppm.

HRMS (ESI): m/z calculated for $C_{19}H_{19}N_6O_4$ $(M+H)^+$ 395.1462, found 395.1458.

Bipyridyl-iso-propoxy-albicidin (16)

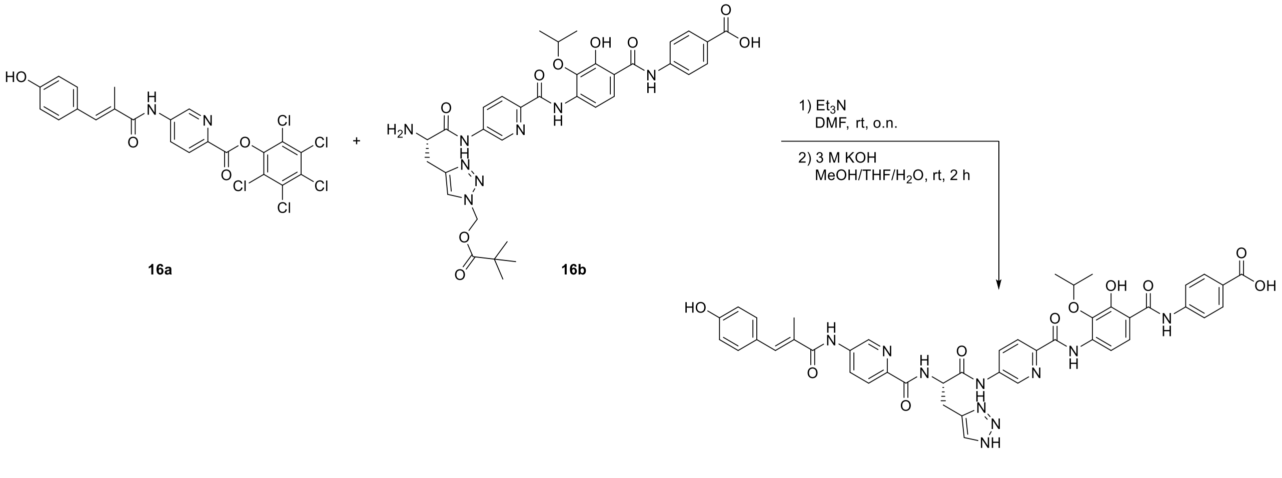

Scheme 3: Synthesis of derivative **16** from tetrapeptide **16b** with activated AB building block**16a**.

B(Pyr)-C(azaHis)-D(Pyr)-E(*i*Pr)-F(*p*ABA) (16)

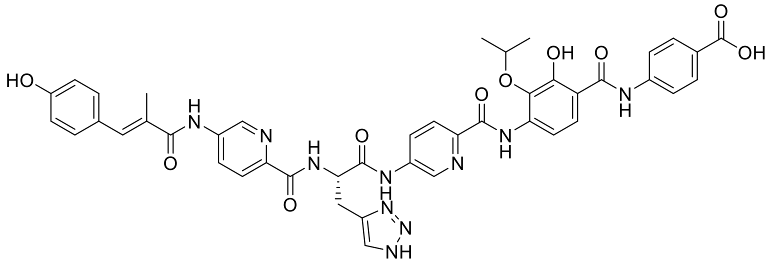

$C_{44}H_{40}N_{10}O_{10}$
868.2929

The Tetrapeptide **16b**[17] (100 mg, 135 µmol, 1.00 eq.) and PCP activated AB building block[18] (**16a**, 81.3 mg, 149 µmol, 1.10 eq.) were dissolved in anhydrous DMF and Et_3N (132 µL, 7.0 eq.) was added. The reaction mixture was stirred at room temperature for 16 h. All volatiles were removed *in vacuo* and the residue was taken up in a mixture of THF and MeOH (1:1 v/v), and 3 M $KOH_{(aq.)}$ was added dropwise at 0 °C. The ice bath was removed and after 15 min of stirring the suspension was acidified to pH ≈ 2 by the addition of 3 M $HCl_{(aq.)}$. The resulting suspension was concentrated under reduced pressure and the crude material was dissolved in DMSO and purified by HPLC (PLRP-S column, CH_3CN in H_2O). Derivative **16** (9.98 mg, 8% over two steps) was obtained as white powder.

^{1}H NMR (DMSO-d$_6$, 500MHz): δ = 1H-NMR (DMSO) 1.35 (6H, d, *J* = 1.36 Hz), 2.14 (3H, s), 3.37 (3H, d, *J* = 6.08 Hz), 4.68 (1H, t, *J* = 6.06 Hz), 5.00 (1H, d, *J* = 7.24 Hz), 6.85 (2H, d, *J* = 8.56 Hz), 7.36 (3H, t, *J* = 10.22 Hz), 7.86 (2H, d, *J* = 8.68 Hz), 7.93 (1H, s), 7.97 (2H, d, *J* = 8.64 Hz), 8.02 (1H, d, *J* = 8.56 Hz), 8.11 (1H, d, *J* = 8.92 Hz), 8.20 (1H, d, *J* = 8.60 Hz), 8.27 (1H, d, *J* = 1.92 Hz), 8.34 (1H, dd, *J* = 3.60 Hz), 8.87 (1H, d, *J* = 7.96 Hz), 8.99 (2H, d, *J* = 2.84 Hz), 9.83 (1H, s), 10.38 (2H, s), 10.62 (1H, s), 10.76 (1H, s), 10.85 (1H, s), 12.46 (1H, s)

H,C-HSQC (DMSO-d$_6$, 126 MHz): δ = 140.4, 135.1, 132.1, 131.9, 130.7, 128.0, 127.8, 127.6, 124.2, 124.0, 123.5, 123.2, 122.8, 122.8, 121.3, 121.3, 116.0, 109.0, 75.2, 53.6, 28.0, 22.7, 14.8 ppm.

***HRMS** (ESI): m/z calculated for $C_{44}H_{41}N_{10}O_{10}$ (M+H)$^+$ 869.3002, found 869.2996*

Ether-C-D-Isostere (17)

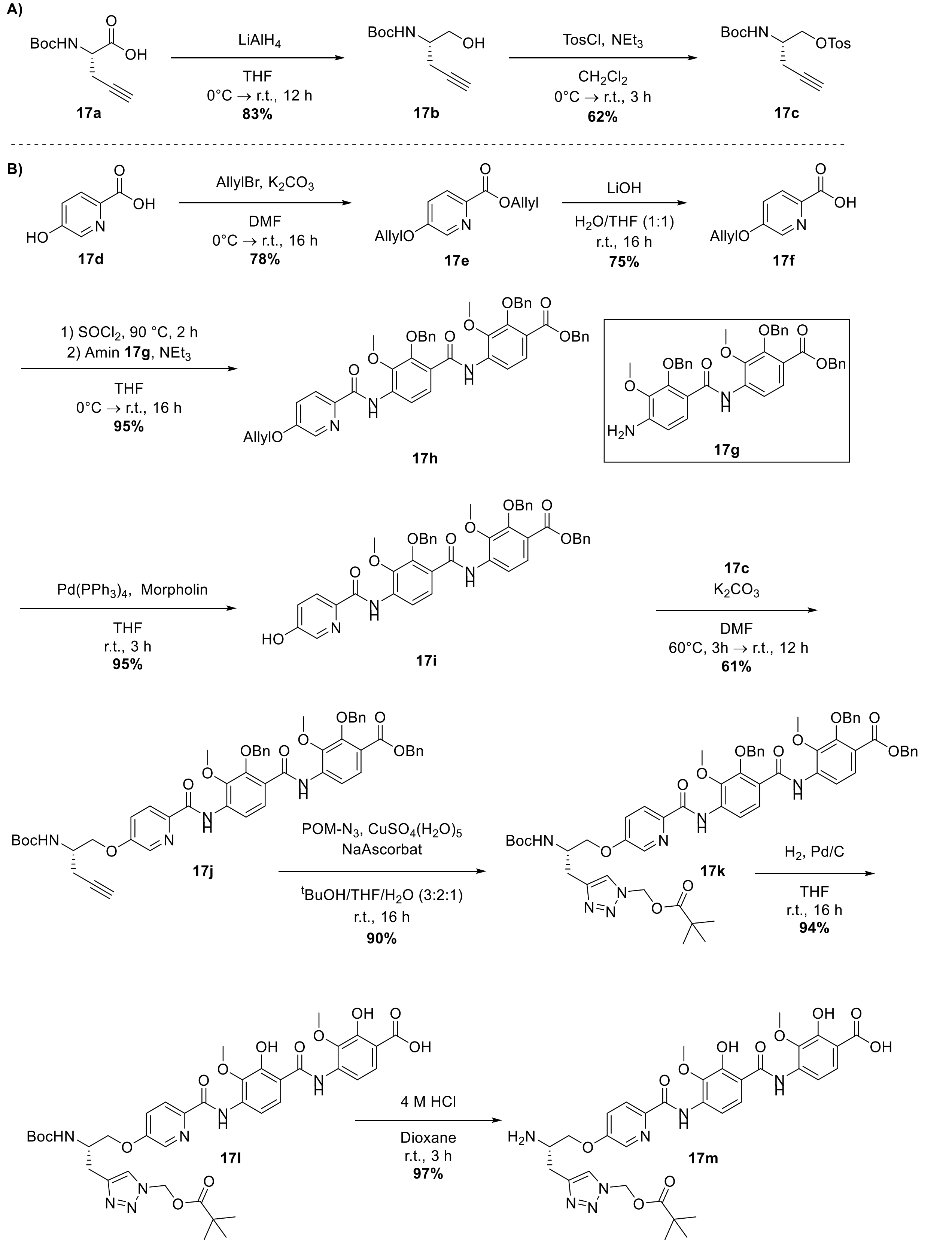

Scheme 4: **A)** Synthesis of tosylate **17c** starting from Boc-propargylglycine (**17a**) **B)** Synthesis of fragment **17m** starting from picolinic acid **17d**.

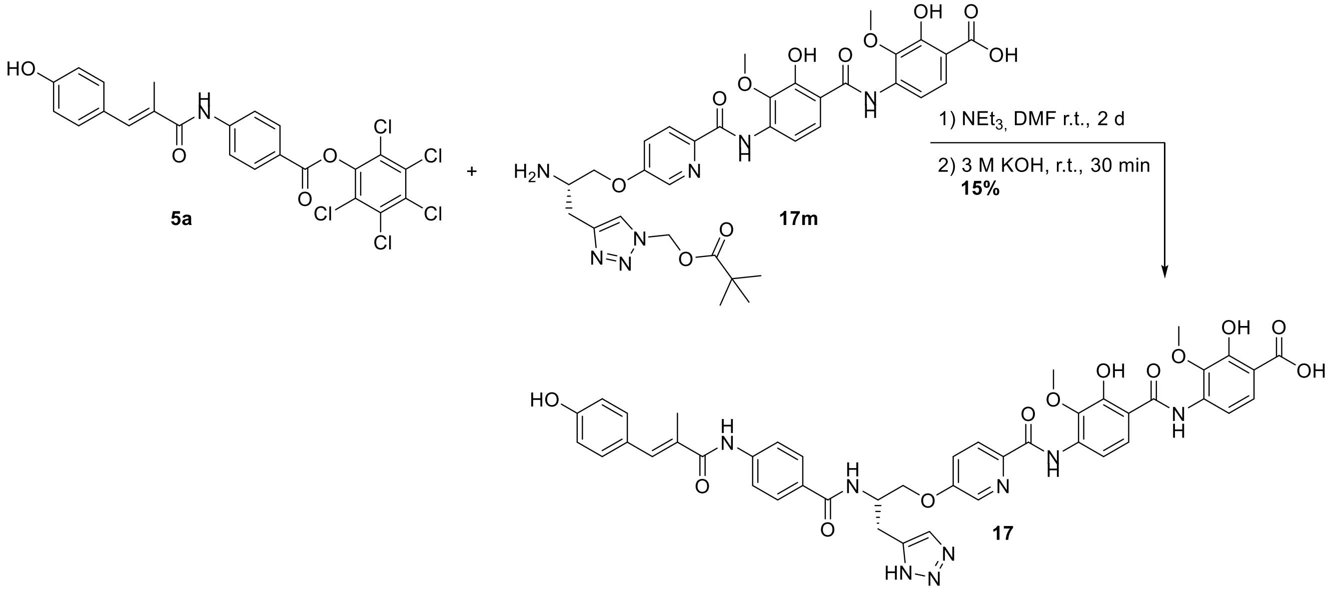

Scheme 5: Final coupling of activated AB building block **5a** with fragment **17m** giving ether-C-D-isostere **17**.

tert-Butyl-(_S_)-(1-hydroxypent-4-yn-2-yl)carbamate (17b)

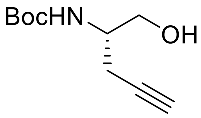

Lithium aluminium hydride (3.56 g, 93.8 mmol, 2.00 eq.) was added to dry THF (100 mL) at 0°C under a N_2-atmosphere. To the reaction mixture was added a solution of (*S*)-2-((*tert*-butoxycarbonyl)amino)pent-4-inic acid (**17a**, 10.0 g, 46.9 mmol, 1.00 eq.) in THF (20 mL) over a period of 20 min. The mixture was stirred for 12 h at room temperature until the reaction was completed. The reaction mixture was diluted with ethyl acetate (50 mL), quenched by the addition of water (50 mL) and filtrated over Celite®. The mixture was extracted by ethyl acetate (3 x 30 mL) and washed by water (30 mL). The combined organic phases were dried over Na_2SO_4, filtered and the solvent was removed by rotary evaporation. The product **17b** (7.78 g, 77.9 mmol, 83 %) was obtained as white solid and used in the next step without further purification. The protocol was based on a literature known procedure used for the synthesis of a similar product[19].

^{1}H NMR (500 MHz, DMSO-*d*$_6$) δ 6.57 (d, *J* = 8.4 Hz, 1H), 4.69 (t, *J* = 5.7 Hz, 1H), 3.37 - 3.41 (m, 1H), 3.30 - 3.34 (m, 1H), 2.73 (s, 1H), 2.36 (ddd, *J* = 16.6, 6.1, 2.7 Hz, 1H), 2.21 (ddd, *J* = 16.7, 7.1, 2.7 Hz, 1H), 1.38 (s, 9H).

^{13}C NMR (126 MHz, DMSO) δ 155.1, 81.9, 77.7, 72.0, 62.1, 51.6, 28.2, 20.6.

HRMS (ESI-ORBITRAP): *m/z* [M+H]$^+$ calc. for $C_{10}H_{18}NO_3$: 200.1281; found: 200.1285.

(*S*)-2-((*tert*-butoxycarbonyl)amino)pent-4-yn-1-yl4-methylbenzene sulfonate (17c)

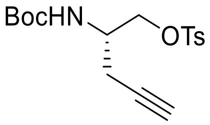

Triethylamine (9.12 mL, 65.2 mmol, 2.00 eq.) and *p*-toluenesulfonyl chloride (7.46 g, 39.1 mmol, 1.20 eq.) were added to a solution of alcohol **17b** (6.50 g, 32.6 mmol, 1.00 eq.) in CH_2Cl_2 (50 mL) at 0 °C. The reaction mixture was stirred for 3 h at room temperature and after completion of the reaction the volatiles were removed by rotary evaporation. The residue was taken up by CH_2Cl_2 (50 mL) and washed by an aqueous solution of HCl (1 M, 2 x 30 mL) and a saturated aqueous solution of NaCl (30 mL). The organic phase was dried over Na_2SO_4, filtered and concentrated by rotary evaporation. The crude product was purified by column chromatography (SiO_2, 20:1 → 7:1, cyclohexane:ethyl acetate) and the product (**17c**, 7.11 g, 20.2 mmol, 62 %) was obtained as a yellow solid. The protocol was based on a literature known procedure used for the synthesis of a similar product[20].

^{1}H NMR (500 MHz, DMSO-*d*$_6$) δ 7.78 (d, *J* = 8.3 Hz, 2H), 7.48 (d, *J* = 8.1 Hz, 2H), 7.00 (d, *J* = 8.3 Hz, 1H), 3.98 (d, *J* = 5.3 Hz, 2H), 3.71 (m, 1H), 2.81 (s, 1H), 2.42 (s, 3H), 2.28 - 2.31 (m, 2H), 1.35 (s, 9H).

^{13}C NMR (126 MHz, DMSO-*d*$_6$) δ 154.9, 145.0, 132.0, 130.1, 127.6, 80.3, 78.1, 72.9, 70.1, 50.1, 48.7, 28.1, 21.1.

HRMS (ESI-ORBITRAP): *m/z* $[M+H]^+$ calc. for $C_{17}H_{24}NO_5S$: 354.1370; found 354.1372.

Allyl-5-(allyloxy)picolinate (17e)

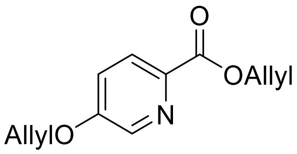

To a suspension of potassium carbonate (2.61 g, 21.6 mmol, 3.00 eq.) in DMF (10 mL) was added allyl bromide (1.86 mL, 21.6 mmol, 3.00 eq.) and a solution of 5-hydroxypicolinic acid (**17d**, 1.00 g, 7.19 mmol, 1.00 eq.) in DMF (5 mL) was added dropwise at 0 °C. The reaction mixture was stirred for 16 h at room temperature, after completion of the reaction diluted by water (50 mL) and extracted by diethyl ether (4 x 30 mL). The combined organic phases were concentrated by rotary evaporation and the product (**17e**, 1.23 g, 16.8 mmol, 78 %) was obtained as orange oil and used without further purification in the next step.

^{1}H NMR (500 MHz, DMSO-*d*$_6$) δ 8.41 (d, *J* = 2.9 Hz, 1H), 8.05 (d, *J* = 8.7 Hz, 1H), 7.53 (dd, *J* = 8.8, 3.0 Hz, 1H), 6.12 – 5.98 (m, 2H), 5.42 (ddd, *J* = 18.9, 17.2, 1.7 Hz, 2H), 5.32 (ddd, *J* = 10.6, 1.4 Hz, 1H), 5.27 (ddd, *J* = 10.5, 1.5 Hz, 1H), 4.80 (dt, *J* = 5.5, 1.5 Hz, 2H), 4.75 (dt, *J* = 5.3, 1.5 Hz, 2H).

^{13}C NMR (126 MHz, DMSO-*d*$_6$) δ 163.9, 156.9, 139.6, 138.6, 132.6, 132.6, 126.4, 121.0, 118.4, 118.0, 68.8, 65.1.

HRMS (ESI-ORBITRAP): *m/z* $[M+H]^+$ calc. for $C_{12}H_{14}NO_3$: 220.0968; found: 220.0961.

5-(Allyloxy)picolinic acid (17f)

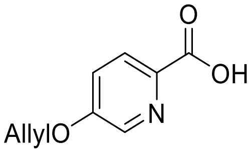

To a solution of allylester (**17e**, 1.23 g, 5.63 mmol, 1.00 eq.) in THF (10 mL) was added a solution of lithium hydroxide (943 mg, 39.4 mmol, 7.00 eq.) in water (10 mL) and the reaction mixture was stirred for 16 h at room temperature. After completion of the reaction the organic solvent was removed by rotary evaporation and the residue was extracted by CH_2Cl_2 (5 x 10 mL). The combined organic phases were concentrated by rotary evaporation and the product (**17f**, 757 mg, 4.22 mmol, 75 %) was obtained as orange solid.

^{1}H NMR (500 MHz, DMSO-*d*$_6$) δ 12.84 (s, 1H), 8.38 (d, *J* = 2.9 Hz, 1H), 8.01 (d, *J* = 8.7 Hz, 1H), 7.51 (dd, *J* = 8.7, 2.9 Hz, 1H), 6.06 (ddt, *J* = 17.2, 10.5, 5.3 Hz, 1H), 5.44 (ddd, *J* = 17.3, 1.7 Hz, 1H), 5.31 (ddd, *J* = 10.5, 1.4 Hz, 1H), 4.74 (dt, *J* = 5.3, 1.5 Hz, 2H).

^{13}C NMR (126 MHz, DMSO-*d*$_6$) δ 165.7, 156.7, 140.5, 138.0, 132.7, 126.1, 121.1, 118.4, 68.8.

HRMS (ESI-ORBITRAP): *m/z* $[M+H]^+$ calc. for $C_9H_{10}NO_3$: 180.0655; found: 180.0659.

Allyloxy-D(N)-E(OBn)-F(OBn, OBn) (17h)

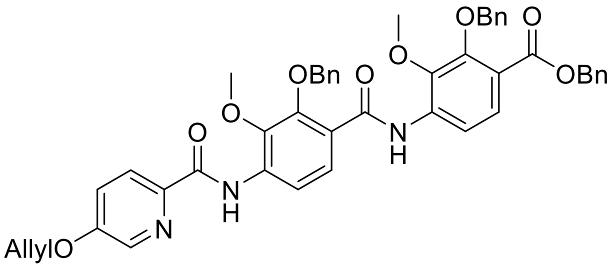

Carboxylic acid (**17f**, 525 mg, 2.93 mmol, 1.20 eq.) was suspended in thionyl chloride (5 mL) and stirred for 2 h at 90 °C. The reaction mixture was allowed to cool down to room temperature and the volatiles were removed by rotary evaporation. The residue was taken up in dry THF (5 mL) and added to a cooled solution of benzyl protected dipeptide **17g**[1] (1.51 g, 2.44 mmol, 1.00 eq.) and triethylamine (680 µL, 4.88 mmol, 2.00 eq.) in dry THF (5 mL) at 0 °C and stirred for 16 h at room temperature. To the reaction mixture was added diethyl ether (70 mL), the precipitated product was filtered and the solid was dissolved in CH_2Cl_2 (30 mL). The organic phase was washed by an aqueous solution of HCl (1 M, 2 x 30 mL) and a saturated aqueous solution of NaCl (30 mL). The organic phase was dried over Na_2SO_4, filtered and concentrated by rotary evaporation. Tripeptide (**17h**, 1.80 g, 2.78 mmol, 95 %) was obtained as yellowish solid.

DC: (cyclohexane/ethyl acetate = 2:1, v/v), R_f = 0.55.

^{1}H NMR (500 MHz, DMSO-*d*$_6$) δ 10.58 (s, 1H), 10.55 (s, 1H), 9.58 (s, 1H), 8.49 (d, *J* = 2.8 Hz, 1H), 8.39 (d, *J* = 8.8 Hz, 1H), 8.27 (d, *J* = 8.8 Hz, 1H), 8.19 (d, *J* = 8.8 Hz, 1H), 7.81 (d, *J* = 8.9 Hz, 1H), 7.67 (dd, *J* = 8.8, 2.8 Hz, 1H), 7.58 (d, *J* = 8.8 Hz, 1H), 7.43 (td, *J* = 6.0, 3.2 Hz, 4H), 7.41 – 7.32 (m, 8H), 7.30 (dd, *J* = 4.9, 1.9 Hz, 3H), 6.09 (ddt, *J* = 17.4, 10.7, 5.3 Hz, 1H), 5.47 (ddd, *J* = 17.2, 1.7 Hz, 1H), 5.38 – 5.28 (m, 5H), 4.95 (s, 2H), 4.80 (dt, *J* = 5.3, 1.6 Hz, 2H), 4.03 (s, 3H), 3.62 (m, 4H).

HRMS (ESI-ORBITRAP): *m/z* $[M+H]^+$ calc. for $C_{46}H_{42}N_3O_9$: 780.2916; found: 780.2928.

Hydroxy-D(N)-E(OBn)-F(OBn, OBn) (17i)

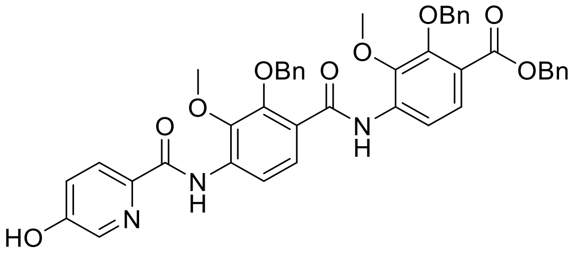

To a solution of allyl-protected alcohol (**17h**, 1.81 g, 2.32 mmol, 1.00 eq.) in dry THF (10 mL), morpholine (4.00 mL, 46.2 mmol, 20.0 eq.) and $Pd(PPh_3)_4$ (536 mg, 464 µmol, 0.20 eq.) were added and the reaction was stirred for 3 h at room temperature. After completion of the reaction the volatiles were removed by rotary evaporation and the residue was taken up with ethyl acetate. The organic phase was washed by an aqueous solution of HCl (1 M, 2 x 30 mL) and a saturated aqueous solution of NaCl (30 mL). The organic phase was dried over Na_2SO_4, filtered and concentrated by rotary evaporation. The crude product was purified by column chromatography (SiO_2, 10 % → 40 % ethyl acetate in cyclohexane) alcohol (**17i**, 1.13 g, 1.53 mmol, 66 %) was obtained as yellowish solid.

DC: (cyclohexane/ethyl acetate = 2:1, v/v), R_f = 0.29.

^{1}H NMR (500 MHz, DMSO-d_6) δ 10.96 – 10.92 (m, 1H), 10.55 (s, 2H), 8.39 (d, *J* = 7.9 Hz, 1H), 8.27 (d, *J* = 8.9 Hz, 2H), 8.09 (d, *J* = 8.5 Hz, 1H), 7.81 (d, *J* = 8.8 Hz, 1H), 7.58 (d, *J* = 8.7 Hz, 1H), 7.44 (d, *J* = 8.3 Hz, 5H), 7.39 – 7.35 (m, 8H), 7.30 (d, *J* = 4.0 Hz, 3H), 7.29 (s, 1H), 5.31 (d, *J* = 6.6 Hz, 4H), 4.94 (s, 2H), 4.03 (s, 3H), 3.63 (d, *J* = 3.2 Hz, 3H).

^{13}C NMR (126 MHz, DMSO-d_6) δ 164.6, 162.3, 161.8, 157.2, 151.1, 149.4, 142.5, 141.7, 140.0, 137.2, 136.8, 136.1, 136.1, 135.5, 129.0, 128.6, 128.5, 128.3, 128.3, 128.2, 128.1, 128.0, 126.4, 124.0, 121.9, 114.9, 114.3, 76.3, 75.3, 66.2, 61.3, 60.7.

HRMS (ESI-ORBITRAP): *m/z* $[M+H]^+$ calc. for $C_{43}H_{38}N_3O_9$: 740.2603; found: 740.2606.

BocHN-CH(propargyl)-CH_2O-D(N)-E(OBn)-F(OBn, OBn) (17j)

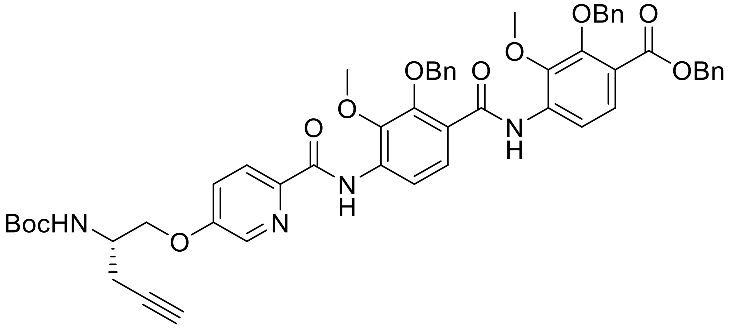

Chemical Formula: $C_{53}H_{52}N_4O_{11}$
Exact Mass: 920.3633

To a solution of phenol (**17i**, 621 mg, 839 µmol, 1.00 eq.) in DMF (10 mL) was added potassium carbonate (232 mg, 1.68 mmol, 2.00 eq.) and amino acid (**17c**, 1.48 g, 4.20 mmol, 5.00 eq.). The reaction mixture was stirred for 3 h at 60 °C and then 12 h at room temperature. The volatiles were removed by rotary evaporation and the crude product was purified with column chromatography (SiO_2,10 % → 25 % ethyl acetate in cyclohexane). The product (**17j**, 470 mg, 512 µmol, 61 %) was obtained as yellowish solid.

DC: (cyclohexane/ethyl acetate = 2:1, v/v), R_f = 0.26.

^{1}H NMR (500 MHz, DMSO-d_6) δ 10.57 (s, 1H), 10.55 (s, 1H), 8.46 (d, *J* = 2.8 Hz, 1H), 8.39 (d, *J* = 8.8 Hz, 1H), 8.27 (d, *J* = 8.8 Hz, 1H), 8.18 (d, *J* = 8.7 Hz, 1H), 7.81 (d, *J* = 8.8 Hz, 1H), 7.67 (dd, *J* = 8.7, 2.8 Hz, 1H), 7.58 (d, *J* = 8.9 Hz, 1H), 7.47 – 7.43 (m, 3H), 7.27 - 7.43 (m, 15H), 7.12 (d, *J* = 8.2 Hz, 1H), 5.31 (d, *J* = 8.3 Hz, 4H), 4.95 (s, 2H), 4.18 (dt, *J* = 9.7, 5.5 Hz, 2H), 4.03 (s, 3H), 3.95 (h, *J* = 8.4, 7.4 Hz, 1H), 3.63 (s, 3H), 2.89 (d, *J* = 2.6 Hz, 1H), 1.39 (s, 9H).

(^{1}H,^{13}C)-HSQC_ed NMR (126 MHz, DMSO-d_6) δ 137.7, 114.8, 115.3, 124.2, 127.0, 122.7, 126.9, 129.2, 128.8, 128.9, 76.7, 66.7, 75.8, 69.8, 61.7, 49.5, 61.1, 51.9, 62.7, 73.8, 28.8.

HRMS (ESI-ORBITRAP): *m/z* $[M+H]^+$ calc. for $C_{53}H_{53}N_4O_{11}$; 921.3705 found: 921.3705.

Boc-azaHis(POM)-D(N)-E(OBn)-F(OBn, OBn) (17k)

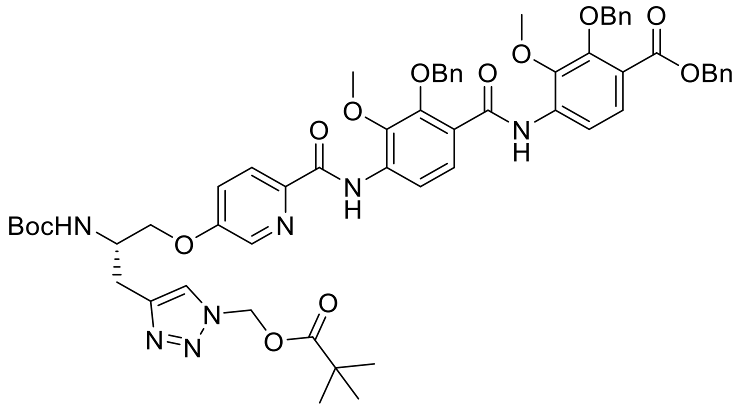

Chemical Formula: $C_{59}H_{63}N_7O_{13}$
Exact Mass: 1077.4484

To a solution of alkyne (**17j**, 470 mg, 510 µmol, 1.00 eq.) in *tert*-butanol (30 ml), THF (20 mL) und water (10 ml) was added copper(II)-sulfate-pentahydrate (12.7 mg, 51.0 mmol, 0.100 eq.). A stream of N_2-gas was bubbled through the solution for 5 min. Sodium ascorbate (40.4 mg, 204 µmol, 0.40 eq.) was added to the reaction mixture which was stirred for 16 h at room temperature. After completion of the reaction, the solvent was removed by rotary evaporation and the residue was diluted with water (30 mL). The mixture was extracted by ethyl acetate (3 x 50 mL) and the combined organic phases were washed by a saturated aqueous solution of NaCl (30 mL). The organic phase was dried over Na_2SO_4, filtered and concentrated by rotary evaporation. The crude product was purified by column chromatography (SiO_2, 25 % → 50 % ethyl acetate in cyclohexane). The product was obtained as colorless solid (**17k**, 442 mg, 459 µmol, 90 %).

DC: (cyclohexane/ethyl acetate = 1:1, v/v), R_f = 0.29.

^{1}H NMR (500 MHz, DMSO-*d*6) δ 10.57 (s, 1H), 10.55 (s, 1H), 8.45 (d, *J* = 2.9 Hz, 1H), 8.39 (d, *J* = 8.8 Hz, 1H), 8.27 (d, *J* = 8.8 Hz, 1H), 8.18 (d, *J* = 8.7 Hz, 1H), 7.99 (d, *J* = 1.6 Hz, 1H), 7.81 (d, *J* = 8.8 Hz, 1H), 7.65 (dd, *J* = 8.7, 2.9 Hz, 1H), 7.58 (d, *J* = 8.8 Hz, 1H), 7.47 – 7.27 (m, 19H), 7.07 (d, *J* = 8.4 Hz, 1H), 6.28 (s, 2H), 5.32 (s, 2H), 5.30 (s, 2H), 4.95 (s, 2H), 4.16 (d, *J* = 5.8 Hz, 2H), 4.12 – 4.07 (m, 1H), 4.03 (s, 3H), 3.63 (s, 3H), 3.02 (dd, *J* = 14.7, 5.4 Hz, 1H), 2.89 (dd, *J* = 14.8, 8.5 Hz, 1H), 1.35 (s, 9H), 1.11 (s, 9H).

(^{1}H,^{13}C)-HSQC_ed NMR (126 MHz, DMSO-*d*6) δ 137.6, 114.9, 115.3, 124.2, 124.1, 127.1, 122.8, 126.9, 129.0, 128.8, 128.9, 76.8, 66.7, 75.9, 70.4, 50.0, 61.7, 61.1, 27.6, 28.6, 26.9.

HRMS (ESI-ORBITRAP): *m/z* $[M+H]^+$ calc. for $C_{59}H_{64}N_7O_{13}$: 1078.4557; found: 1078.4564.

Boc-azaHis(POM)-D(N)-E-F (17l)

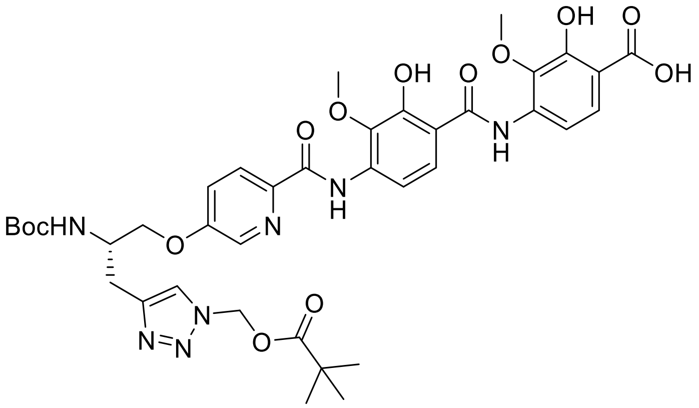

Chemical Formula: $C_{38}H_{45}N_7O_{13}$
Exact Mass: 807.3075

Tetrapeptide (**17k**, 442 mg, 410 µmol, 1.00 eq.) was dissolved in THF (10 mL) and a stream of N_2-gas was bubbled through the solution for 5 min. Palladium on charcoal (10 wt. %, 44.2 mg) was added to the reaction mixture which was stirred for 16 h at room temperature. After completion of the reaction the mixture was filtered over Celite® and the filtrate was concentrated by rotary evaporation. The product (**17l**, 311 mg, 385 µmol, 94 %) was obtained as colorless solid.

^{1}H NMR (400 MHz, DMSO-d_6) δ 11.72 (s, 2H), 11.13 (s, 1H), 10.44 (s, 1H), 8.44 (d, J = 2.8 Hz, 1H), 8.17 (d, J = 8.7 Hz, 1H), 8.11 (d, J = 9.0 Hz, 1H), 8.02 (d, J = 8.9 Hz, 2H), 7.99 (s, 1H), 7.87 (d, J = 8.9 Hz, 1H), 7.65 (dd, J = 8.9, 2.9 Hz, 1H), 7.59 (d, J = 8.9 Hz, 1H), 7.20 (dd, J = 7.6 Hz, 27.2, 2H), 7.09 (d, J = 8.3 Hz, 1H), 6.28 (s, 2H), 4.18 – 4.05 (m, 3H), 3.91 (s, 3H), 3.86 (s, 3H), 3.02 (dd, J = 14.7, 5.3 Hz, 1H), 2.88 (dd, J = 14.7, 8.3 Hz, 1H), 1.35 (s, 9H), 1.10 (s, 9H).

(^{1}H,^{13}C)-HSQC_ed NMR (101 MHz, DMSO-d_6) δ 137.5, 124.1, 110.7, 124.1, 126.9, 122.8, 125.9, 70.2, 70.2, 49.6, 60.6, 61.2, 29.0, 29.0, 26.9.

HRMS (ESI-ORBITRAP): m/z [M+H]$^+$ calc. for $C_{38}H_{46}N_7O_{13}$: 808.3148; found: 808.3148.

AzaHis-C(POM)-D(N)-E-F (17m)

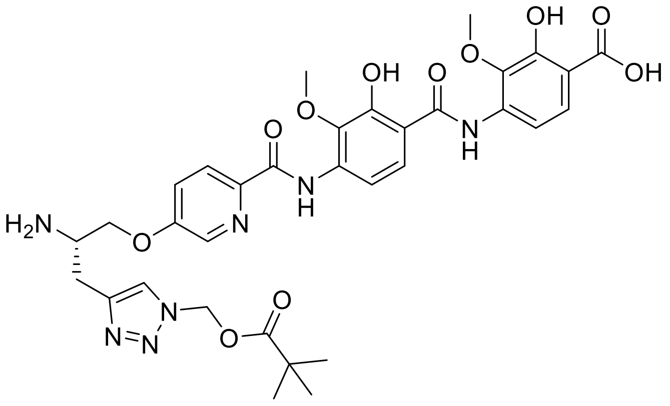

Chemical Formula: $C_{33}H_{37}N_7O_{11}$
Exact Mass: 707.2551

Tetrapeptide (**17l**, 331 mg, 410 µmol, 1.00 eq) was suspended in a solution of 4 M HCl in 1,4-dioxane (5 mL) and the reaction mixture was stirred for 3 h at room temperature. After completion of the reaction, the volatiles were removed by rotary evaporation, the residue was taken up by water and acetonitrile and lyophilized. The amine (**17m**, 281 mg, 398 µmol, 97 %) was obtained as white solid.

^{1}H NMR (500 MHz, DMSO-d_6) δ 11.72 (br. S, 1H), 11.11 (s, 1H), 10.44 (s, 1H), 8.74 – 8.71 (m, 1H), 8.67 (s, 1H), 8.49 (d, J = 2.8 Hz, 1H), 8.23 – 8.16 (m, 1H), 8.15 – 8.07 (m, 1H), 8.01 (d, J = 8.9 Hz, 1H), 7.87 (d, J = 8.9 Hz, 1H), 7.82 (d, J = 32.0 Hz, 1H), 7.68 (dd, J = 8.8, 2.8 Hz, 1H), 7.58 (d, J = 8.8 Hz, 1H), 5.56 (s, 2H), 4.43 (dt, J = 9.7, 3.3 Hz, 1H), 4.31 (dt, J = 11.9, 5.9 Hz, 1H), 3.91 (s, 3H), 3.86 (s, 3H), 3.81 – 3.59 (m, 1H), 3.28 – 3.15 (m, 2H), 1.10 (s, 9H).

^{13}C-DEPT NMR (126 MHz, DMSO-d_6) δ 137.1, 134.1, 126.2, 125.4, 124.7, 123.5, 122.4, 110.2, 110.0, 75.5, 67.2, 66.1, 60.6, 60.0, 49.1, 30.2, 26.8, 26.6, 26.2, 24.9.

HRMS (ESI-ORBITRAP): m/z $[M+H]^+$ calc. for $C_{33}H_{38}N_7O_{11}$: 708.2624; found: 708.2623.

Ether-C-D-isostere (17)

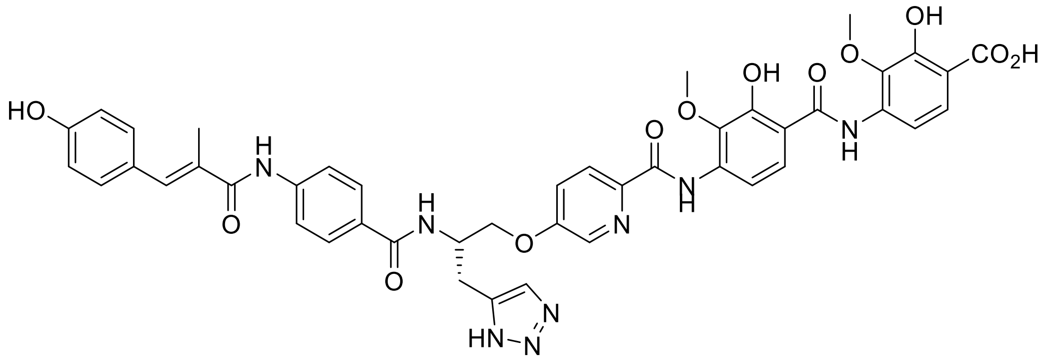

Chemical Formula: $C_{44}H_{40}N_8O_{12}$
Exact Mass: 872.2766

To a solution of tetrapeptide (**17m**, 200 mg, 269 µmol, 1.00 eq.) in DMF (1 mL) was added triethylamine (375 µL, 2.69 mmol, 10.0 eq.) and activated AB building block[1] (147 mg, 269 µmol, 1.00 eq.) and the reaction mixture was stirred under exclusion of light for 2 d at room temperature. After completion of the reaction the reaction mixture was cooled to 0 °C and potassium hydroxide (3 M, 1 mL) was added. The reaction mixture was stirred for 30 min after which an aqueous solution of HCl (3 M, 1 mL) was added. The volatiles were removed by rotary evaporation and the residue was taken up by DMSO (1 mL). The mixture was centrifuged, decanted and the supernatant was subjected to RP-HPLC purification. The product (**17**, 40.3 mg, 40.4 µmol, 15 %) was obtained as white solid.

RP-HPLC: 43-55 % MeCN (+ 0.1% TFA) in milliQ H_2O (+0.1% TFA); 30 min; 70 mL/min.

^{1}H NMR (700 MHz, DMSO-*d*6) δ 11.71 (s, 1H), 11.58 (s, 1H), 11.12 (s, 1H), 10.44 (s, 1H), 10.05 (s, 1H), 9.75 (s, 1H), 8.48 (d, *J* = 2.8 Hz, 1H), 8.44 (d, *J* = 8.1 Hz, 1H), 8.17 (d, *J* = 8.7 Hz, 1H), 8.11 (dd, *J* = 8.9, 3.3 Hz, 1H), 8.05 – 7.99 (m, 1H), 7.87 (d, *J* = 8.8 Hz, 1H), 7.79 (s, 4H), 7.71 (dd, *J* = 8.7, 2.9 Hz, 1H), 7.67 (s, 1H), 7.59 (d, *J* = 8.8 Hz, 1H), 7.34 (d, *J* = 8.3 Hz, 2H), 7.25 (s, 1H), 6.83 (d, *J* = 8.3 Hz, 2H), 4.64 (h, *J* = 6.5 Hz, 1H), 4.34 (dd, *J* = 10.1, 6.2 Hz, 1H), 4.30 (dd, *J* = 10.1, 5.4 Hz, 1H), 3.91 (s, 3H), 3.85 (s, 3H), 3.17 (dd, *J* = 14.8, 5.7 Hz, 1H), 3.10 (dd, *J* = 14.9, 8.4 Hz, 1H), 2.10 (s, 3H).

(^{1}H,^{13}C)-HSQC_ed NMR (176 MHz, DMSO-*d*6) δ 137.7, 124.2, 110.7, 110.9, 127.0, 119.5, 128.4, 122.8, 126.2, 131.8, 134.3, 115.8, 49.0, 70.2, 60.6, 61.3, 15.0, 15.0, 29.4.

HRMS (ESI-ORBITRAP): *m/z* $[M+H]^+$ calc. for $C_{44}H_{41}N_8O_{12}$: 873.2838, found: 873.2839, R_T = 8.17 min.

Guanidino-Albicidin (20)

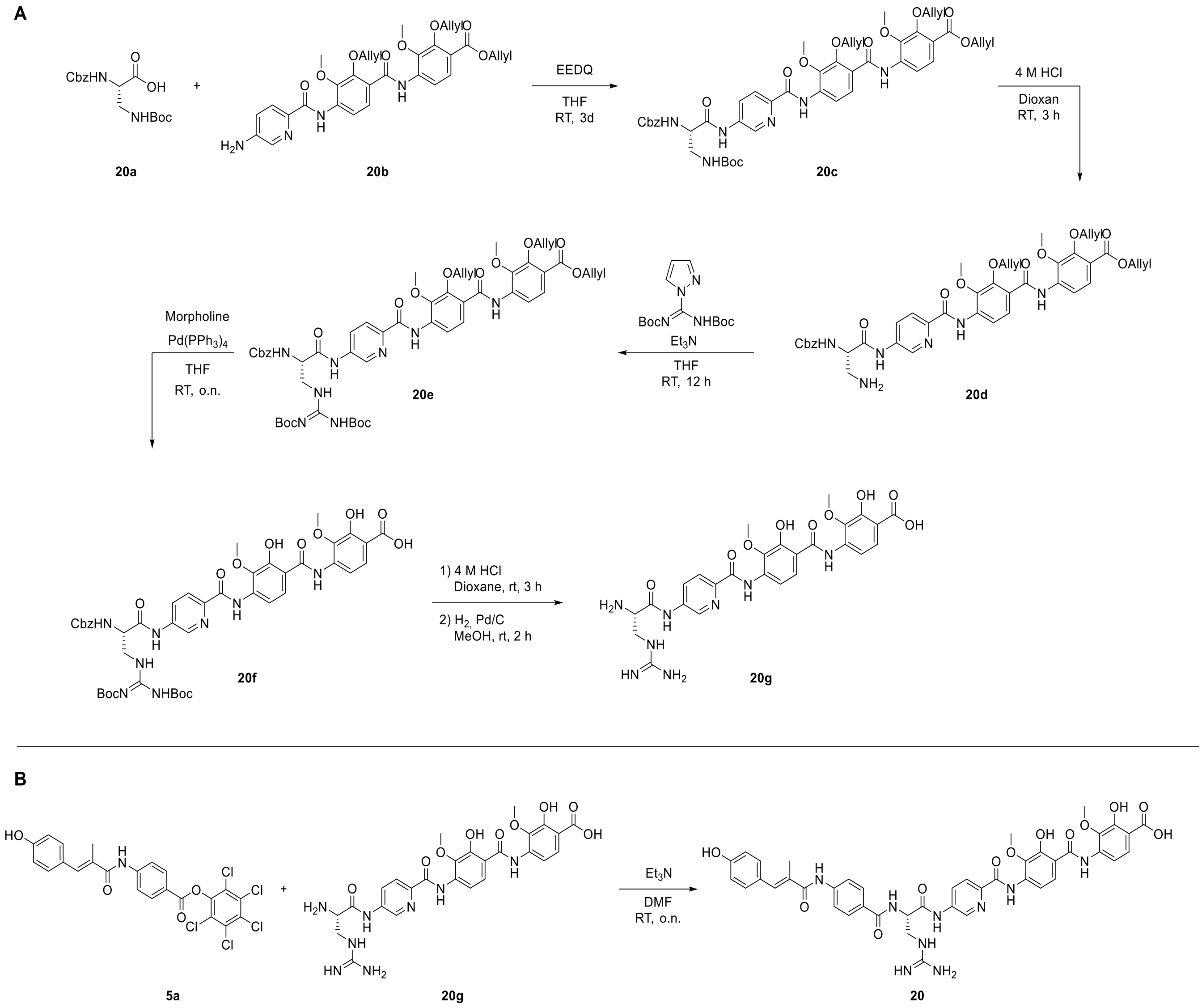

Scheme 6: **A)** Synthesis route of guanidino tetrapeptide **20g**. **B)** Final coupling of tetrapeptide **20g** with activated AB building block **5a** to obtain derivative **20**.

Cbz/Boc/Allyl-protected amino tetrapeptide precursor (20c)

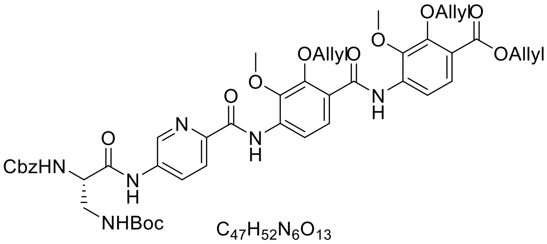

$C_{47}H_{52}N_6O_{13}$
908.3592

To a solution of the amino acid **20a** (100 mg, 296 µmol, 2.00 eq.) in dry THF was added EEDQ (73.1 mg, 296 µmol, 2.00 eq.) and the reaction mixture was stirred at room temperature for 15 min. A premixed solution of the tripeptide **20b**[18] (87.0 mg, 148 µmol, 1.00 eq.) in dry THF was added slowly and the reaction mixture was stirred at room temperature for 72 h. The organic solvent was evaporated under reduced pressure and the residue was partitioned between EtOAc and 1 M $HCl_{(aq.)}$. The organic layer was washed with 1 M $HCl_{(aq.)}$ (2x), brine, dried over anhydr. Na_2SO_4, and concentrated *in vacuo* to afford the crude product, which was purified by column chromatography (SiO_2, Hexane/EtOAc – 8:1).

^{1}H NMR (DMSO-d_6, 500MHz): δ = 1.32 (1H, s), 3.94 (1H, s), 4.01 (1H, s), 4.31 (1H, dd, *J* = 9.84 Hz), 4.55 (1H, d, *J* = 5.70 Hz), 4.78 (1H, d, *J* = 5.50 Hz), 4.85 (1H, d, *J* = 6.25 Hz), 5.08 (1H, t, *J* = 9.90 Hz), 5.28 (1H, m, *J* = 2.58 Hz), 5.41 (1H, m, *J* = 3.76 Hz), 6.09 (1H, m, *J* = 2.90 Hz), 6.89 (1H, d, *J* = 12.00 Hz), 7.25 (1H, d, *J* = 1.10 Hz), 7.34 (1H, dd, *J* = 4.38 Hz), 7.38 (1H, s), 7.51 (1H, d, *J* = 7.25 Hz), 7.58 (1H, d, *J* = 8.75 Hz), 7.88 (1H, d, *J* = 8.85 Hz), 8.21 (1H, s), 8.34 (1H, d, *J* = 8.75 Hz), 8.40 (1H, d, *J* = 8.85 Hz), 8.94 (1H, s), 10.60 (1H, s), 10.64 (1H, s), 10.69 (1H, s)

^{13}C NMR (DMSO-d_6, 126 MHz): δ = 164.9, 162.0, 151.6, 149.8, 143.7, 142.9, 142.2, 134.4, 133.1, 133.1, 128.8, 128.4, 128.3, 127.0, 122.3, 120.8, 120.7, 118.6, 118.3, 115.3, 75.7, 75.0, 66.2, 65.6, 61.6, 61.5, 28.5 ppm.

Cbz/Allyl-protected amino tetrapeptide precursor (20d)

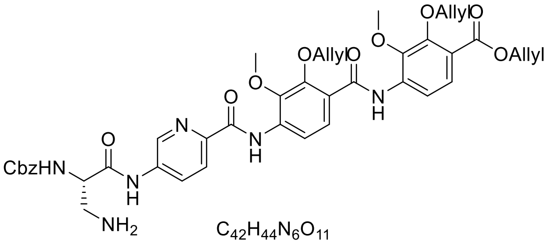

$C_{42}H_{44}N_6O_{11}$
808.3068

A solution of the Boc-protected tetrapeptide **20c** (500 mg, 550 µmol, 1.00 eq.) in 4 M HCl in 1,4-dioxane (10 mL) was stirred at room temperature for 2 h. Subsequently, all volatiles were removed under reduced pressure to obtain the crude product, which was taken up in H_2O/CH_3CN and freeze-dried to afford the Boc-deprotected amine **20d** (465 mg, quant) as yellow/orange powder.

^{1}H NMR (Dioxane-d_8, 500MHz): δ = 4.42 (1H, s), 4.74 (1H, m, *J* = 4.80 Hz), 4.86 (1H, m, *J* = 6.48 Hz), 4.98 (1H, s), 5.04 (1H, s), 5.40 (1H, dd, *J* = 5.92 Hz), 5.59 (1H, d, *J* = 5.60 Hz), 5.82 (1H, d, *J* = 5.35 Hz), 5.89 (1H, d, *J* = 6.05 Hz), 6.11 (1H, dd, *J* = 11.90 Hz), 6.32 (1H, m, *J* = 7.33 Hz), 6.45 (1H, m, *J* = 4.57 Hz), 7.13 (1H, m, *J* = 3.64 Hz), 7.92 (1H, s), 8.26 (1H, d, *J* = 26.41 Hz), 8.42 (1H, d, *J* = 4.10 Hz), 8.62 (1H, d, *J* = 8.75 Hz), 8.89 (1H, s), 8.92 (1H, d, *J* = 8.85 Hz), 9.06 (1H, d, *J* = 6.80 Hz), 9.24 (1H, d, *J* = 8.50 Hz), 9.34 (1H, d, *J* = 8.45 Hz), 9.38 (1H, d, *J* = 8.90 Hz), 9.44 (1H, d, *J* = 8.90 Hz), 9.60 (1H, d, *J* = 5.05 Hz), 9.97 (1H, s), 11.64 (1H, s), 11.68 (1H, s), 11.78 (1H, s), 12.52 (1H, s).

^{13}C NMR (Dioxane-d$_8$, 126MHz): δ = 187.3, 162.8, 156.3, 142.9, 137.0, 133.1, 128.8, 128.3, 120.8, 120.7, 118.6, 118.3, 103.8, 78.7, 75.0, 69.6, 66.3, 65.6, 61.6, 61.5, 28.3, 28.2, 28.0 ppm.

Cbz/Boc/allyl-protected guanidino tetrapeptide (20e)

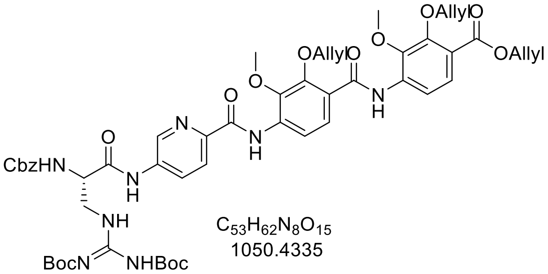

The amine **20d** (465 mg, 574 µmol, 1.00 eq.) and 1-[*N,N'*-(Di-Boc)amidino]pyrazole (187 mg, 604 µmol, 1.05 eq.) were dissolved in anhydrous THF and Et_3N (240 µL, 3.00 eq.) was added. The reaction mixture was stirred at room temperature for 12 h. All volatiles were removed *in vacuo,* the residue was taken up in ethyl acetate and washed with 1 M $HCl_{(aq.)}$ (2x), water (2x) and brine. The organic phase was dried over anhydrous Na_2SO_4 and concentrated *in vacuo* to obtain the crude product **20e** (600 mg, quant). The product was used in the next reaction without further purification.

HRMS (ESI): m/z calculated for $C_{53}H_{62}N_8O_{15}$ $(M+H)^+$ 1051.4407, found 1051.4414.

Unprotected guanidino tetrapeptide (20f)

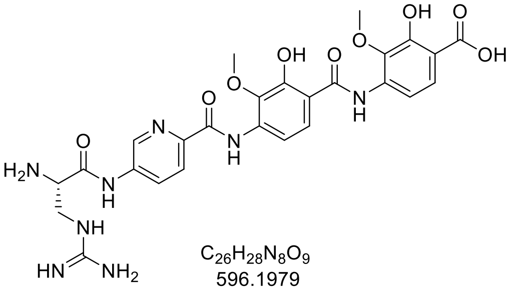

The tetrapeptide **20e** (150 mg, 205 µmol, 1.00 eq.) was dissolved in 4 M HCl in 1,4-dioxane and stirred at room temperature for 3 h. Subsequently, all volatiles were removed under reduced pressure to obtain the crude product, which was taken up in H_2O/CH_3CN and freeze-dried to afford the Boc-deprotected product as yellow powder. HRMS (ESI): m/z calculated for $C_{34}H_{35}N_8O_{11}$ $(M+H)^+$ 731.2420, found 731.2417. Boc deprotected tetrapeptide (115 mg, 124 µmol, 1.00 eq.) was dissolved in dry THF (5 mL) and the solution was purged with argon for 10 min. Palladium (10 wt. % on activated carbon, 17 mg) was added, the reaction mixture was saturated with H_2 and stirred at room temperature for 5 h under hydrogen atmosphere. The suspension was filtered over Celite®, concentrated under reduced pressure, and freeze-dried to afford the Cbz-Boc-deprotected tetrapeptide **20f** (90 mg, quant over two steps) as yellow powder.

Cbz/Boc-protected guanidino tetrapeptide (20g)

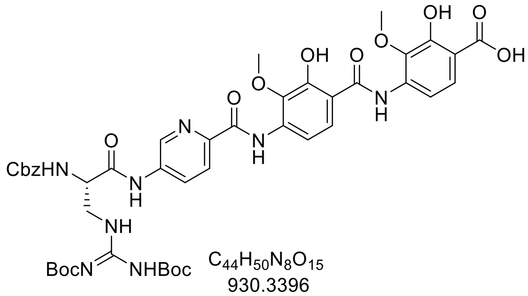

The allyl-protected tetrapeptide **20f** (500 mg, 476 µmol, 1.00 eq.) was dissolved in dry THF and morpholine (0.821 mL, 20.0 eq.) was added, followed by either $Pd(PPh_3)_4$ (220 mg, 190 µmol, 0.400 eq.) The reaction mixture was stirred at room temperature for 16 h under the exclusion of light (aluminum wrap). After removing all volatiles under reduced pressure, the residue was taken up in EtOAc and washed with 1 M $HCl_{(aq.)}$ (3x) and brine. The organic phase was dried over anhydrous Na_2SO_4 and concentrated *in vacuo* to obtain the crude product, which was purified by column chromatography (SiO_2, 5-10 % MeOH in DCM). The allyl-deprotected tetrapeptide **20g** (162 mg, 37 %) was obtained as an orange solid.

^{1}H NMR (DMSO-d$_6$, 500MHz): δ = 1H-NMR (DMSO) 1.31 (1H, s), 1.47 (1H, s), 1.92 (1H, s), 3.51 (1H, s), 3.68 (1H, t, *J* = 6.98 Hz), 3.82 (1H, t, *J* = 6.49 Hz), 3.88 (1H, s), 3.89 (1H, s), 4.35 (1H, dd, *J* = 5.98 Hz), 5.07 (1H, dd, *J* = 11.68 Hz), 7.22 (1H, t, *J* = 13.96 Hz), 7.33 (1H, m, *J* = 4.16 Hz), 7.38 (1H, d, *J* = 4.31 Hz), 7.51 (1H, d, *J* = 8.66 Hz), 7.77 (1H, d, *J* = 8.63 Hz), 7.88 (1H, d, *J* = 8.92 Hz), 8.02 (1H, d, *J* = 6.88 Hz), 8.10 (1H, d, *J* = 8.96 Hz), 8.20 (1H, d, *J* = 8.50 Hz), 8.29 (1H, dd, *J* = 3.48 Hz), 8.56 (1H, s), 8.93 (1H, d, *J* = 1.70 Hz), 10.50 (1H, s), 10.73 (1H, s), 10.92 (1H, s), 11.48 (1H, s), 11.78 (1H, s) ppm.

HRMS (ESI): m/z calculated for $C_{44}H_{55}N_8O_{15}$ $(M+H)^+$ 931.3468, found 931.3471.

Guanidino-Albicidin (20)

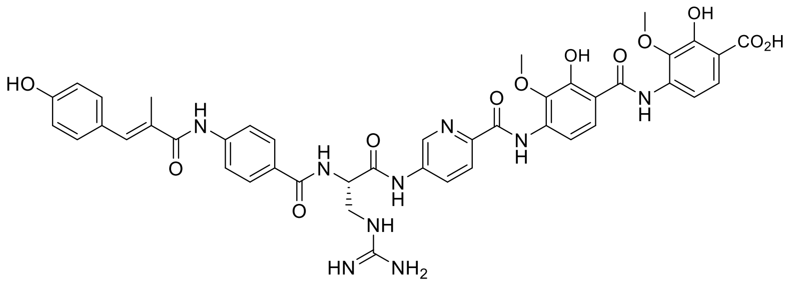

Guanidino-Albi
$C_{43}H_{41}N_9O_{12}$
875.2875

The tetrapeptide **20g** (135 mg, 226 µmol, 1.00 eq.) and PCP active ester (124 mg, 226 µmol, 1.00 eq.) were dissolved in a mixture of anhydrous DMF and Et_3N (47.3 µl, 340 µmol). The reaction mixture was stirred at room temperature for 16 h under the exclusion of light. All volatiles were removed *in vacuo* and the crude material was dissolved in DMSO and purified by HPLC (PLRP-S column, CH_3CN in H_2O). The final derivative **20** (14 mg, 7 %) was obtained as a colorless solid.

^{1}H NMR (DMSO-d$_6$, 500MHz): δ = 2.12 (10H, s), 3.66 (17H, d, *J* = 61.42 Hz), 3.90 (22H, d, *J* = 22.51 Hz), 4.88 (2H, d, *J* = 6.65 Hz), 6.85 (8H, d, *J* = 8.15 Hz), 7.28 (4H, s), 7.36 (9H, d, *J* = 8.05 Hz), 7.59 (4H, d, *J* = 8.75 Hz), 7.69 (2H, s), 7.87 (12H, dd, *J* = 7.60 Hz), 7.94 (7H, d, J = 8.35 Hz), 8.02 (4H, d, *J* = 8.95 Hz), 8.11 (4H, d, J = 8.85 Hz), 8.23 (4H, d, *J* = 8.05 Hz), 8.37 (3H, d, *J* = 8.15 Hz), 8.84 (3H, d, *J* = 7.25 Hz), 9.01 (3H, s), 9.80 (1H, t, *J* = 1.28 Hz), 10.11 (3H, s), 10.49 (3H, s), 10.97 (2H, s), 11.12 (3H, s), 11.75 (2H, s)

^{13}C NMR (DMSO-d$_6$, 126 MHz): δ = 140.1, 134.5, 131.8, 128.8, 128.0, 127.0, 127.0, 126.1, 126.1, 123.4, 119.6, 115.8, 115.8, 110.9, 110.9, 110.7, 110.7, 61.3, 60.8, 53.9, 14.9 ppm.

HRMS (ESI): m/z calculated for $C_{43}H_{42}N_9O_{12}$ $(M+H)^+$ 876.2947, found 876.2950.

Sulfonamide-E-F-isostere (21) Synthesis conducted as previously reported.[18]

L-*trans*-hydroxyproline-albicidin (22)

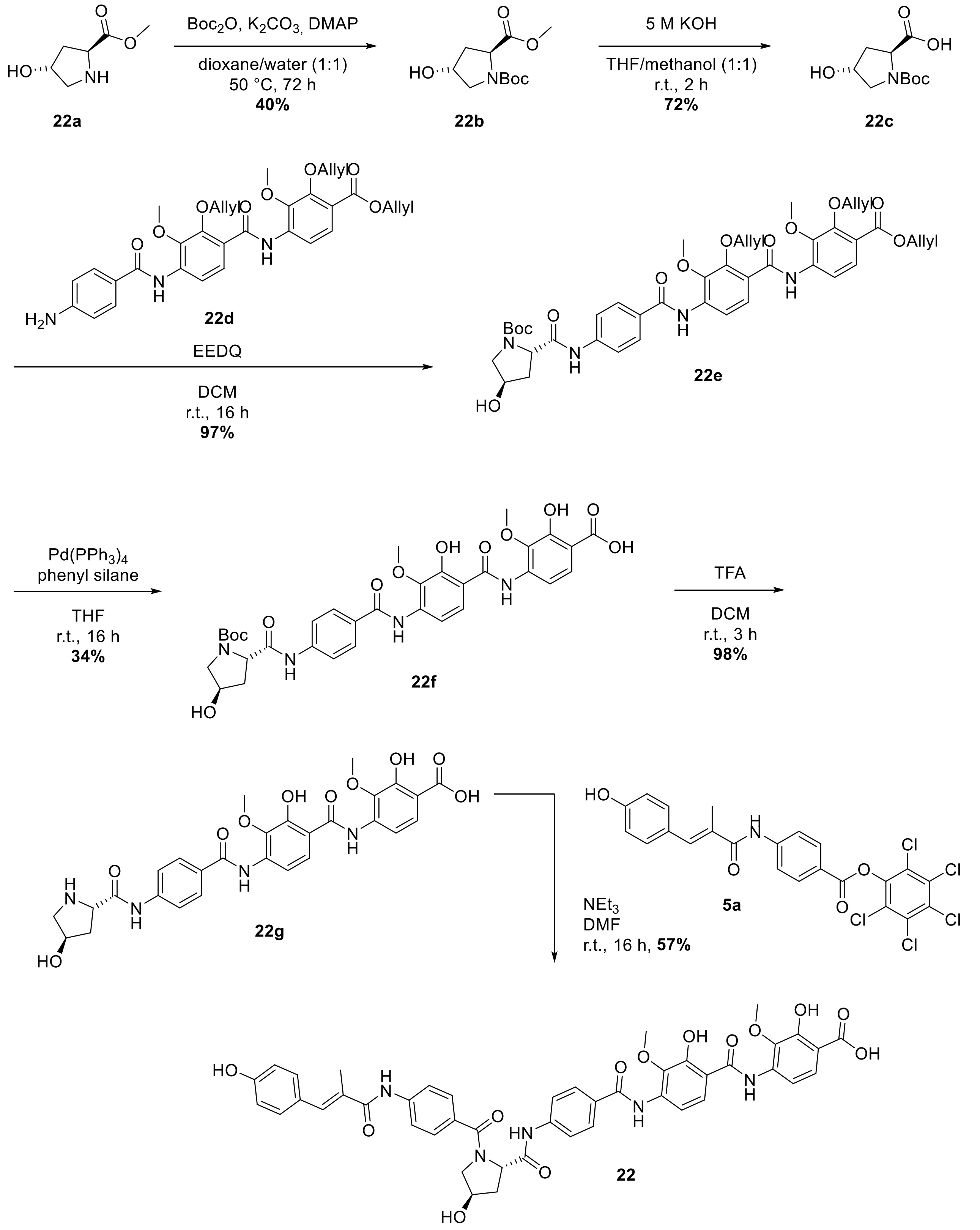

Scheme 7: Synthesis route of L-*trans*-hydroxyproline-albicidin **22** starting from hydroxy-proline methyl ester **22a.**

1-(*tert*-Butyl)-2-methyl (2*S*,4*R*)-4-hydroxypyrrolidin-1,2-dicarboxylat (22b)

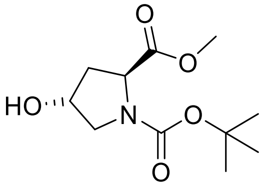

To a solution of methyl-(2*S*,4*R*)-4-hydroxy-pyrrolidin-2-carboxylate (**22a**, 2.22 g, 15.3 mmol, 1.00 eq) in a mixture of 1,4-dioxane/water (100 mL, 1:1) was added potassium carbonate (4.30 g, 31.1 mmol, 2.03 eq), di-*tert*-butyldicarbonate (3.67 g, 16.8 mmol, 1.10 eq) and 4-(dimethylamino)-pyridine (93.4 mg, 0.770 mmol, 0.050 eq). The reaction mixture was stirred for 72 h at 50 °C. The volatiles were removed by rotary evaporation and the residue was acidified by 1 M $HCl_{(aq.)}$ to pH 2. The aqueous phase was extracted by ethyl acetate (3 x 30 mL). The combined organic phases were washed by distilled water (30 mL) und a saturated solution of sodium chloride (30 mL), dried over sodium sulphate and filtered. After evaporation of the solvent, product (**22b**, 1.51 g, 6.16 mmol, 40 %) was obtained as colorless oil.

^{1}H NMR (DMSO-d$_6$, 400 MHz, mixture of rotamers): δ (ppm) = 5.10 (d, *J* = 3.5 Hz, 1 H), 4.16 - 4.21 (m, 1 H), 3.65 (s, 3 H), 3.57 (s, 1 H), 3.37 - 3.43 (m, 1 H), 3.23 - 3.30 (m, 1 H), 2.11 (d, *J* = 9.5 Hz, 1 H), 1.82 - 1.93 (m, 1 H), 1.30 - 1.41 (m, 9 H).

^{13}C NMR (DMSO-d$_6$, 101 MHz, mixture of rotamers): δ (ppm) = 173.3, 172.9, 153.8, 153.0, 79.1, 79.0, 68.5, 67.8, 66.4, 57.7, 57.4, 54.7, 54.5, 51.8, 51.8, 37.9, 28.1, 27.9.

HRMS (ESI): *m/z* calc. for $C_{11}H_{19}NO_5$ $[M+Na]^+$: 268.1156; found: 246.1149.

(2*S*,4*R*)-1-(*tert*-Butoxycarbonyl)-4-hydroxypyrrolidine-2-carbonic acid (22c)

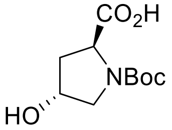

A solution of 1-(*tert*-butyl)-2-methyl-(2*S*,4*R*)-4-hydroxypyrrolidine-1,2-dicarboxylate (**22b**, 315 mg, 1.28 mmol, 1.00 eq) in a mixture of THF/methanol (6 mL, 1:1) was cooled to 0 °C. To this solution was added an aqueous solution of KOH (5 M, 3 mL) and the reaction mixture was stirred for 2 h at room temperature. After completion of the reaction the solvent was removed by rotary evaporation, the residue was diluted by water, acidified with 1 M $HCl_{(aq.)}$ to pH 1 and extracted with ethyl acetate (3 × 15 mL). The combined organic phases were washed by a saturated solution of sodium chloride (30 mL), dried over sodium sulphate and filtered. The solvent was removed by rotary evaporation and the product (**22c**, 213.8 mg, 0.92 mmol, 72 %) was isolated as a white powder.

^{1}H NMR (DMSO-d$_6$, 400 MHz, mixture of rotamers): δ (ppm) = 12.51 (br. s, 1 H), 5.06 (br. s, 1 H), 4.24 (br. s, 1 H), 4.09 - 4.13 (m, 1 H), 3.38 - 3.41 (m, 1 H), 3.23 - 3.26 (m, 1 H), 2.06 - 2.15 (m, 1 H), 1.84 - 1.91 (m, 1 H), 1.34, 1.39 (2s, 9 H). **^{13}C NMR (DMSO-d$_6$, 101 MHz):** δ (ppm) = 174.4, 153.8, 78.8, 68.5, 67.8, 59.8, 57.7, 28.1, 27.9.

HRMS (ESI): *m/z* calc. for $C_{10}H_{17}NO_5$ $[M-H]^-$: 230.1034; found: 230.1031.

Boc-Hyp-pABA-HMpABA(Allyl)-HMpABA(Allyl)-OAllyl (22e)

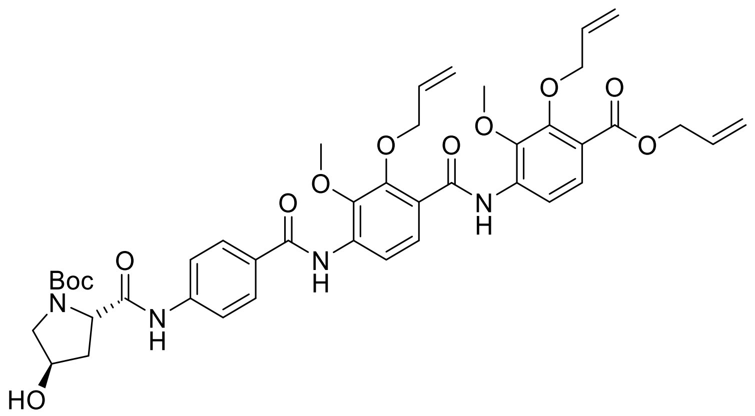

Carboxylic acid (**22b**, 218 mg, 0.940 mmol, 4.81 eq) was dissolved in absolute DCM (20 mL), cooled to −15 °C and *N*-ethoxycarbonyl-2-ethoxy-1,2-dihydrochinoline (235 mg, 0.950 mmol, 4.85 eq) was added. The reaction mixture was stirred for 15 min after which the allyl-protected tripeptide[21] (**22d**, 115 mg, 0.200 mmol, 1.00 eq) was added. The resulting mixture was stirred for 16 h at room temperature and after completion of the reaction the solvent was removed by rotary evaporation. The residue was taken up by ethyl acetate (20 mL), washed by a saturated solution of sodium bicarbonate (30 mL), a solution of sodium chloride (30 mL), dried over sodium sulphate and filtered. The solvent was removed by rotary evaporation and the crude product was purified by column chromatography (SiO_2, 1 % → 10 % MeOH in DCM). The product (**22e** ,152 mg, 0.190 mmol, 97 %) was obtained as a yellow oil.

^{1}H NMR ($CDCl_3$, 400 MHz): δ (ppm) = 10.65 (s, 1 H), 9.92 (s, 1 H), 8.63 (s, 1 H), 8.32 - 8.43 (m, 2 H), 7.92 - 7.99 (m, 1 H), 7.72 - 7.79 (m, 2 H), 7.64 (d, *J* = 8.5 Hz, 2 H), 6.08 - 6.20 (m, 2 H), 5.99 – 6.08 (m, 1 H), 5.33 - 5.44 (m, 3 H), 5.22 - 5.31 (m, 3 H), 4.79 (d, 2 H), 4.76 (d, 2 H), 4.64 (m, 1 H), 4.56 (d, 2 H), 4.55 (m, 1 H), 4.02 (s, 3 H), 3.98 (s, 3 H), 3.57 - 3.63 (m, 1 H), 3.49 - 3.57 (m, 1 H), 3.48 - 3.57 (m, 1 H), 3.26 - 3.44 (m, 1 H), 2.49 - 2.60 (m, 1 H), 2.06 – 2.15 (m, 1 H), 1.42 - 1.55 (m, 9 H).

^{13}C NMR ($CDCl_3$, 101 MHz): δ (ppm) = 165.2, 164.8, 163.0, 151.8, 149.3, 142.8, 141.5, 137.1, 136.5, 134.0, 132.4, 128.2, 127.5, 127.4, 122.1, 120.7, 120.3, 119.5, 118.5, 118.2, 115.5, 115.4, 81.5, 75.8, 75.2, 65.7, 61.3, 61.2, 50.8, 28.5.

HRMS (ESI): *m/z* calc. for $C_{42}H_{48}N_4O_{12}$ $[M+H]^+$: 801.3341; found: 801.3332.

Boc-Hyp-pABA-HMpABA-HMpABA-OH (22f)

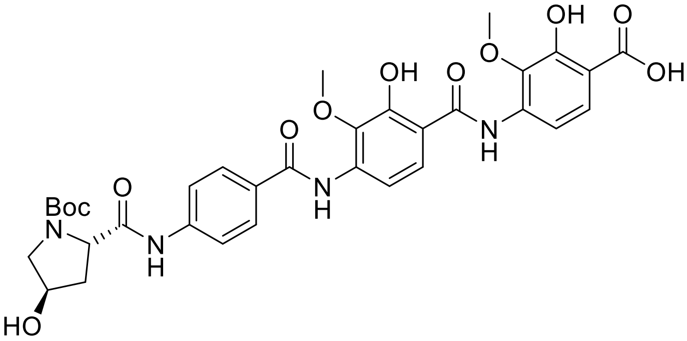

The allyl-protected tetrapeptide (**22e**, 148 mg, 0.15 mmol, 1.00 eq) was dissolved in absolute THF (8 mL) and phenyl silane (146 µL, 1.18 mmol, 8.02 eq) was added. After addition of $Pd(PPh_3)_4$ (17.6 mg, 0.015 mmol, 0.100 eq) the reaction mixture was stirred for 16 h at room temperature. The solvent was removed by rotary evaporation and the crude product was purified by column chromatography (SiO_2, 1 % → 40 % MeOH in DCM). The product (**22f**, 44.0 mg, 0.065 mmol, 34 %) was obtained as brownish solid.

^{1}H NMR (DMSO-d$_6$, 400 MHz): δ (ppm) = 11.62 (br. s, 1 H), 10.90 (s, 1 H), 10.50 (s, 1 H), 9.63 (s, 1 H), 7.96 (d, *J* = 8.8 Hz, 2 H), 7.78 - 7.81 (m, 3 H), 7.65 (d, *J* = 8.5 Hz, 1 H), 7.54 (d, *J* = 9.0 Hz, 1 H), 7.46 (d, *J* = 8.5 Hz, 1 H), 5.16 (br. s., 1 H), 4.37 - 4.45 (m, 1 H), 4.31 (br. s., 1 H), 3.86 (s, 3 H), 3.79 (s, 3 H), 2.14 – 2.19 (m, 1 H), 1.92 – 1.98 (m, 1 H), 1.11 - 1.38 (m, 9 H).

HRMS (ESI): *m/z* calc. for $C_{33}H_{36}N_4O_{12}$ $[M-H]^-$: 679.2257; found: 679.2250.

Due to the low amount of product no ^{13}C-NMR spectrum was recorded.

HCl*H-Hyp-pABA-HMpABA-HMpABA-OH (22g)

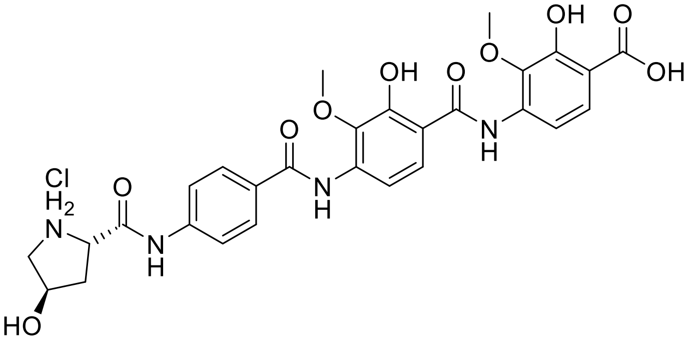

The protected amine (**22f**, 35.0 mg, 0.051 mmol, 1.00 eq) was dissolved in absolute DCM (5 mL), trifluoroacetic acid (2 mL) was added and the reaction mixture was stirred for 3 h at room temperature. The volatiles were removed by rotary evaporation and the residue was taken up with acetonitrile and an aqueous solution of HCl (1 M). The product (**22g**, 31.2 mg, 0.051 mmol, 98 %) was obtained after freeze drying as yellowish solid and used in the next step without further purification.

HRMS (ESI): *m/z* calc. for $C_{28}H_{28}N_4O_{10}$ $[M+H]^+$: 581.1878; found: 581.1888.

L-Hyp-Albicidin (22)

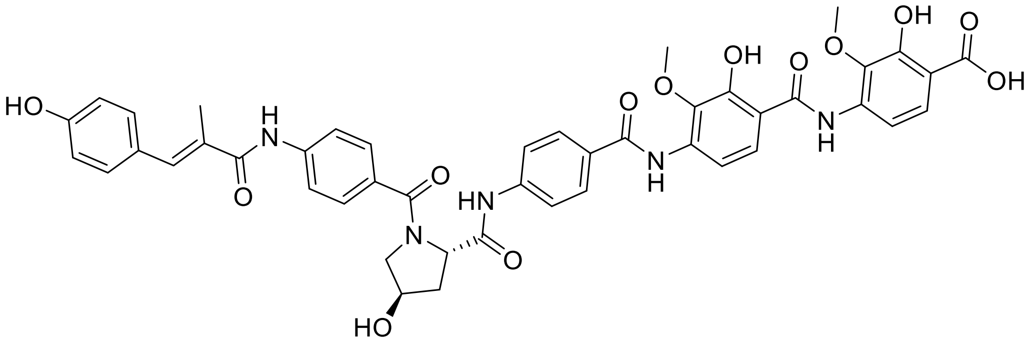

To a solution of tetrapeptide (**22g**, 31.2 mg, 0.051 mmol, 1.00 eq) in absolute DMF (2 mL) was added triethylamine and activated AB building block[1] (**5a**, 30.7 mg, 0.056 mmol, 1.11 eq) and the reaction mixture was stirred for 16 h at room temperature. The resulting mixture was diluted with acetonitrile and filtered. The solid was dissolved in a mixture of THF/H_2O/acetonitrile (3:1:3) and concentrated under reduced pressure. After freeze-drying and preparative RP-HPLC the final product (**22**, 25.0 mg, 0.029 mmol, 57 %) was obtained.

^{1}H NMR (DMSO-d$_6$, 400 MHz): δ (ppm) = 10.46 (s, 1 H), 10.09 (m, 1 H), 9.86 (m, 1 H), 9.10 (br. s, 1 H), 7.95 (s, 1 H), 7.91 (d, *J* = 8.8 Hz, 2 H), 7.76 - 7.85 (m, 3 H), 7.64 - 7.75 (m, 1 H), 7.59 (d, *J* = 8.8 Hz, 2 H), 7.50 - 7.56 (m, 1 H), 7.39 - 7.44 (m, 1 H), 7.35 (d, *J* = 7.5 Hz, 2 H), 7.26 (s., 1 H), 7.00 - 7.17 (m, 1 H), 6.85 (br. s., 2 H), 5.08 - 5.17 (m, 1 H), 4.71 - 4.80 (m, 1 H), 4.31 - 4.39 (m, 1 H), 3.86 - 3.94 (m, 1 H), 3.38 - 3.45 (m, 1 H), 2.89 (s, 3 H), 2.73 (s, 3 H), 2.15 - 2.35 (m, 2 H), 2.11 (s, 3 H), 1.95 - 2.07 (m, 2 H).

HRMS (ESI): *m/z* calc. for $C_{45}H_{41}N_5O_{13}$ $[M+H]^+$: 860.2774;found: 860.2773.
Due to the low product amount no ^{13}C-NMR spectrum was recorded.

2. Supplementary figures

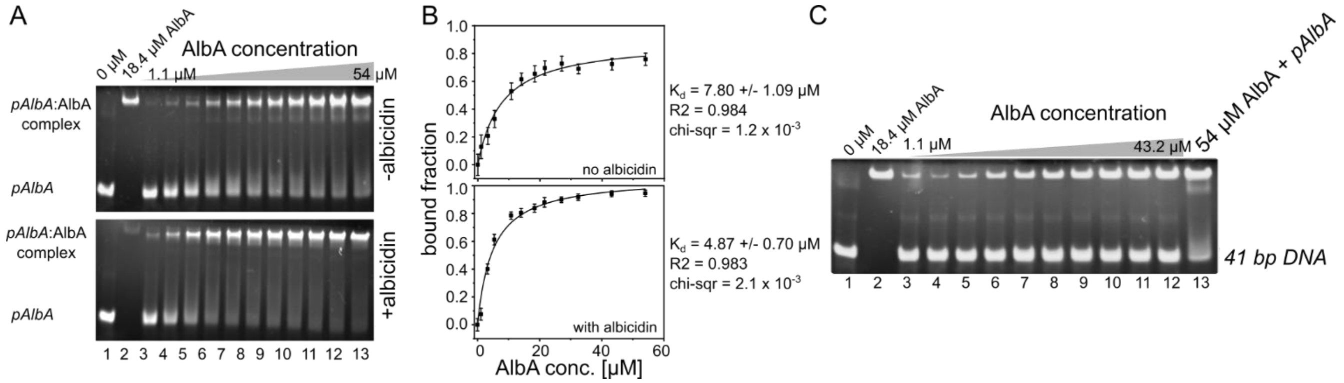

Figure S1. Binding affinity of AlbA to the promoter pAlbA. A) EMSA in absence (top) and presence of albicidin (bottom). Lane 1: *pAlbA* with 10 µM AlbAS (short AlbA without DBD); lane 2: 18.4 µM AlbA without *pAlbA*; lanes 3-13 *pAlbA* with increasing concentrations of AlbA. B) EMSA bands of the free DNA fitted with Hill function (n = 1) and fitting parameters. Error bars represent the signal to noise in gel band quantifications. C) EMSA with AlbA and control 41-bp DNA fragment. Lane 1: control DNA with 10 µM AlbAS (short AlbA without DBD); lane 2: 18.4 µM AlbA without the control DNA, lanes 3-13 control DNA with increasing concentrations of AlbA.

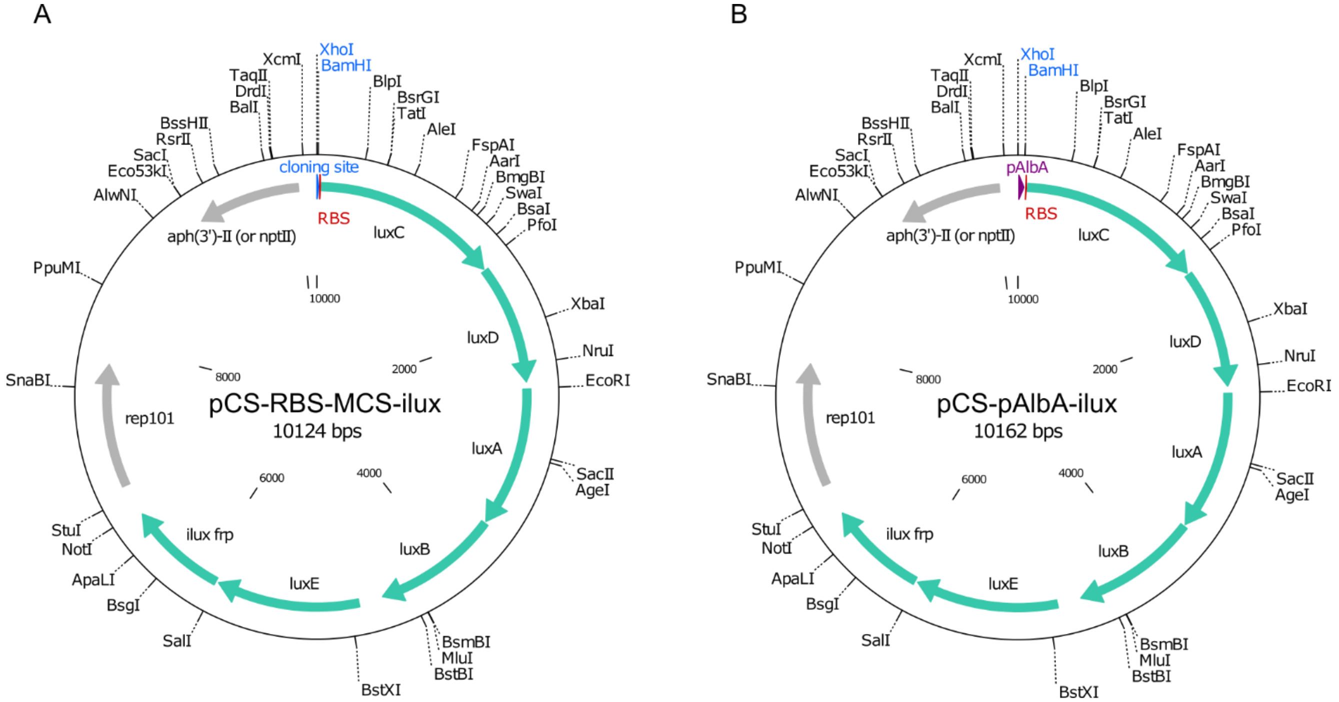

Figure S2. Vector maps of reporter vectors. (A) pCS-RBS-MCS-ilux and (B) pCS-pAlbA-ilux. The genes that are part of the ilux cassette are highlighted in cyan, the ribosome binding site (RBS) and restriction cloning site are shown in red and blue, respectively, and the pAlbA promoter region is coloured in purple.

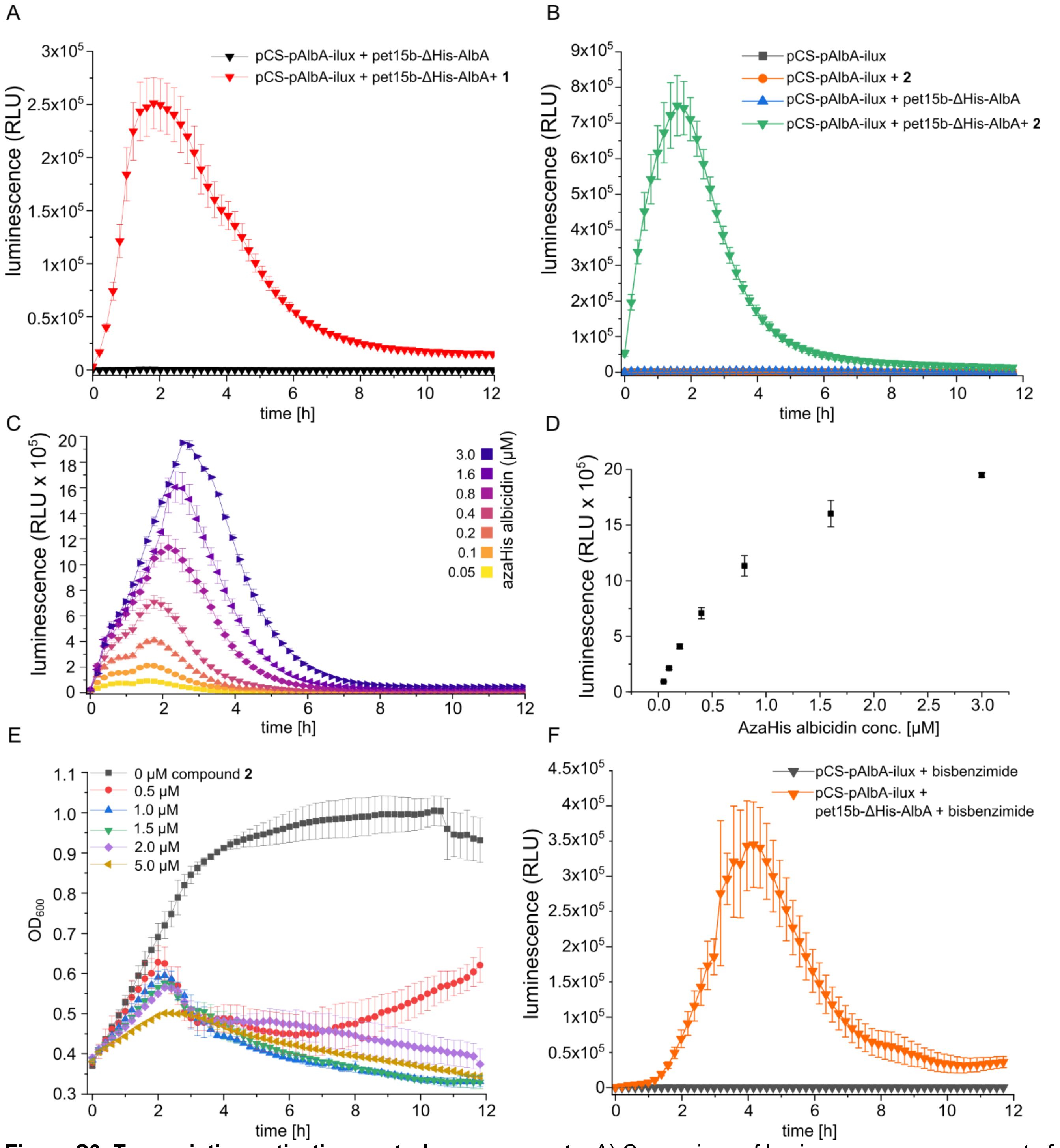

Figure S3. Transcription activation control measurements. A) Comparison of luminescence measurement of *E. coli* BL21 cells transformed with the reporter vector pCS-pAlbA-ilux and pET15b-ΔHisAlbA with (red triangles) and without (black triangles) 0.25 mM albicidin (**1**). B) *E. coli* cells transformed with either only the reporter vector pCS-pAlbA-ilux or the reporter vector and pET15b-ΔHisAlbA in absence and presence of 1.5 µM aza-His albicidin (**2**). Only when both vectors and **2** are present, luminescence is detected. C) Time curves of luminescence intensities in reporter assay after addition of 0, 0.05, 0.1, 0.2, 0.4, 0.8, 1.6 and 3.0 µM aza-His albicidin (**2**). D) Maxima of the luminescence time curves shown in C. E) OD_{600} measurements in presence of 0, 0.5, 1.0, 1.5, 2.0 and 5.0 µM of **2**, acquired in parallel to light emission measurements shown in Figure 2E. F) Control measurements to bisbenzimide transcription induction. *E. coli* cells transformed with either only the reporter vector pCS-pAlbA-ilux or the reporter vector and pET15b-ΔHisAlbA in absence and presence of 5 µM bisbenzimide. E) All measurements were conducted in triplicate and error bars represent the standard deviation of each data point.

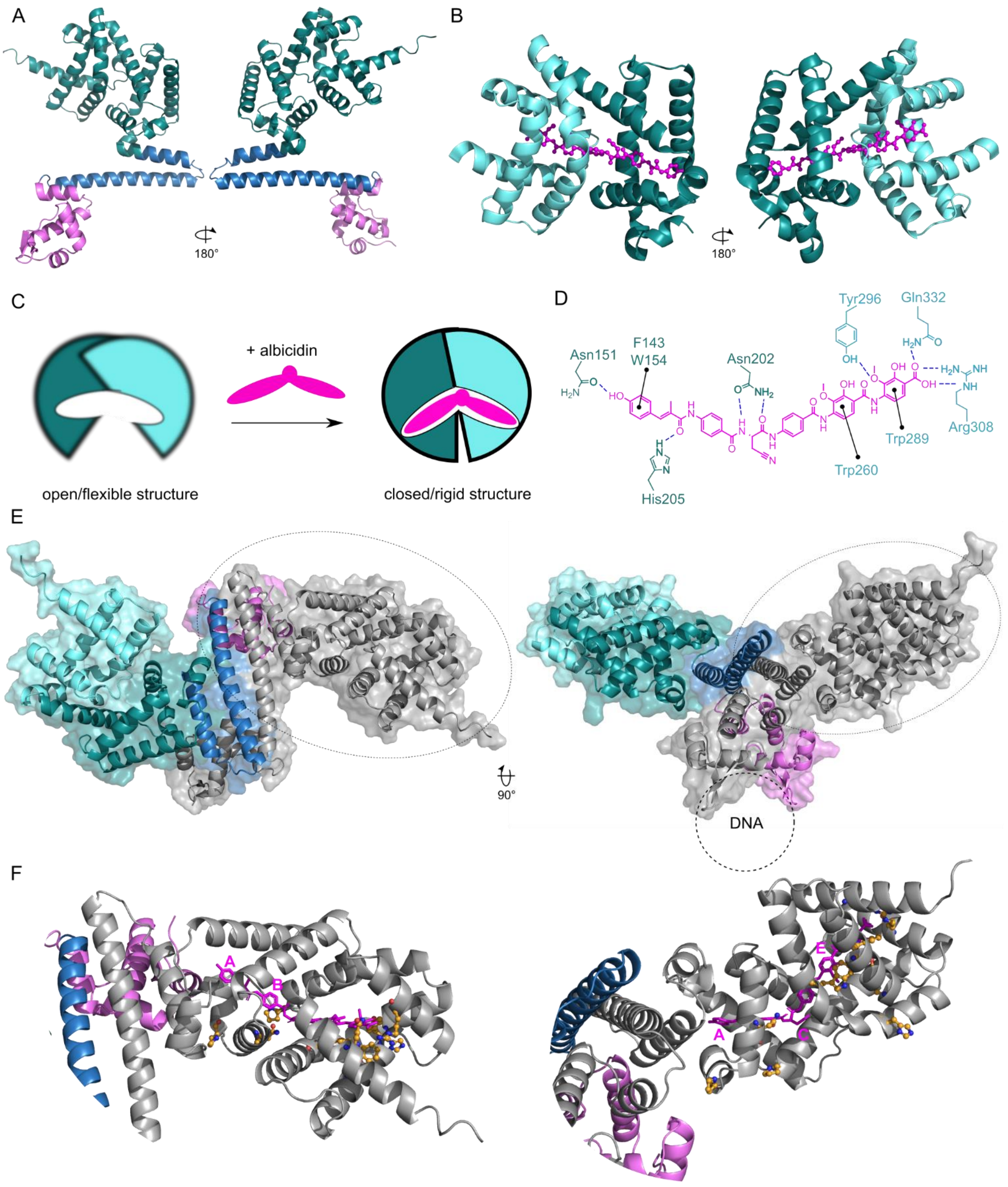

Figure S4. Structure of AlbA and AlbAS. A) Alphafold model of full AlbA. The DNA-binding domain is shown in violet, the coiled-coil dimerization domain is coloured blue and the ligand binding domain is teal. RMSD of the ligand binding domain in the model and the crystal structure of AlbAS is 0.93 Å (193 atoms). B) Crystal structure (PDB-ID: 6ET8) with the pseudodimeric fold of the ligand binding domain of a repeat TipAS fold. The N-terminal

half is shown in dark teal (NTD) and the C-terminal sub-domain (CTD) is shown in light cyan. C) Conformational change from an open, flexible structure to a more rigid, closed structure in the binding mechanism of AlbA suggested by Rostock et al[6]. D) Hydrogen bonds and π-π interactions of albicidin with the subdomains of AlbA. Amino acid residues of the NTD are shown in cyan and residues of the CTD in light cyan. E) Alphafold model of AlbA dimers with one monomer in grey and one monomer depicted in the same colour scheme as in (A). The location of bound DNA (according to structures of other MerR family members) is indicated as a dashed black circle. F) Model of albicidin (pink) in the dimer obtained by alignment of the crystal structure 6ET8. Albicidin building blocks are indicated by magenta labels and mutated residues are shown in yellow.

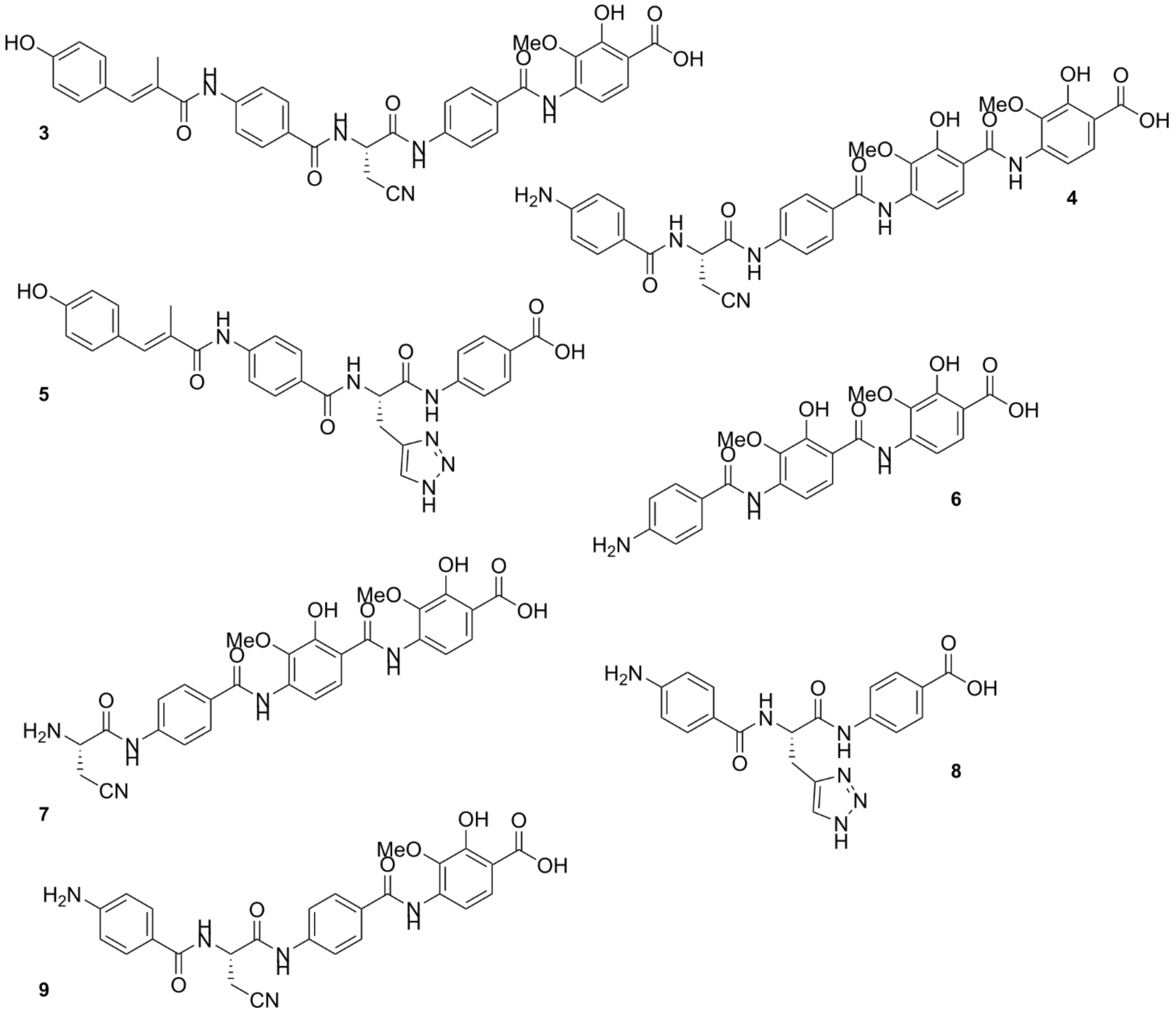

Figure S5. Chemical structures of albicidin fragments.

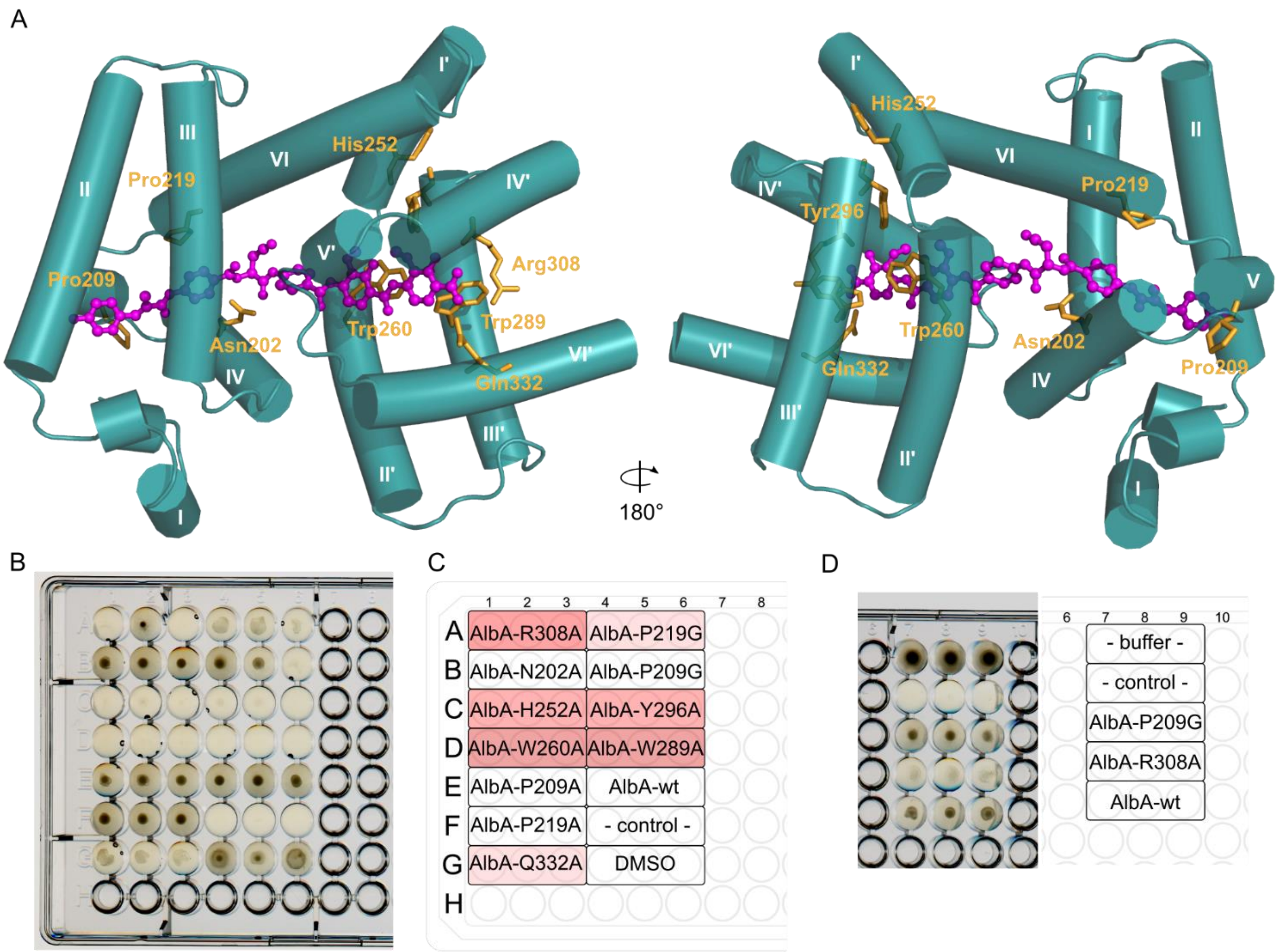

Figure S6. Mutated residues in the ligand binding domain of AlbA. A) Mutated residues in the ligand binding domain of AlbA are shown in gold as sticks with amino acid labels corresponding to their position in the full di-domain protein. The α-helices are numbered sequentially with roman numerals (helices I-VI in the NTD and helices I'-VI' in the CTD). Albicidin is shown in magenta. B) Microdilution tray with liquid cultures of *E. coli* DSM 1116 after incubation with **2** and different purified AlbA mutants and the C) sample loading scheme. The assay was performed in triplicates. AlbA variants with mutations that led to less protection from albicidin are shown in shades of red (darker colours indicate less protection from albicidin). The control contains **2** together with buffer. D) Repeat of growth inhibition assays with mutants AlbA-P209G and AlbA-R308A.

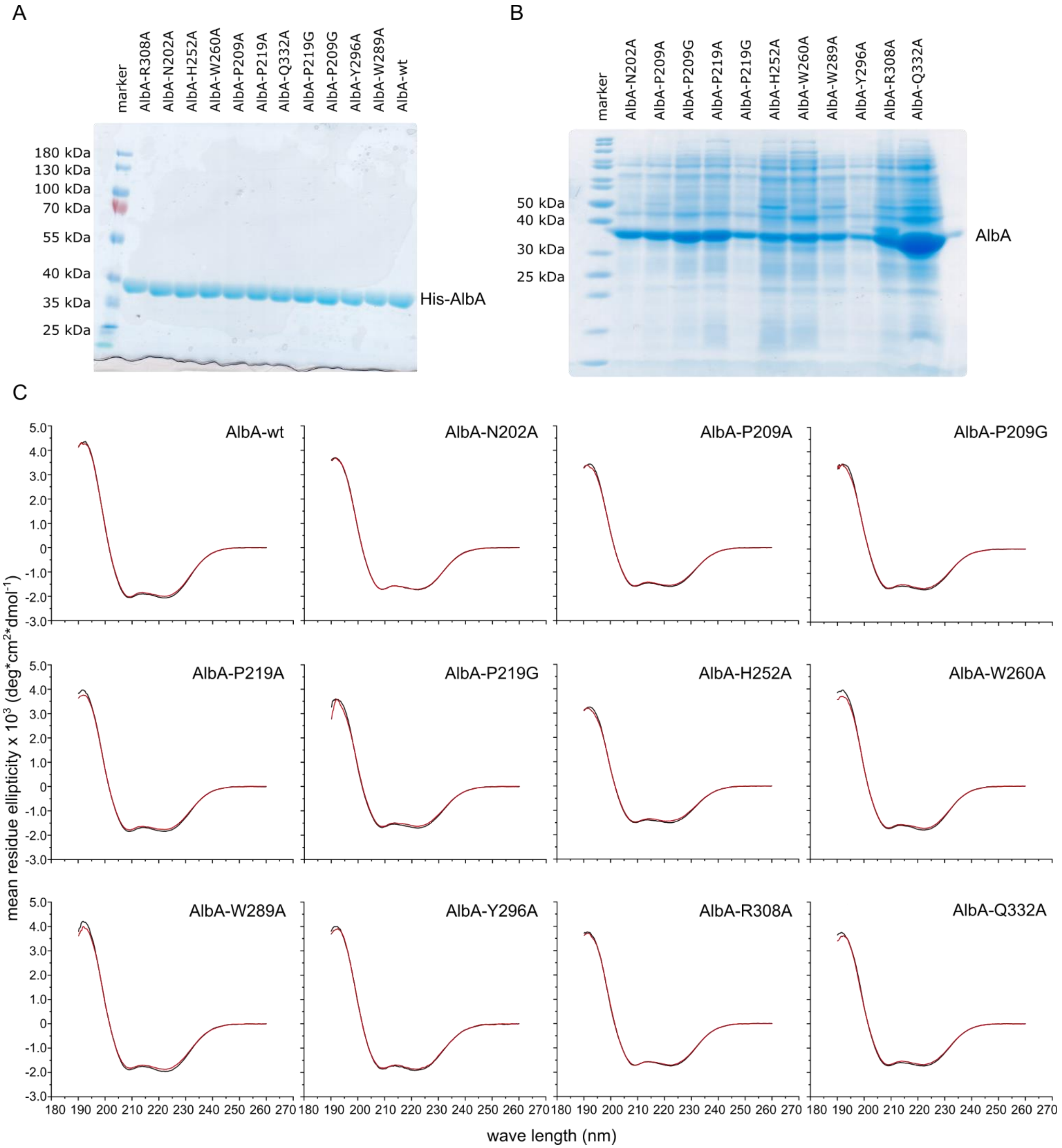

Figure S7. Expression and purification of AlbA mutants. A) SDS-PAGE of Ni-affinity purified His-tagged proteins. B) Soluble fractions of cell lysates from *E. coli* BL21 (DE3) cells with mutant proteins expressed from pet15b without induction with IPTG as used in reporter assays. C) CD spectra of AlbA mutants at 20°C (black line) and 30°C (red line).

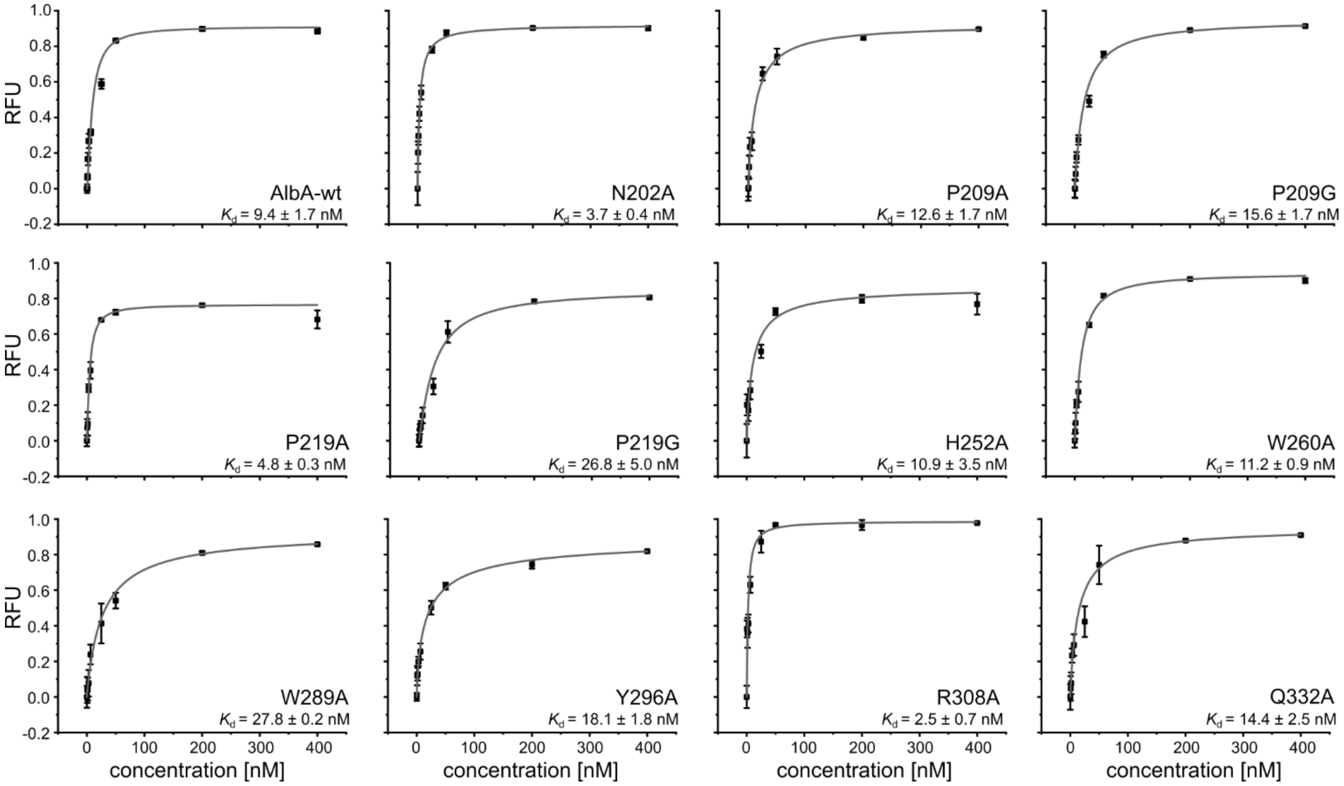

Figure S8. Tryptophan fluorescence quenching of AlbA mutant proteins with aza-His albicidin. Shown are the normalized loss in fluorescence (black squares) and Hill fits (black line) of tryptophane fluorescence quenching titration experiments of AlbA and its mutants with compound **2**. The resulting dissociation constants are listed in Table S2. RFU = relative fluorescence units. Error bars show the standard deviation, n = 3.

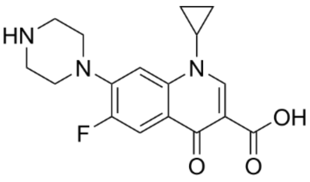

ciprofloxacin

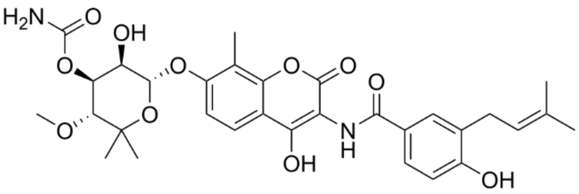

novobiocin

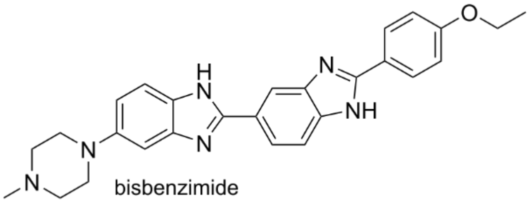

bisbenzimide
(Hoechst H33342)

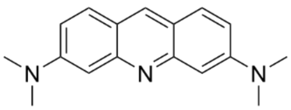

3,6 - acridinamine
(acridin orange)

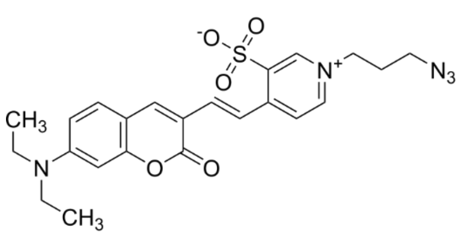

Azid MegaStokes 673

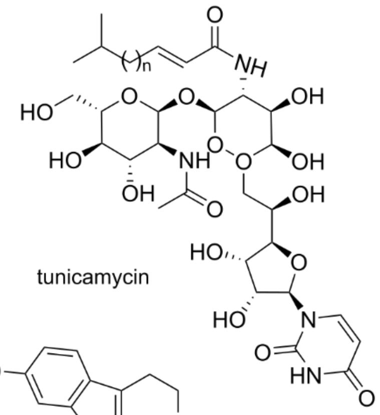

tunicamycin

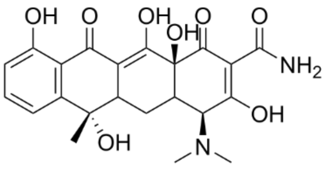

tetracycline

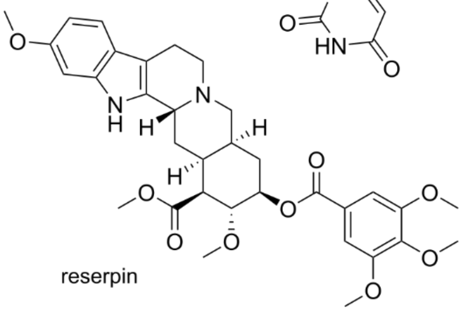

reserpin

Figure S9. Chemical structures of compounds tested in AlbA transcription activation assays.

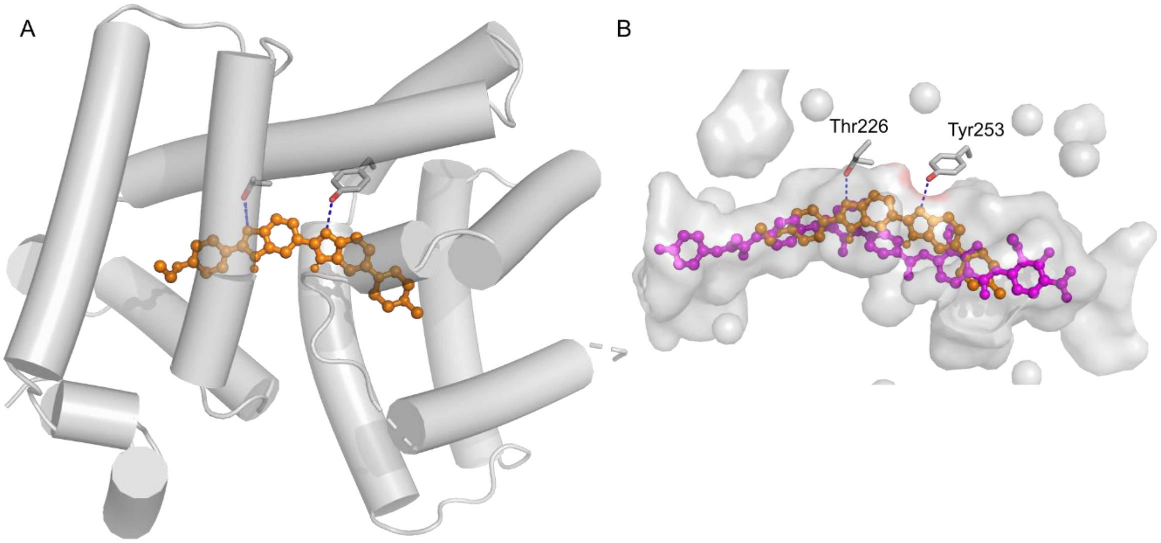

Figure S10. Docking model of AlbA and bisbenzimide. A) Best scoring model (score -12.4) of bisbenzimide (orange) docked to AlbAS (grey, PDB-ID: 6ET8) with Docking Vina. H-bonds to amino acid side-chains (stick representation) are highlighted in blue. B) Surface representation of the albicidin binding pocket with albicidin (magenta) in the crystal structure and docked bisbenzimide (orange). Oxygen atoms on protein side-chains are shown in red.

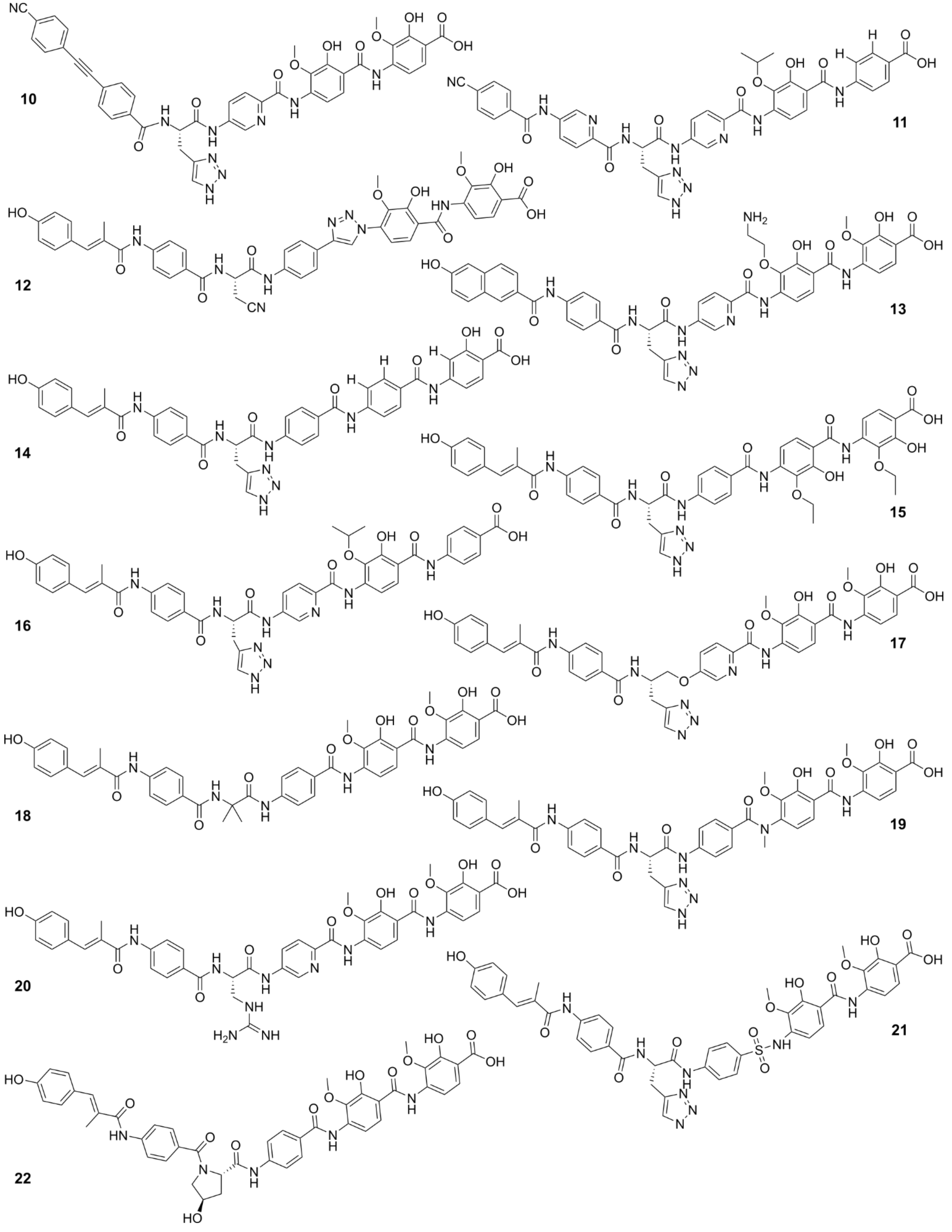

Figure S11. Chemical structures of albicidin derivatives tested in AlbA transcription activation assays.

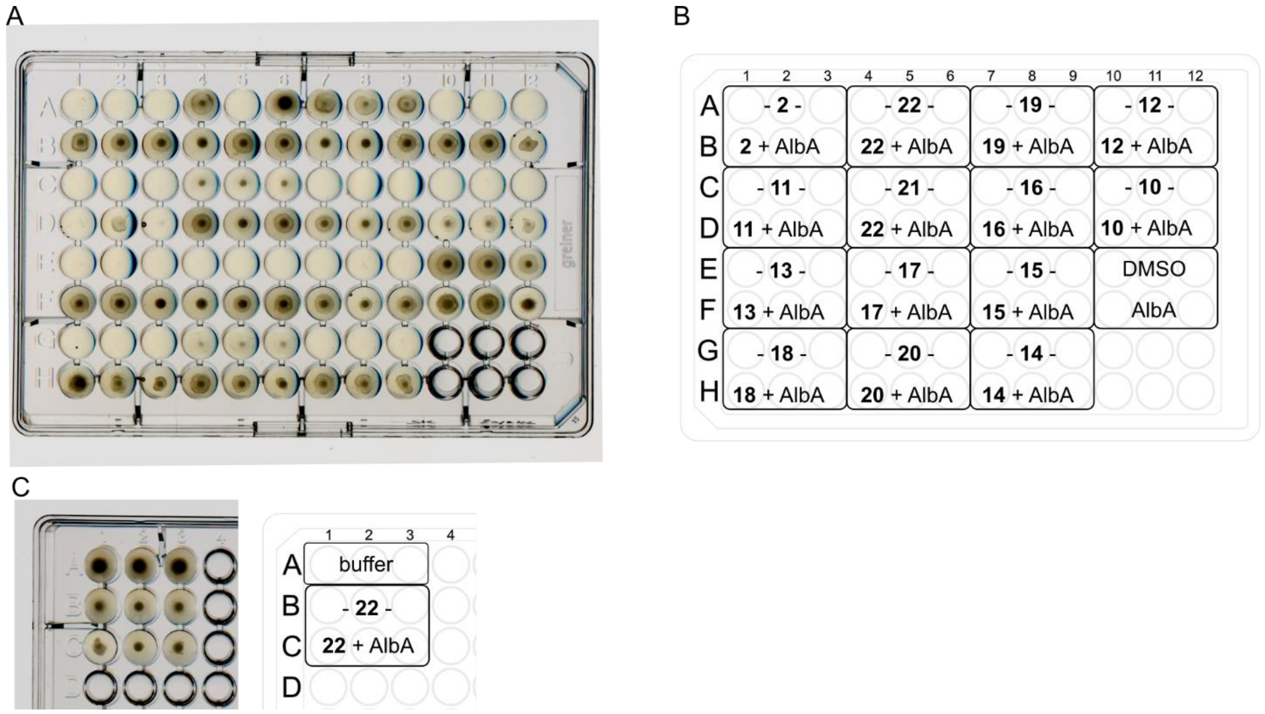

Figure S12. ***E. coli* growth inhibition assays with albicidin derivatives**. A) Microdilution tray with liquid cultures of *E. coli* DSM1116 after incubation with purified AlbA and albicidin derivatives **2**, **10**-**22** and the B) sample loading scheme. The assay was performed in triplicates. C) Repeat of growth inhibition assay with compound **22.**

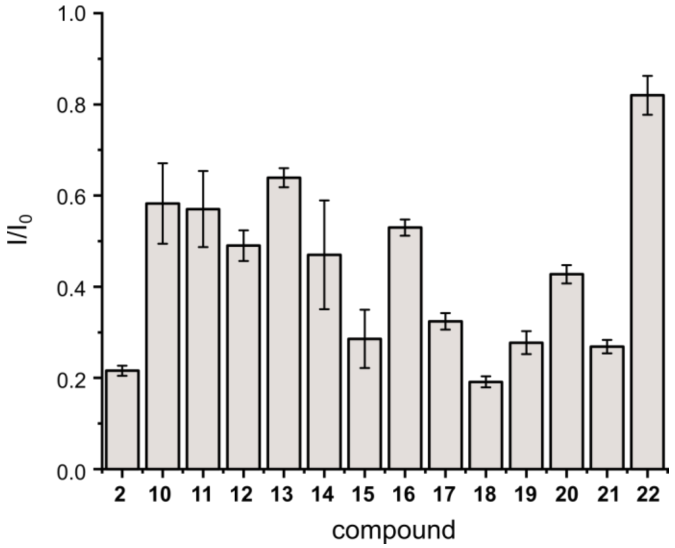

Figure S13. AlbA tryptophan fluorescence quenching by albicidin derivatives. Ratio of fluorescence intensities of AlbA alone (I_0) and AlbA incubated with an equimolar amount of albicidin derivative (I) are shown. Error bars represent the standard deviations, n = 3.

3. Supplementary tables

Table S1 AlbA mutants in literature.

Mutated residues	Residue in full length AlbA	Effect on albicidin binding	Assay	Citation
H78G, H141G and H189G	H205, H268, H316	no effect	Agar diffusion	Weng et al[22]
H125G/A/ L	H252	30% reduced binding	Agar diffusion	Weng et al[22]
H125Y/R	H252	no effect	Agar diffusion	Weng et al[22]
E81-E178 Alanine scan	E208-E305	K106A, W110A, Y113A, L114A, H125A, P134A and W162A showed an effect (see next line)	Agar diffusion	Weng et al[23]
K106A, W110A, Y113A, L114A, H125A, P134A and W162A	K233A, W237A, Y240A, L141A, H252A, P261A and W289A	20-30% reduced binding	Agar diffusion	Weng et al[23]
Y126A	Y253A	60% reduced binding	Agar diffusion	Weng et al[23]
triple mutant N75A/R181A/Q205A	N202A, R308A, Q332A	reduced albicidin neutralization in agar diffusion assay	Agar diffusion	Rostock et al[6]
T99V and T99G	T226A	affects cyclization (V accelerated and G reduced)	Conversion monitored by MS	Sikandar et al[24]
I95G	I222G	reduced cyclization	Conversion monitored by MS	Sikandar et al[24]
triple mutant M131A/Q/K	M258A/Q/K	faster cyclization	Conversion monitored by MS	Sikandar et al[24]

Table S2 Tryptophane fluorescence quenching derived dissociation constants (K_d) and fitting variables of aza-His albicidin (**2**) binding to AlbA mutants.

Protein	K_d [nM]	B_{max}	n	R^2
AlbA	9.4 ± 1.7	0.91 ± 0.01	1.34 ± 0.13	0.981
AlbA-N202A	3.7 ± 0.4	0.92 ± 0.01	0.98 ± 0.08	0.991
AlbA-P209A	12.6 ± 1.7	0.93 ± 0.02	0.95 ± 0.11	0.993
AlbA-P209G	15.6 ± 1.7	0.95 ± 0.02	1.05 ± 0.11	0.994
AlbA-P219A	4.8 ± 0.3	0.77 ± 0.01	1.24 ± 0.08	0.996
AlbA-P219G	26.8 ± 5.0	0.85 ± 0.03	1.13 ± 0.15	0.995
AlbA-H252A	10.9 ± 3.5	0.86 ± 0.07	0.89 ± 0.21	0.951
AlbA-W260A	11.2 ± 0.9	0.94 ± 0.01	1.13 ± 0.10	0.993
AlbA-W289A	27.8 ± 0.2	0.93 ± 0.02	0.95 ± 0.09	0.996
AlbA-Y296A	18.1 ± 1.8	0.90 ± 0.02	0.73 ± 0.05	0.999
AlbA-R308A	2.5 ± 0.7	0.99 ± 0.01	1.07 ± 0.21	0.951
AlbA-Q332A	14.4 ± 2.5	0.96 ± 0.02	0.91 ± 0.09	0.993

Table S3 MIC values of albicidin derivatives.

		MIC [mg L^{-1}]							
**Compound**	**Name**	***E. coli* BW25113**	***E. coli* DH5α**	***E. coli* DSM1116**	***S.* Typhimurium TA100**	***B. subtilis* DSM10**	***M. Luteus* DSM1790**	***M. Phlei* DSM750**	**gyrase inhibition**
**1**	albicidin	0.063	≤0.016	0.063	0.063	0.25	2	2	0
**2**	AF-C-014	≤0.016	n.d.	≤0.016	≤0.016	0.125	0.5	4	0
**3**	AE-001	n.d.	>128	n.d.	n.d.	n.d.	>128	n.d.	n.d.
**4**	BF-001	n.d.	>128	>8.0	>8.0	>8.0	>128	n.d.	n.d.
**5**	KH-602-AD	n.d.	n.d.	n.d.	n.d.	n.d.	n.d.	n.d.	n.d.
**6**	DF-001	n.d.	>128	n.d.	n.d.	n.d.	>128	n.d.	n.d.
**7**	CF-001	n.d.	>128	n.d.	n.d.	n.d.	>128	n.d.	n.d.
**8**	KH-599-BD	n.d.	n.d.	n.d.	n.d.	n.d.	n.d.	n.d.	n.d.
**9**	BE-001	n.d.	>128	n.d.	n.d.	n.d.	>128	n.d.	n.d.
**10**	AF-ABCD-006	≤0.016	n.d.	≤0.016	≤0.016	0.125	0.16	0.25	+
**11**	AF-ABCDEF-001	≤0.016	n.d.	≤0.016	0.016	0.031	≤0.016	0.25	+
**12**	AF-DE-001	1	n.d.	0.5	0.125	1	n.d.	n.d.	-
**13**	AF-ACDE-001	4	n.d.	2	0.5	>8	>8	>8	n.d.
**14**	AF-CEF-009	0.063	n.d.	0.25	0.125	>8	>8	>8	+
**15**	AF-CEF-001	0.016	n.d.	0.016	0.016	0.031	0.5	0.5	-
**16**	AF-CDEF-001	0.031	n.d.	0.031	≤0.016	≤0.016	0.063	0.25	+
**17**	AF-CD-011	0.063	n.d.	0.063	0.03	1	0.5	1	0
**18**	AF-C-005	0.5	n.d.	0.5	-	4	1	2	+
**19**	AF-CDE-001	>8	n.d.	>8	>8	>8	>8	>8	-
**20**	AF-CD-013	1	n.d.	4	0.063	>8	>8	>8	+
**21**	AF-CD-006	>8	n.d.	>8	2	>8	>8	>8	-
**22**	AF-C-008	n.d.	n.d.	>8	>8	>8	>16	>16	-

Abbreviations: gyrase inhibitory activity is shown as 0, equal to the activity of albicidin, -, lower than the activity of albicidin or +, higher than the activity of albicidin. n.d., not determined.

Table S4 List of plasmids in this study

**Plasmid name**	**Origin**	**Purpose**
pET28a-AlbAL	Rostock et al.[6]	protein expression
pET15b-AlbA	this study	protein expression
pET15b-ΔHisAlbA	this study	protein expression in reporter assay
pCS-PesaRlux	Shong et al.[2] via Addgene	template
pGEX-iluxCDABE-frp	Gregor et al.[4]	template
pCS-ilux	this study	reporter plasmid
pCS-MCS-RBS-ilux	this study	reporter plasmid
pCS-T7-ilux	this study	reporter plasmid
pCS-pAlbA-ilux	this study	reporter plasmid
pET28a-AlbA-N202A	this study	protein expression
pET28a-AlbA-P209A	this study	protein expression
pET28a-AlbA-P209G	this study	protein expression
pET28a-AlbA-P219A	this study	protein expression

pET28a-AlbA-P219G	this study	protein expression
pET28a-AlbA-H252A	this study	protein expression
pET28a-AlbA-W260A	this study	protein expression
pET28a-AlbA-W289A	this study	protein expression
pET28a-AlbA-Y296A	this study	protein expression
pET28a-AlbA-R308A	this study	protein expression
pET28a-AlbA-Q332A	this study	protein expression
pET15b-ΔHisAlbA-N202A	this study	protein expression in reporter assay
pET15b-ΔHisAlbA-P209A	this study	protein expression in reporter assay
pET15b-ΔHisAlbA-P209G	this study	protein expression in reporter assay
pET15b-ΔHisAlbA-P219A	this study	protein expression in reporter assay
pET15b-ΔHisAlbA-P219G	this study	protein expression in reporter assay
pET15b-ΔHisAlbA-H252A	this study	protein expression in reporter assay
pET15b-ΔHisAlbA-W260A	this study	protein expression in reporter assay
pET15b-ΔHisAlbA-W289A	this study	protein expression in reporter assay
pET15b-ΔHisAlbA-Y296A	this study	protein expression in reporter assay
pET15b-ΔHisAlbA-R308A	this study	protein expression in reporter assay
pET15b-ΔHisAlbA-Q332A	this study	protein expression in reporter assay

Table S5 Primers and oligonucleotides used for cloning of the transcription reporter system.

Vector construct	oligonucleotide name	Sequence 5'-3'
pCS-ilux	pCS-fw	CCGAAGCGTTTGATAGTTGA
	pCS-rev	GACGTCGGAATTGCCAGCTG
	Ilux-fw	CGCCCCAGCTGGCAATTCCGACGTCATGACTAAAAAAATTTCATTCATTATTAC-CGGCCA
	Ilux-rev	GATATCAACTATCAAACGCTTCGGTTACCTTCTGGCAAGGCCCTTG
pCS-MCS-RBS- ilux	pCS-MCS-RBS-fwt	CTCGAGTTAGGATCCTTTCACACAGGAAACTTAGTCCATGACTAAAAAAATTTCATT-CATTATTACCGGCCA
	pCS-MCS-RBS-fw	ATGACTAAAAAAATTTCATTCATTATTACCGGCCA
	pCS-MCS-RBS-revt	GGACTAAGTTTCCTGTGTGAAAGGATCCTAACTCGAGGACGTCGGAATTGCCAGCTG
	pCS-MCS-RBS-rev	GACGTCGGAATTGCCAGCTGGGGC
pCS-pAlbA- ilux	pAlbA-fw	CTTCACCTCGAGTTAGCGCTTGACCCTGACGTCACGTCAGGCAGCAGAGTCGTG-GATCCTCTAGT
	pAlbA-rev	ACTAGAGGATCCACGACTCTGCTGCCTGACGTGACGTCAGGGTCA-AGCGCTAACTCGAGGTGAAG
pCS-pT7- ilux	pT7-fw	CTTCACCTCGAGTGCACGTCGGCATTGGTACCTAATACGACTCACTATAGG-GATCCTCTAGT
	pT7-rev	ACTAGAGGATCCCTATAGTGAGTCGTATTAGGTACCAAT-GCCGACGTGCACTCGAGGTGAAG
pET-15b-AlbA	AlbA_XhoI_fw	TTATATCTCGAGATGCTGATTCAGGTTGGT
	AlbA_BamHI_rev	TTATATGGATCCTTATTCTGCGGCAGGA
pET-15b-deltaHis-AlbA	deltaHis-AlbA-fwt	TTGTTTAACTTTAAGAAGGAGATATACCATGCTGATTCAGGTTGGTGAA
	deltaHis-AlbA-fw	ATGCTGATTCAGGTTGGTGAACTGG
	deltaHis-AlbA-revt	GGTATATCTCCTTCTTAAAGTTAAACAAAATTATTTCTAGAGGGGAATT
	deltaHis-AlbA-rev	AATTATTTCTAGAGGGGAATTGTTATCCGCT

Table S6 Primers used for mutagenesis. Triplets containing mutations for amino acid exchange are highlighted in pink.

Mutant	Name	Sequenz 5'-3'
AlbA N202A	fw_t	GAA TTT CTG ACC CGT CTG GCT GAA ATG CAT GCA GCA GAA CCG CAG ATG CG
	fw	GCA GCA GAA CCG CAG ATG CG
	rev_t	ATG CAT TTC AGC CAG ACG GGT CAG AAA TTC CGG ACG ACC TGC GGT ATC CT
	rev	CGG ACG ACC TGC GGT ATC CT
AlbA P209A	fw_t	GAA TGA AAT GCA TGC AGC AGA AGC TCA GAT GCG TGA ACA GAC CGG TGT TAC TCC G
	fw	GCG TGA ACA GAC CGG TGT TAC TCC G
	rev_t	ATC TGA GCT TCT GCT GCA TGC ATT TCA TTC AGA CGG GTC AGA AAT TCC GGA CG
	rev	AGA CGG GTC AGA AAT TCC GGA CG
AlbA P209G	fw_t	GAA TGA AAT GCA TGC AGC AGA AGG TCA GAT GCG TGA ACA GAC CGG TGT TAC TCC G
	fw	GCG TGA ACA GAC CGG TGT TAC TCC G
	rev_t	ATC TGA CCT TCT GCT GCA TGC ATT TCA TTC AGA CGG GTC AGA AAT TCC GGA CG
	rev	AGA CGG GTC AGA AAT TCC GGA CG
AlbA P219A	fw_t	TGT TAC TGC TGA AAT GAT TGA TTT TAT CAC CCG TGC ATT TGC CGA AAG CA
	fw	CCG TGC ATT TGC CGA AAG CAA ACT GG
	rev_t	GTG ATA AAA TCA ATC ATT TCA GCA GTA ACA CCG GTC TGT TCA CGC ATC TG
	rev	CCG GTC TGT TCA CGC ATC TGC GGT
AlbA P219G	fw_t	TGT TAC TGG TGA AAT GAT TGA TTT TAT CAC CCG TGC ATT TGC CGA AAG CA
	fw	CCG TGC ATT TGC CGA AAG CAA ACT GG
	rev_t	GTG ATA AAA TCA ATC ATT TCA CCA GTA ACA CCG GTC TGT TCA CGC ATC TG
	rev	CCG GTC TGT TCA CGC ATC TGC GGT
AlbA H252A	fw_t	AGG AAC TGG CAT TTA CCC GTC AGG CTT ATT TTG ATC GTC TGA TGG AAT GG
	fw	TTG ATC GTC TGA TGG AAT GGC CTG C
	rev_t	AAT AAG CCT GAC GGG TAA ATG CCA GTT CCT CTG CAT TCA GAT AAC GTG CC
	rev	CTG CAT TCA GAT AAC GTG CCC AAA TTG CC
AlbA W260A	fw_t	ATT TTG ATC GTC TGA TGG AAG CTC CTG CAC TGG TTG CCG ATC TGC ATC
	fw	TGG TTG CCG ATC TGC ATC GTG C
	rev_t	GTG CAG GAG CTT CCA TCA GAC GAT CAA AAT AAT GCT GAC GGG TAA ATG CC
	rev	AAT GCT GAC GGG TAA ATG CCA GTT CCT C
AlbA W289A	fw_t	GCG TGC TCT GGC ACT GTT TCA GAG CTA TG CAG GTA AAG ATG CAC AGA CC
	fw	CAG GTA AAG ATG CAC AGA CCC AGC A
	rev_t	CAT AGC TCT GAA ACA GTG CCA GAG CAC GC TGG GCC AGC TGC TGA CCT TC
	rev	TGG GCC AGC TGC TGA CCT TC
AlbA Y296A	fw_t	GGC ACT GTT TCA GAG CGC TGC AGG TAA AGA TGC ACA GAC CCA GCA GAA AT
	fw	TGC ACA GAC CCA GCA GAA AT TTC G
	rev_t	TCT TTA CCT GCA GCG CTC TGA AAC AGT GCC AGC CAA CGC TGG GCC
	rev	AGC CAA CGC TGG GCC AGC
AlbA R308A	fw_t	GCA GAA ATT TGC TTA TGC AAT GGA ACA AGA ACC GCA TCT GAT GAA AGG CA
	fw	ACC GCA TCT GAT GAA AGG CAC CT
	rev_t	TCT TGT TCC ATT GCA TAA GCA AAT TTC TGC TGG GTC TGT GCA TCT TTA CCT
	rev	TGG GTC TGT GCA TCT TTA CCT GCA
AlbA Q332A	fw_t	AGC TGG CTG GCT CAG GCA ATT GGT GTT ATG ATG CGT CAG GCA CAG GGT
	fw	ATG CGT CAG GCA CAG GGT CC
	rev_t	CAT AAC ACC AAT TGC CTG AGC CAG CCA GCT CAG CAC TTC GCT GGT CA
	rev	CAG CAC TTC GCT GGT CAT CCA GG

4. Spectra

ABCD fragment (5)

^{1}H-NMR

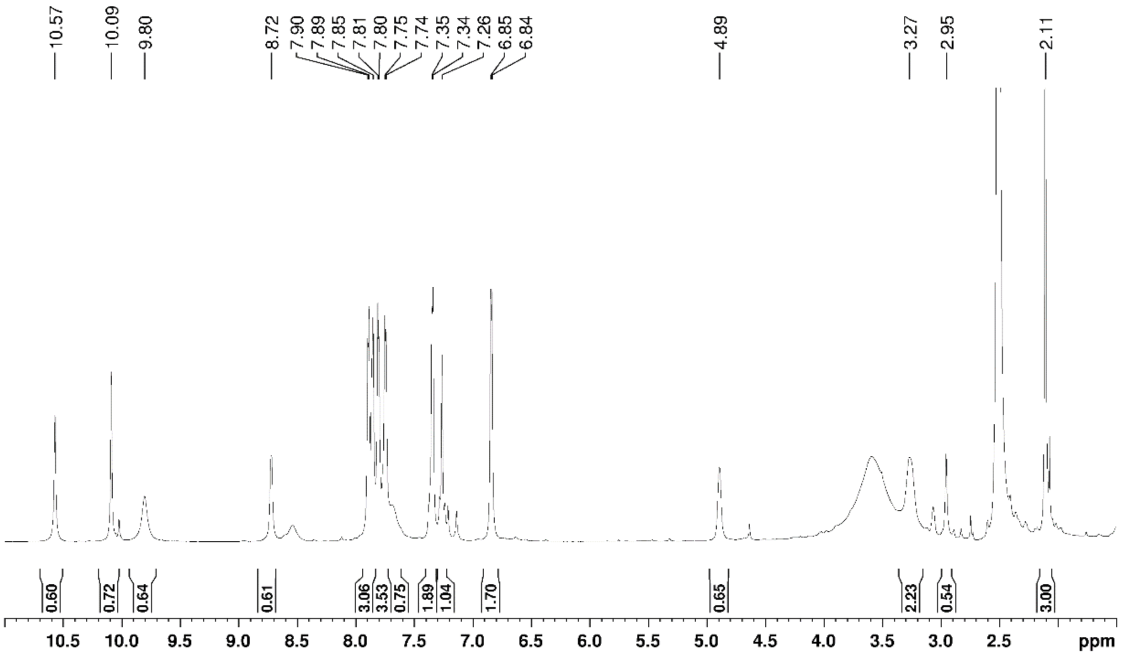

(^{1}H,^{13}C)-HSQC NMR

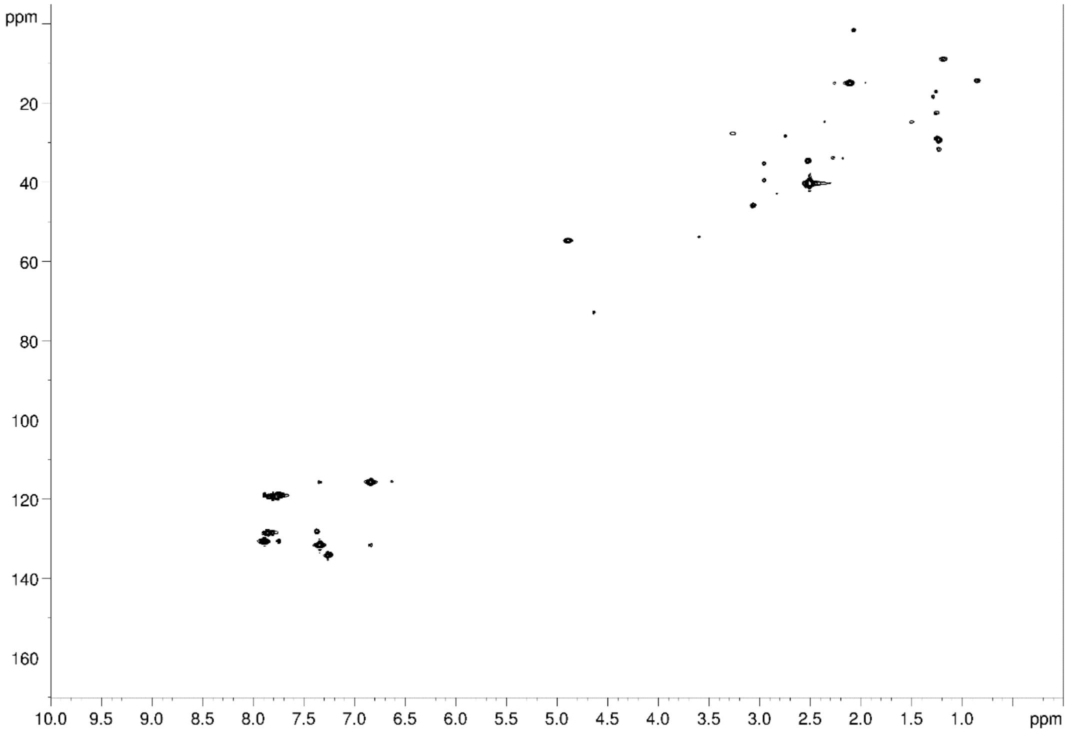

Boc-C(Pom)D-OMe (8b)
^{1}H-NMR

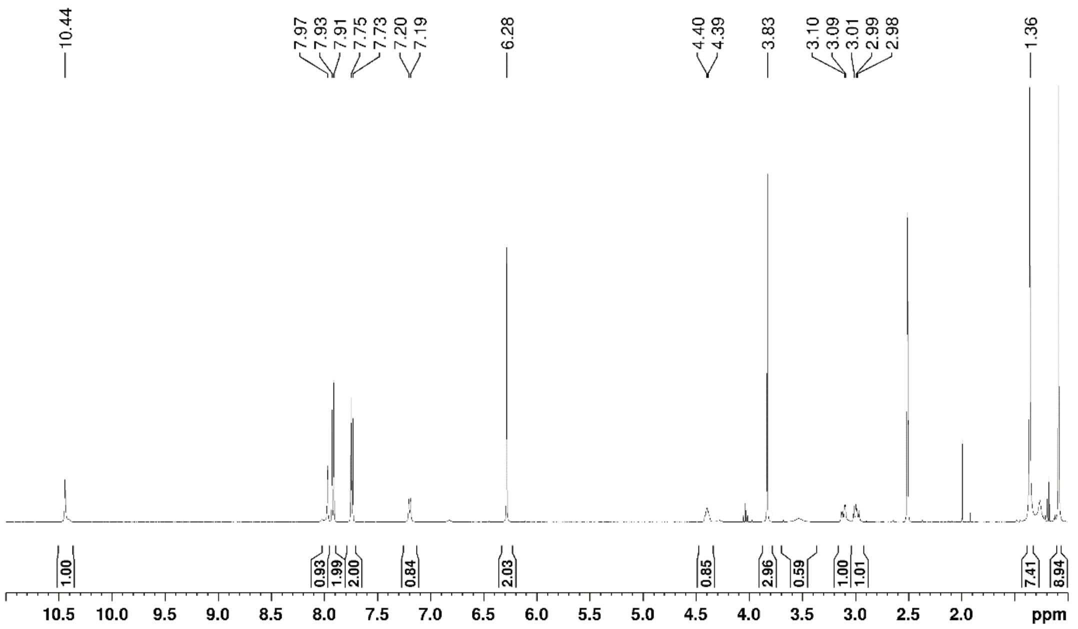

^{13}C-NMR

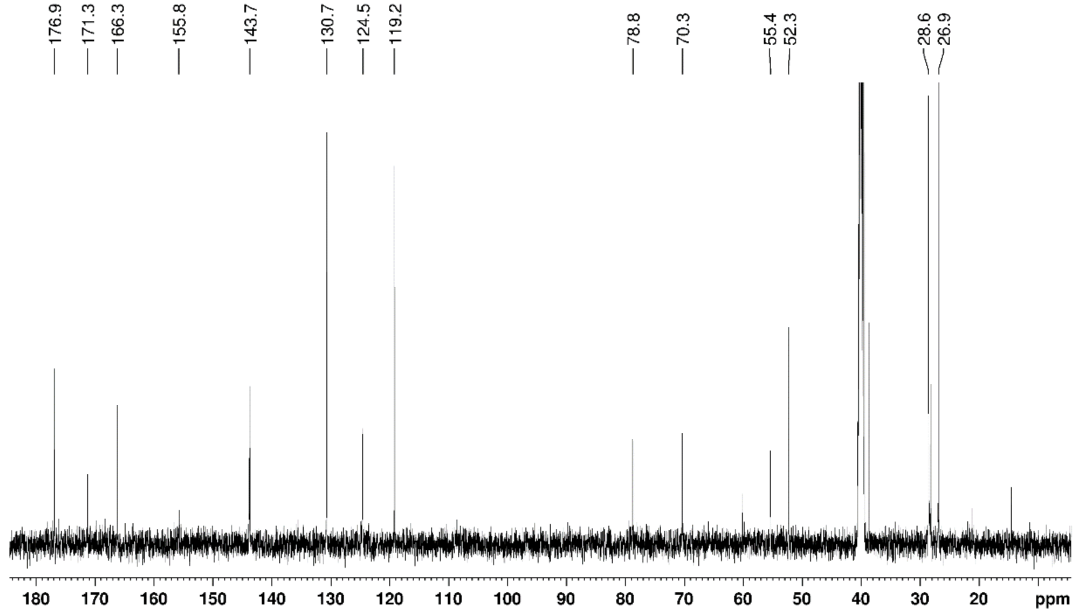

BCD fragment (8)
^{1}H-NMR

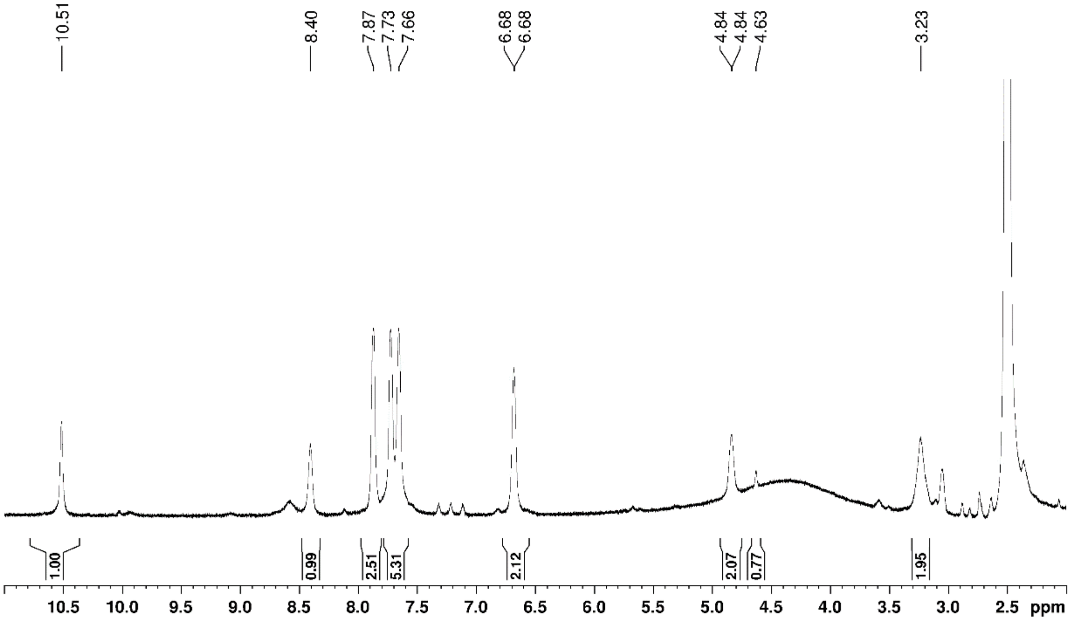

(^{1}H,^{13}C)-HMQC NMR

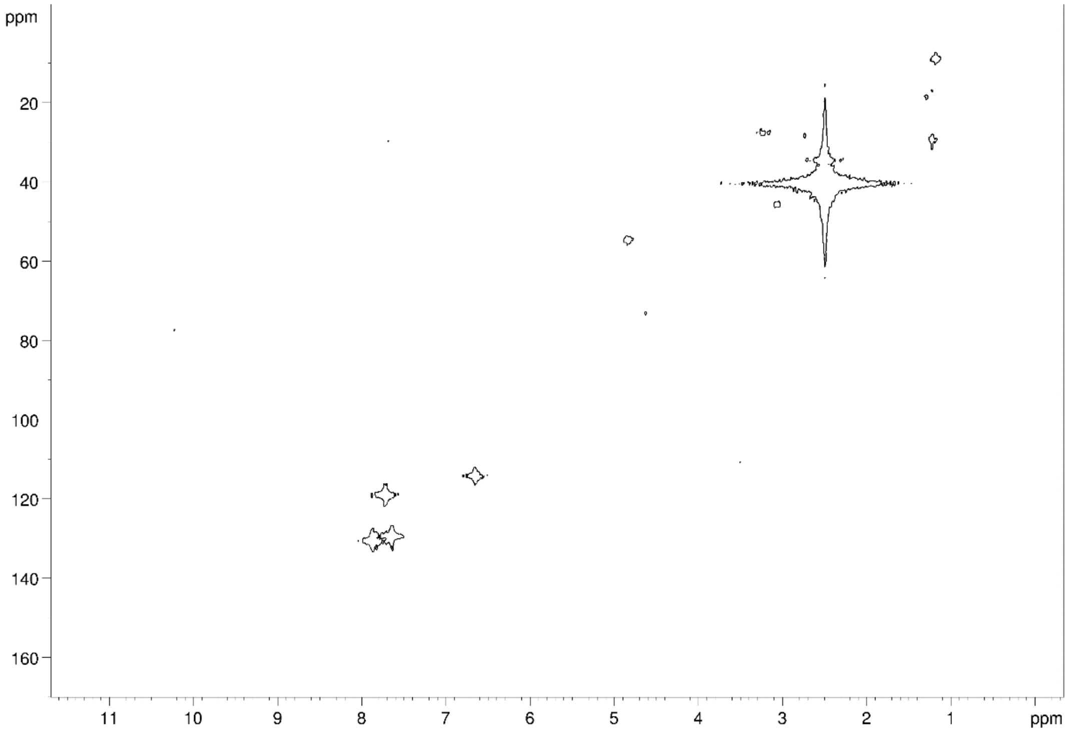

B(Pyr)-C(azaHis)-D(Pyr)-E(*i*Pr)-F(*p*ABA) derivative 16
^{1}H-NMR

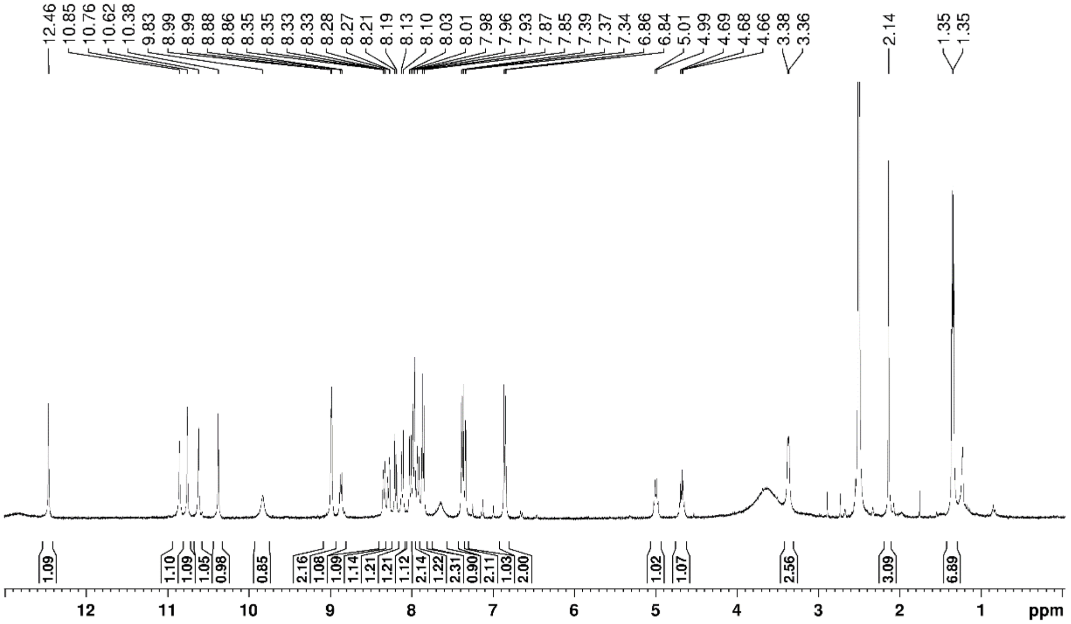

(^{1}H,^{13}C)-HSQC NMR

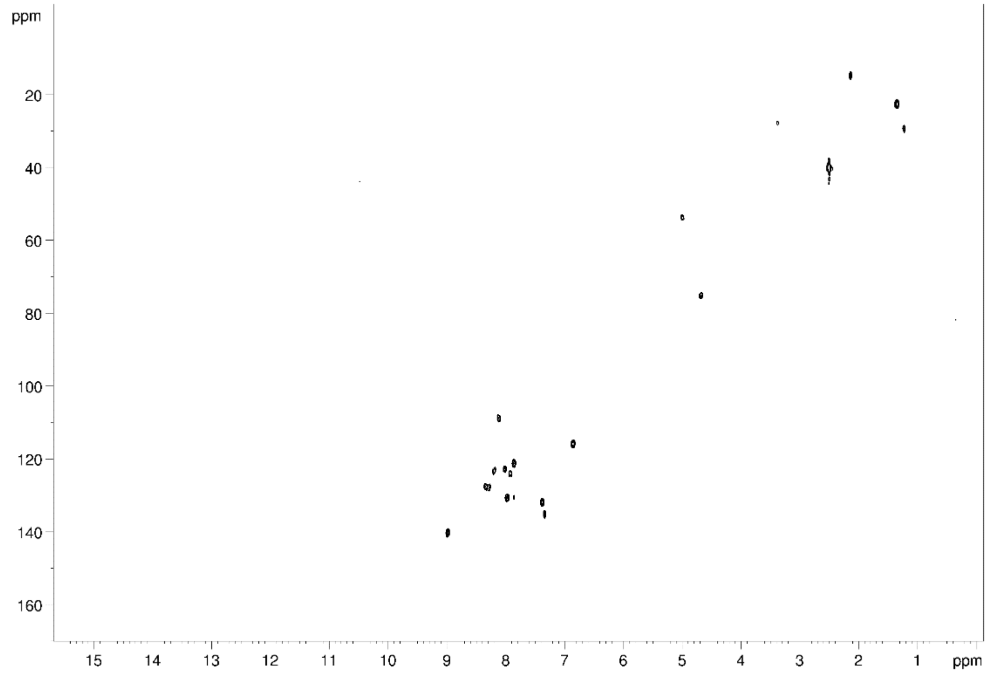

tert-Butyl-(_S_)-(1-hydroxypent-4-yn-2-yl)carbamate (17b)

^{1}H-NMR

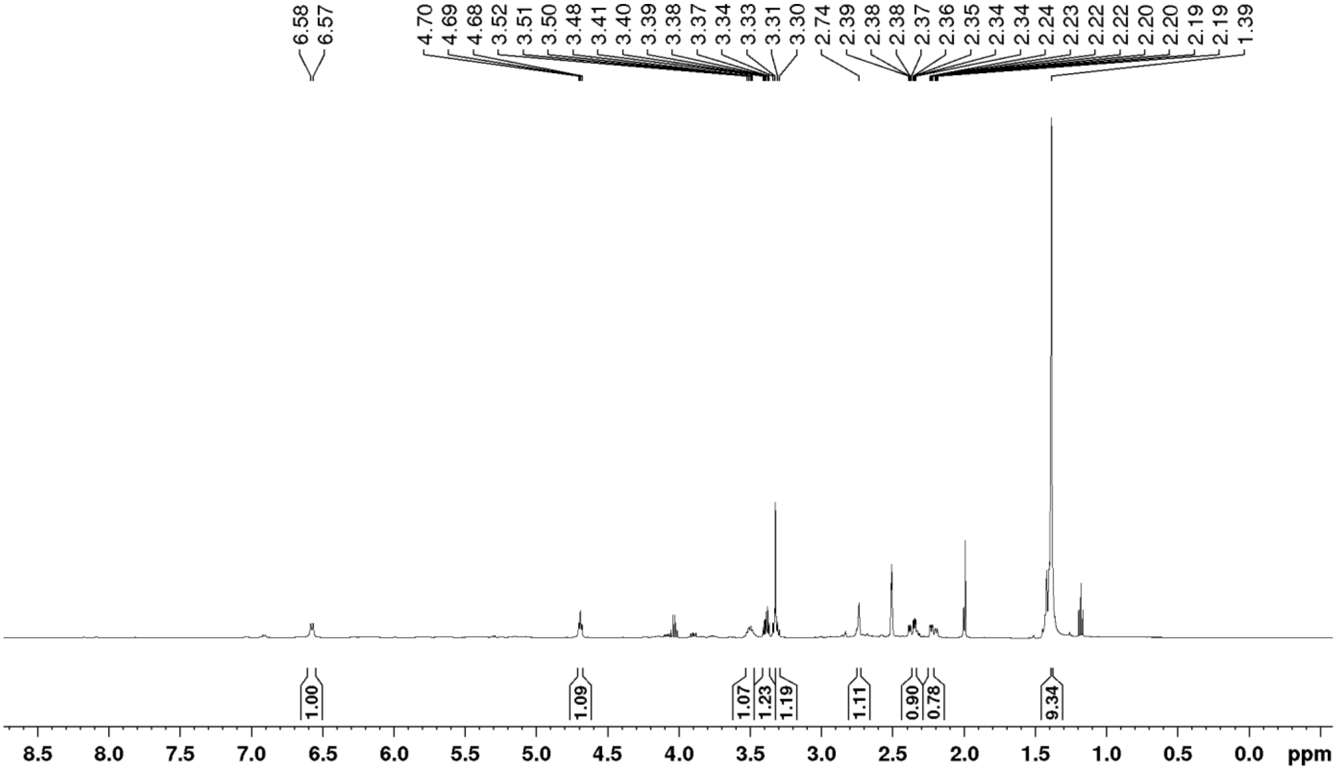

^{13}C-NMR

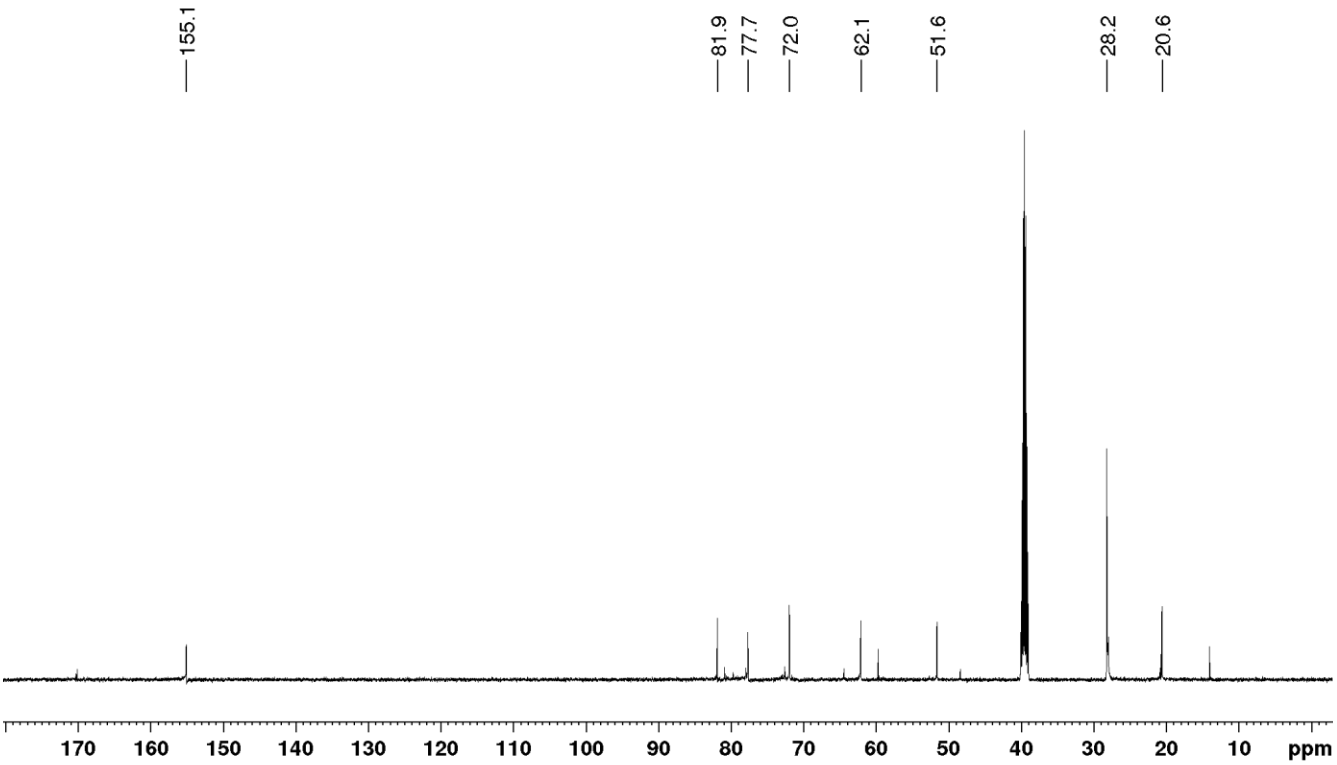

(*S*)-2-((*tert*-butoxycarbonyl)amino)pent-4-yn-1-yl4-methylbenzene sulfonate (17c)

^{1}H-NMR

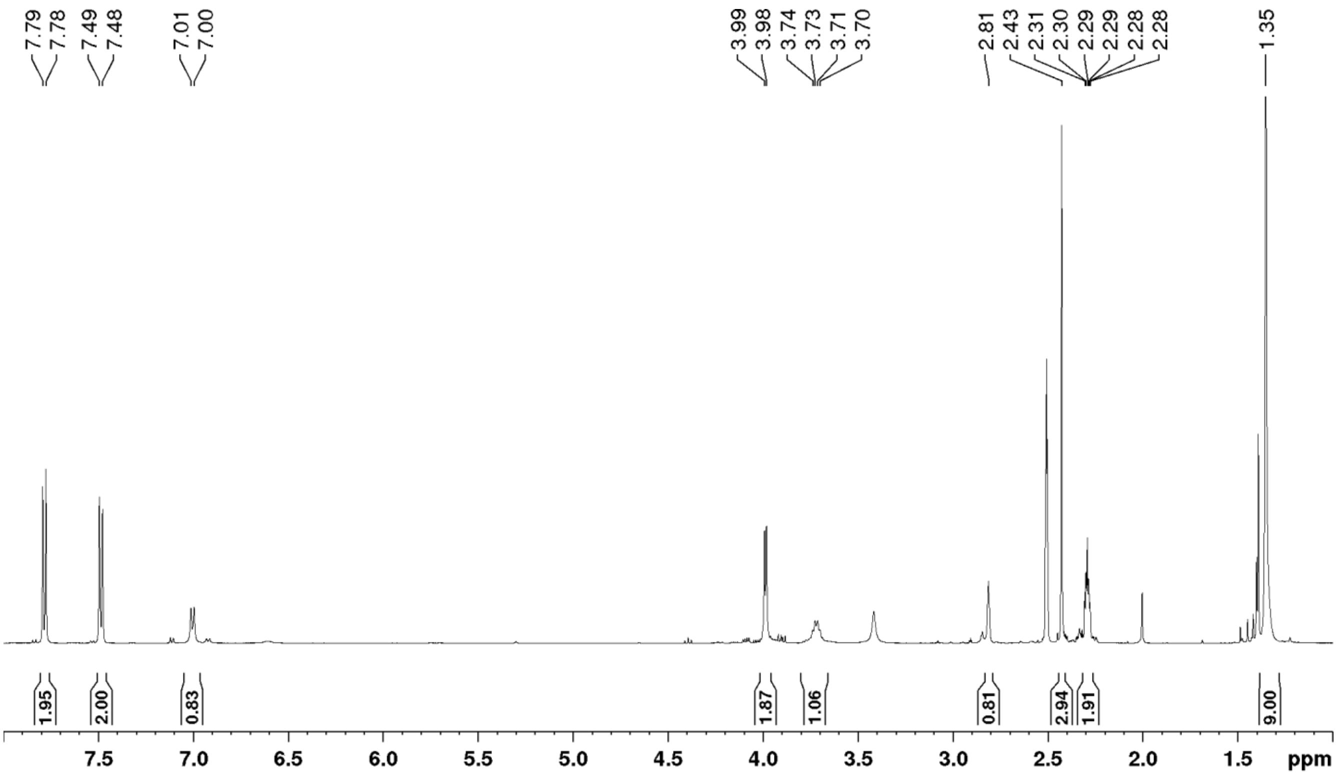

^{13}C-NMR

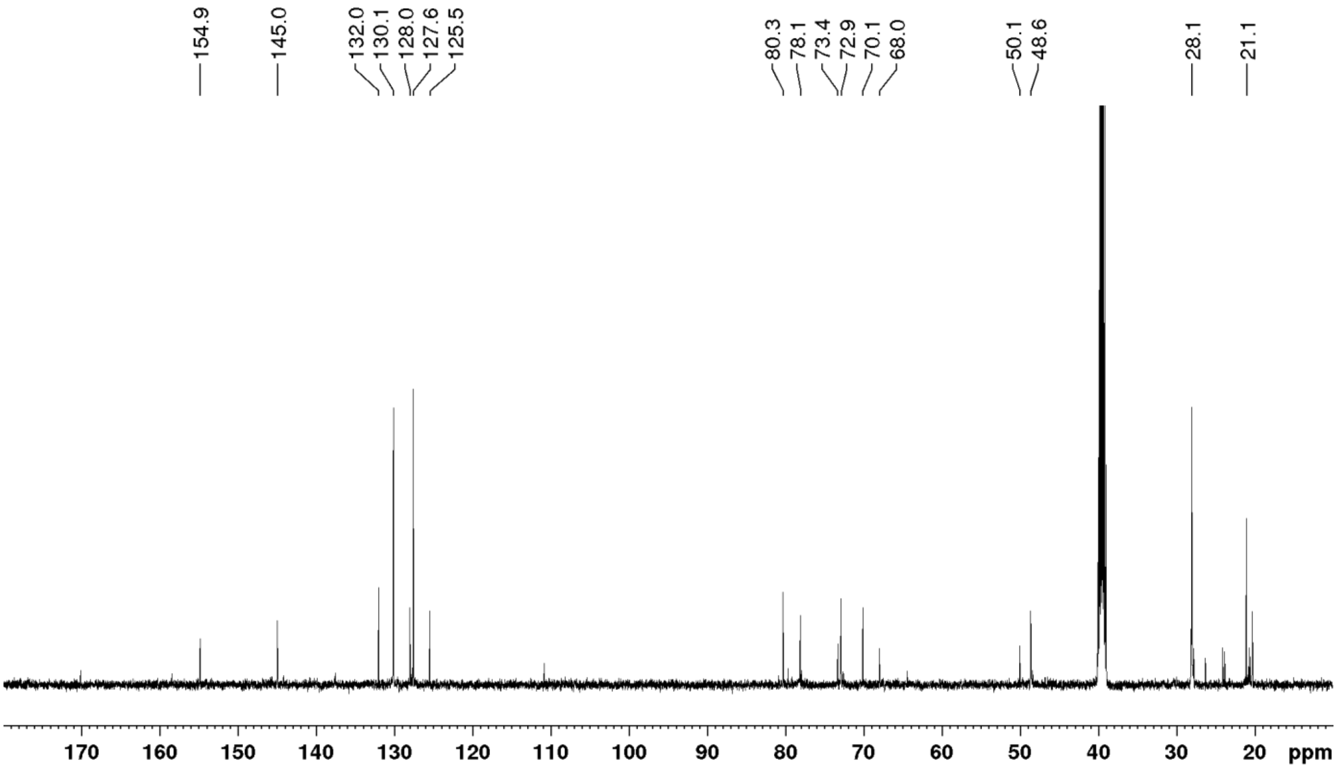

Allyl-5-(allyloxy)picolinate (17e)

^{1}H-NMR

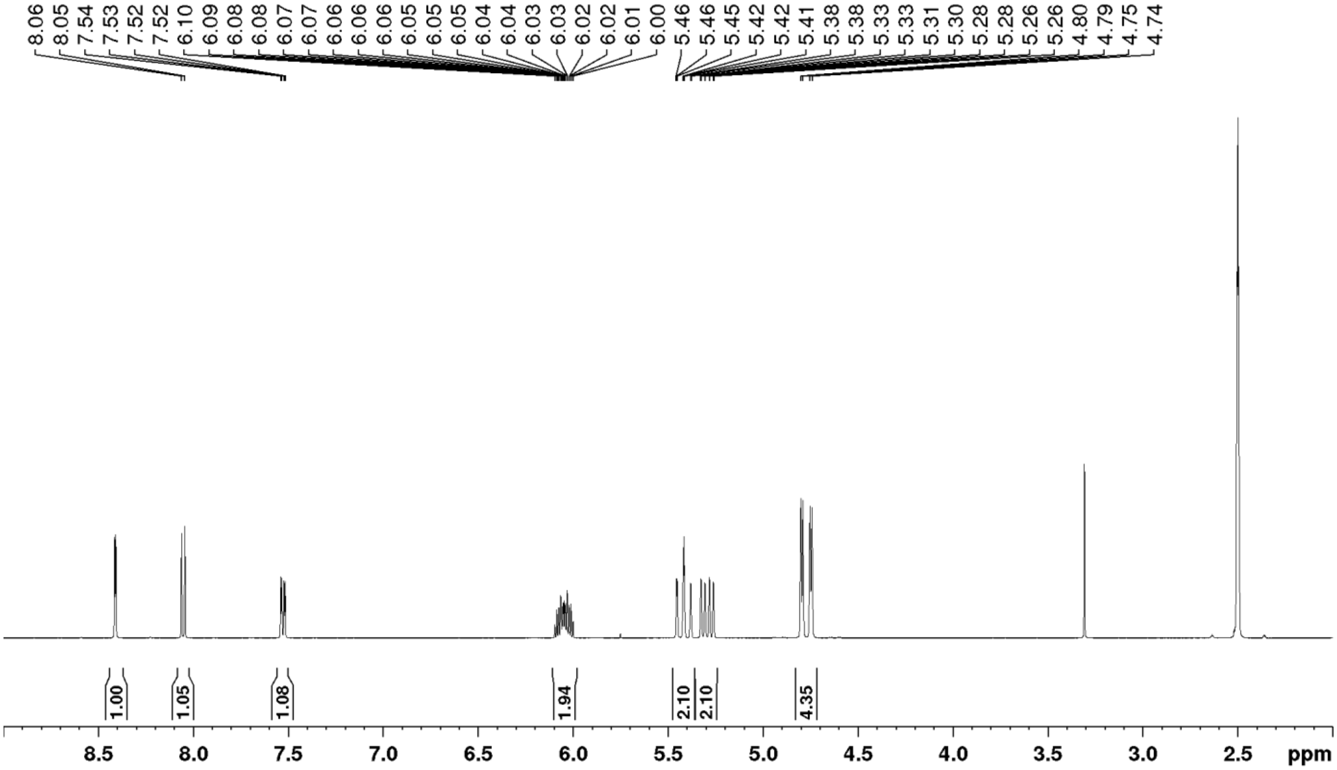

^{13}C-NMR

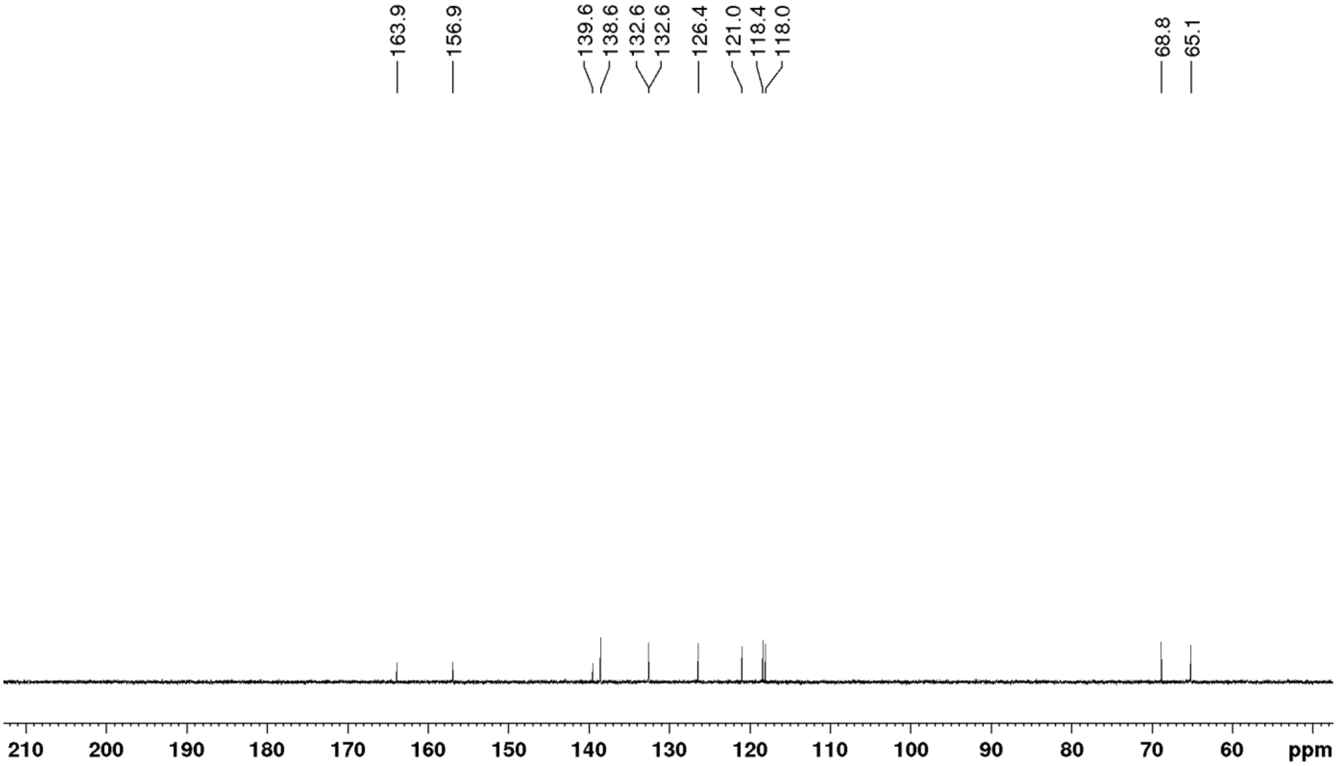

5-(Allyloxy)picolinic acid (17f)

^{1}H-NMR

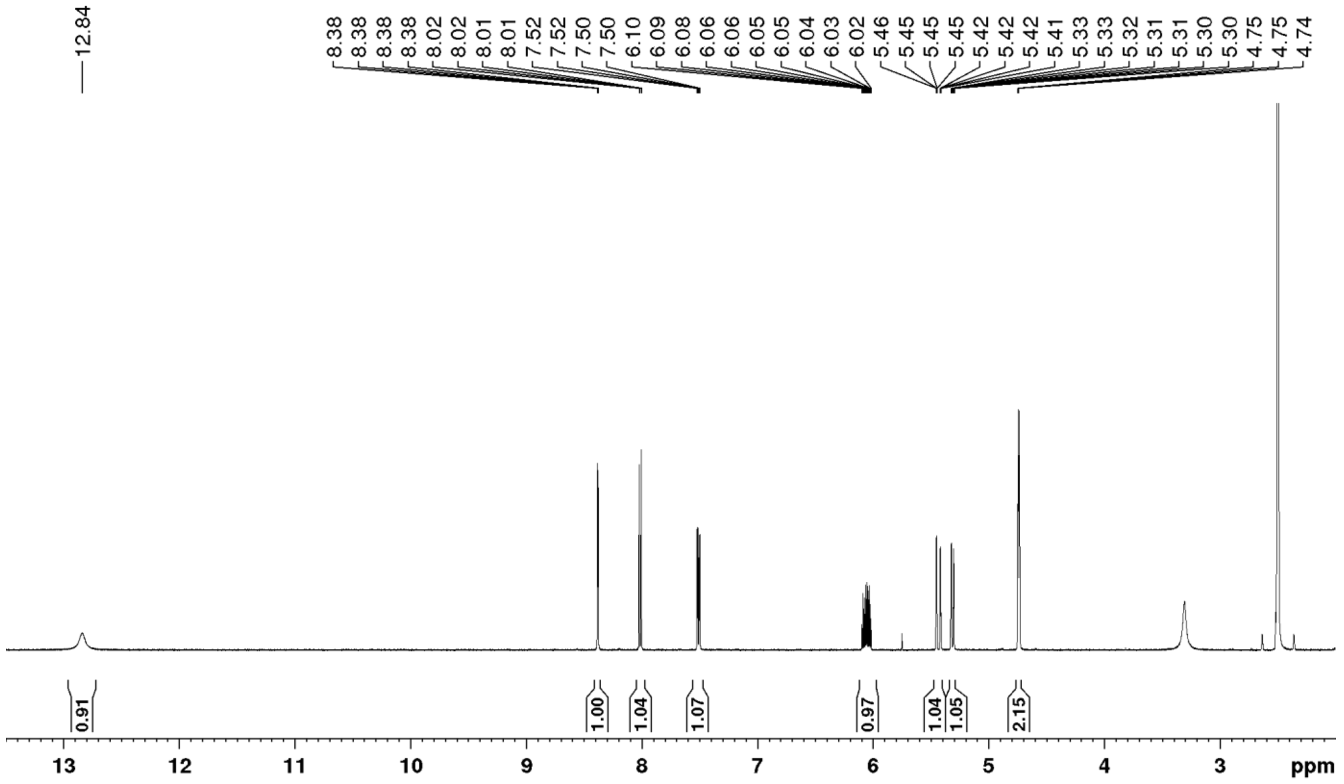

^{13}C-NMR

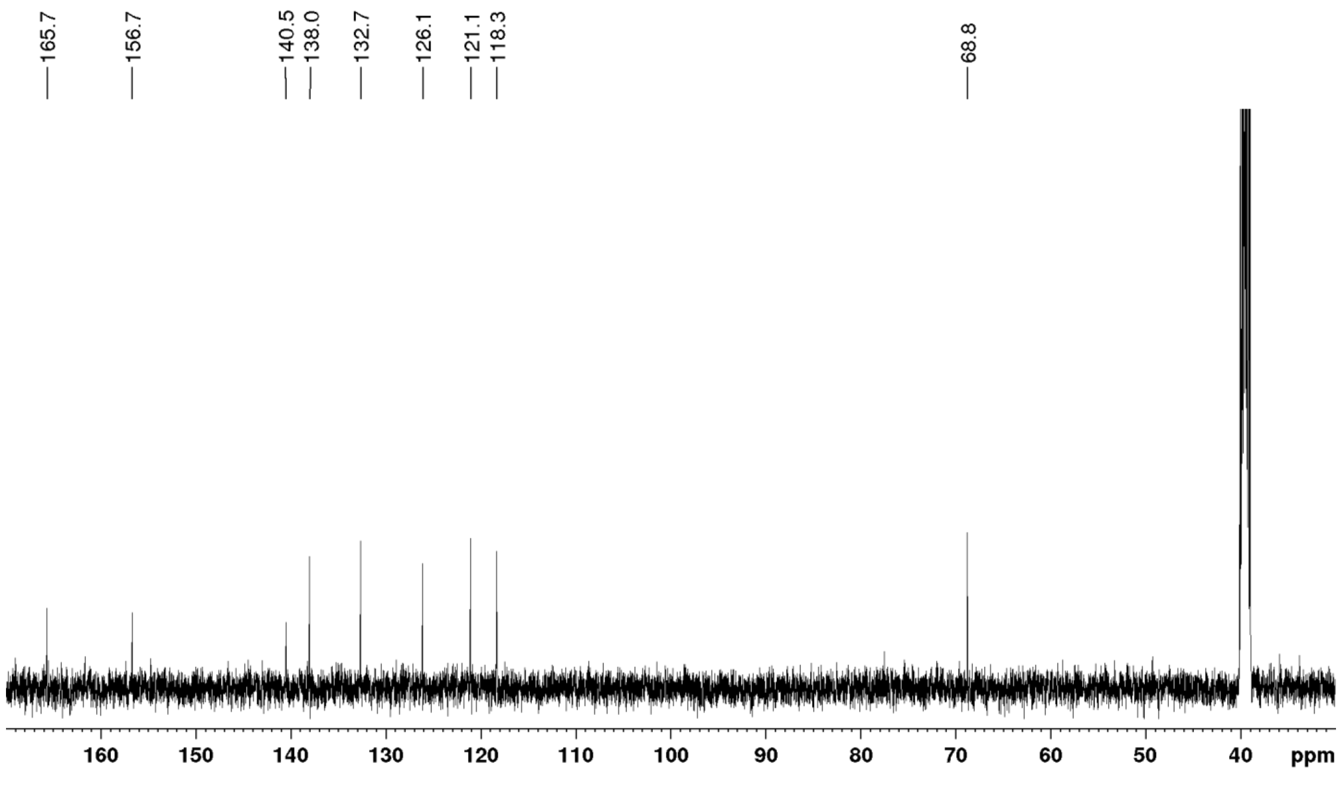

Allyloxy-D(N)-E(OBn)-F(OBn, OBn) (17h)

^{1}H-NMR

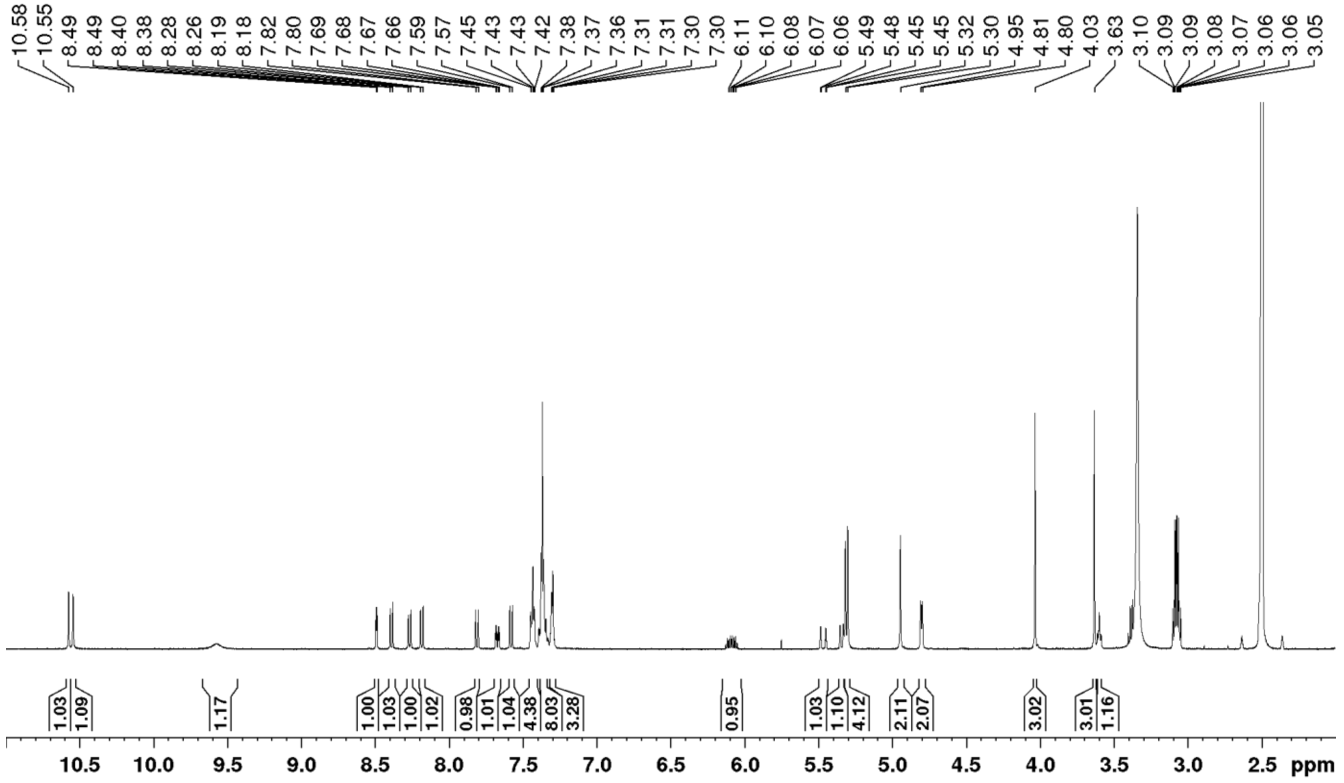

Hydroxy-D(N)-E(OBn)-F(OBn, OBn) (17i)

^{1}H-NMR

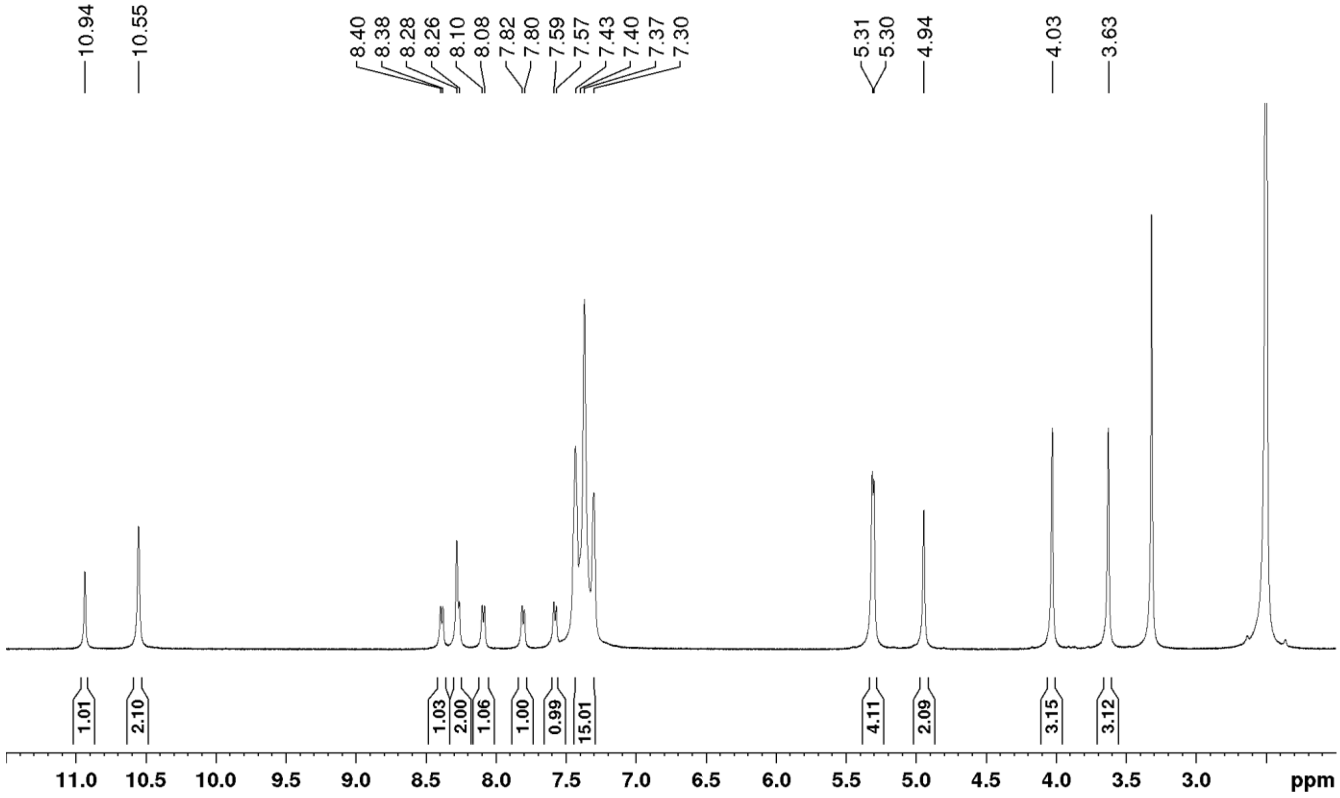

^{13}C-NMR

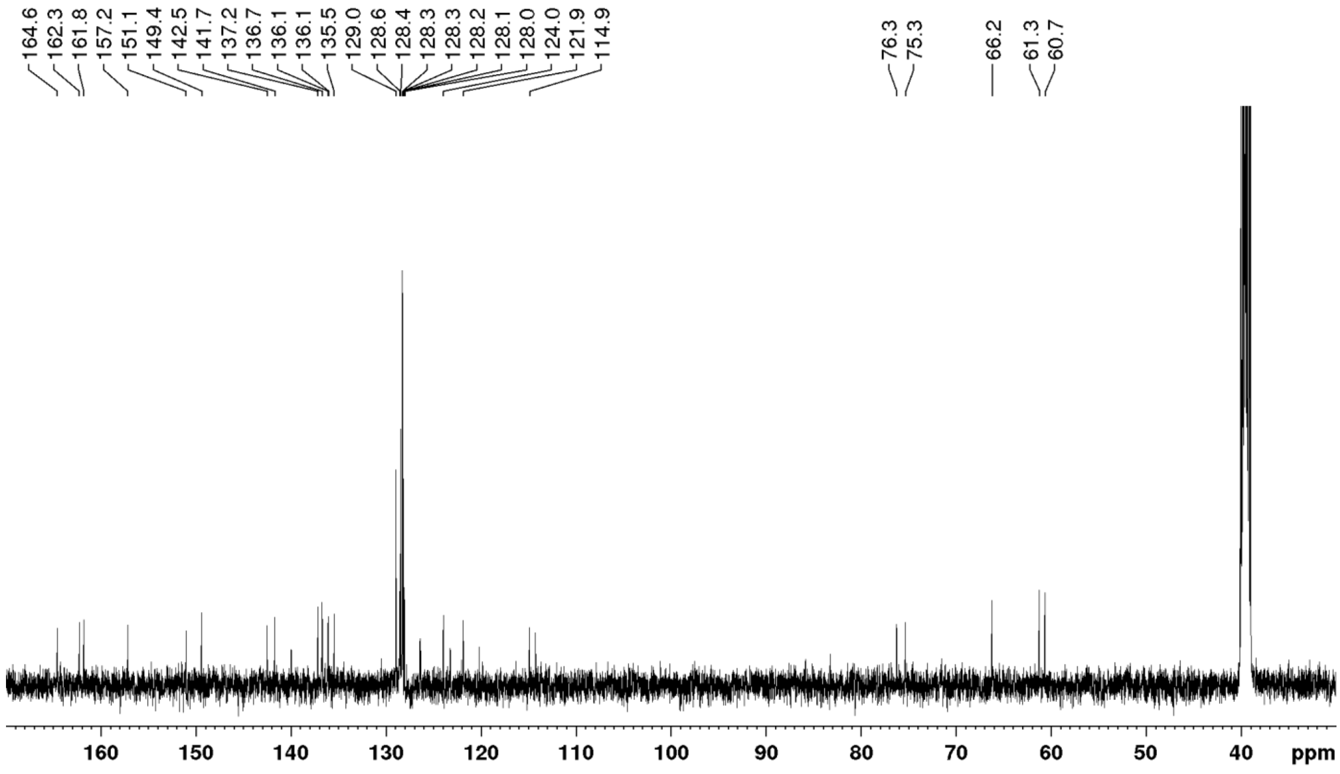

BocHN-CH(propargyl)-CH$_2$O-D(N)-E(OBn)-F(OBn, OBn) (17j)

^{1}H-NMR

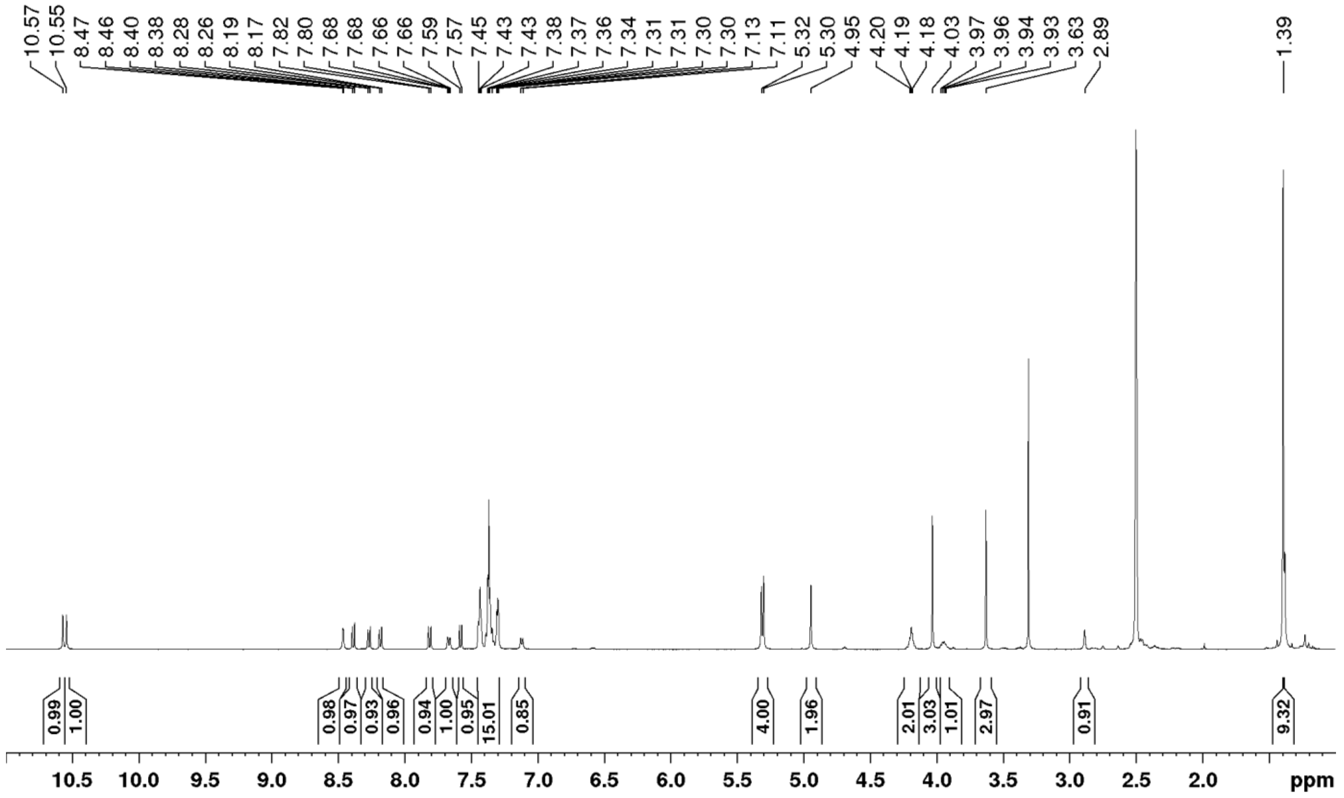

(^{1}H,^{13}C)-HSQC ed NMR

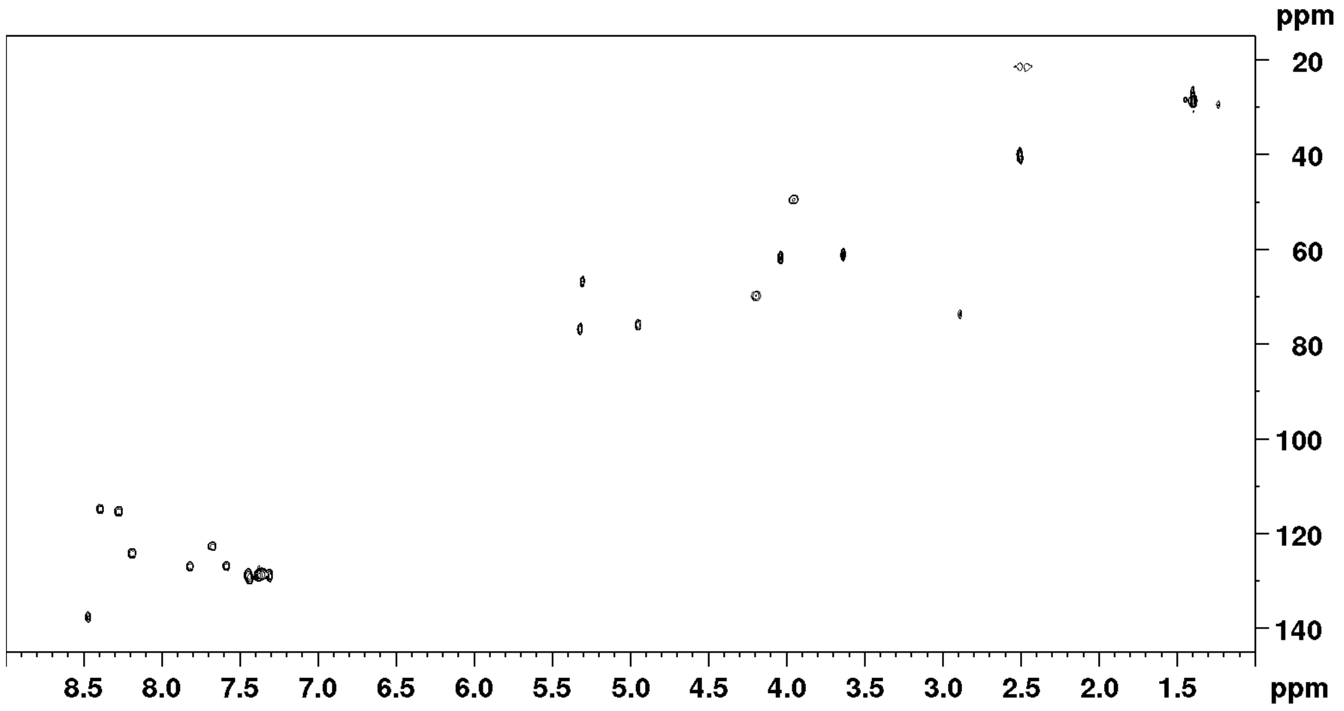

Boc-AzaHis(POM)-D(N)-E(OBn)-F(OBn, OBn) (17k)

^{1}H-NMR

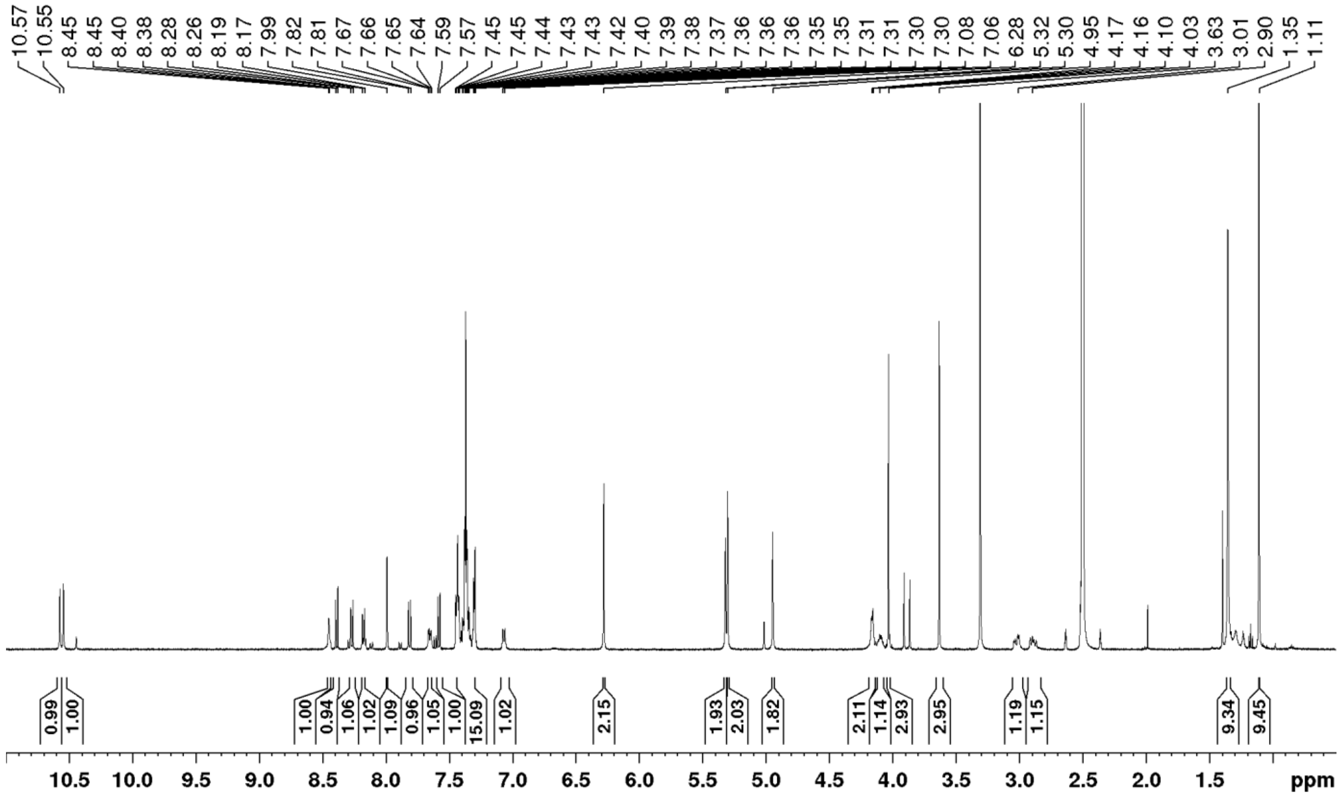

(^{1}H,^{13}C)-HSQC ed NMR

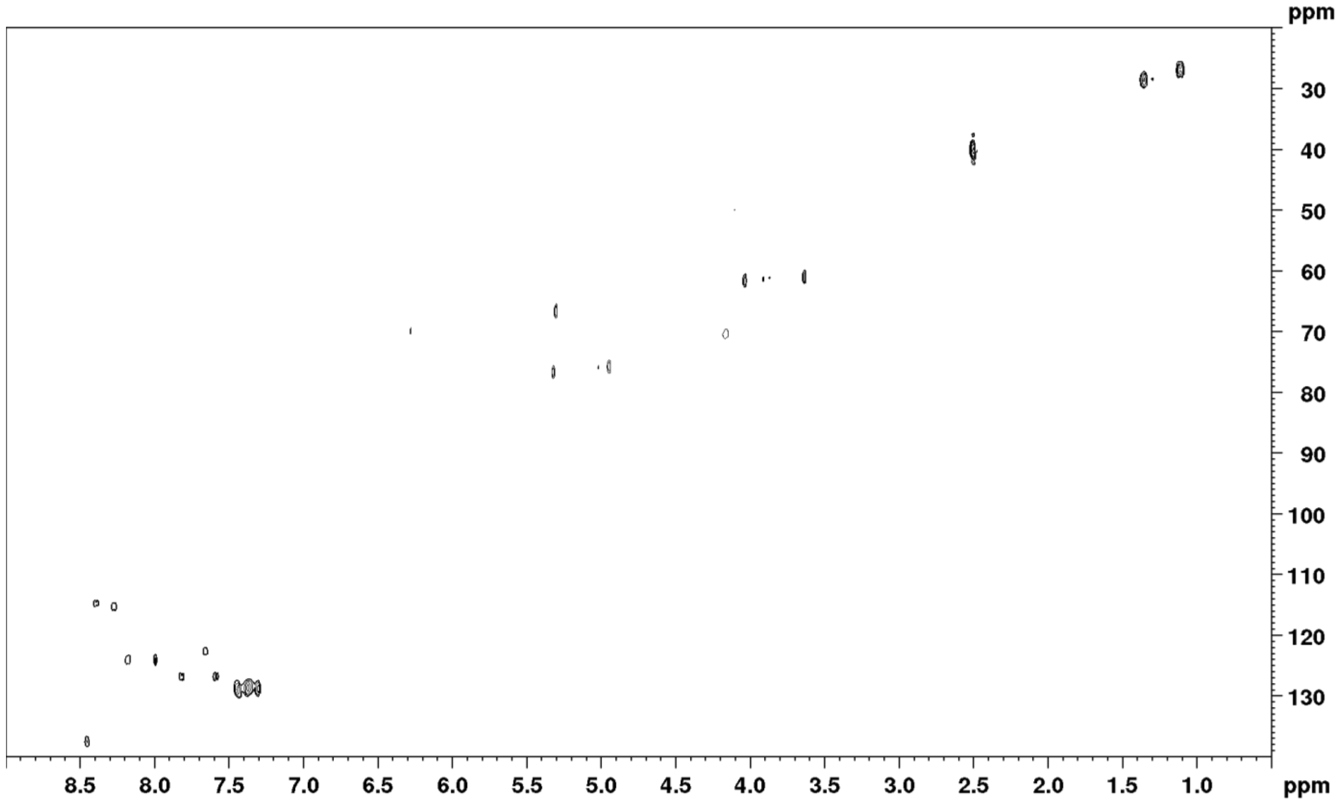

Boc-AzaHis(POM)-D(N)-E-F (17l)

^{1}H-NMR

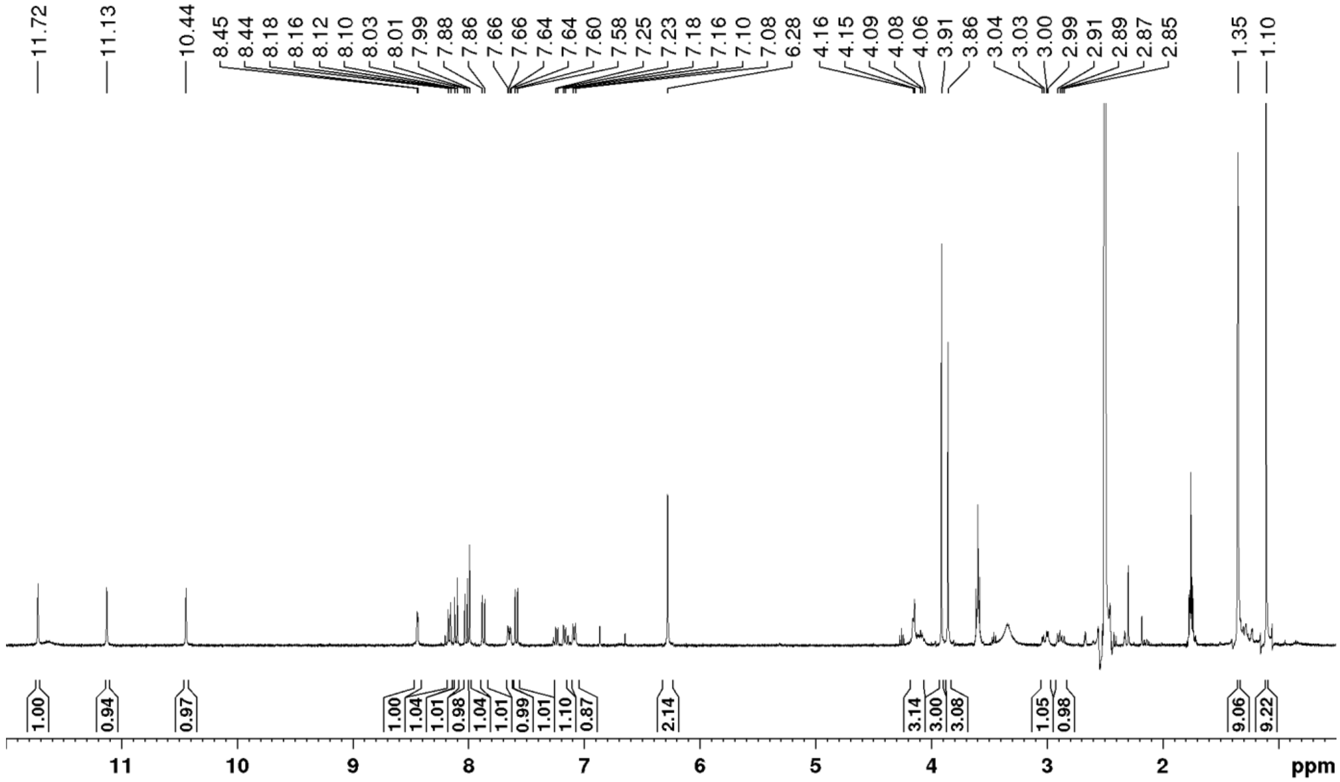

(^{1}H,^{13}C)-HSQC ed NMR

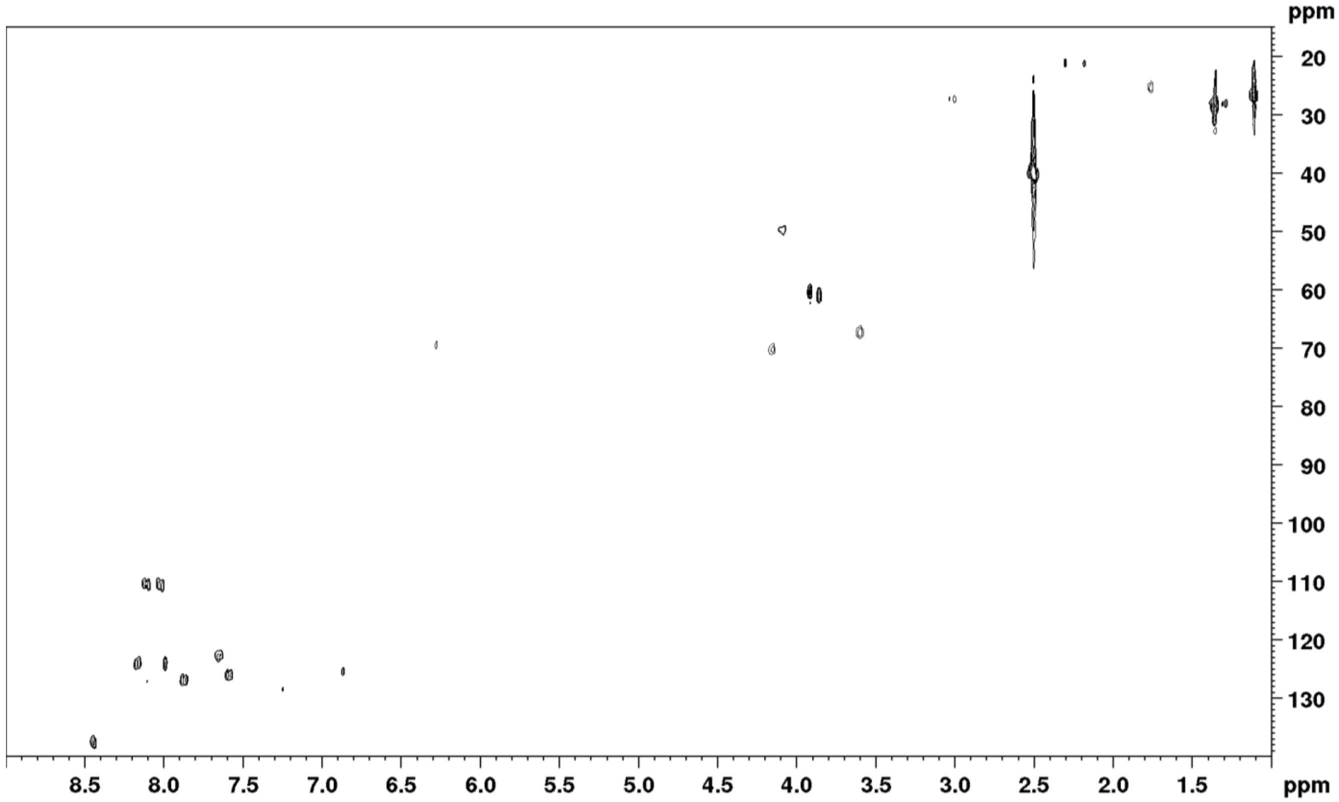

AzaHis-C(POM)-D(N)-E-F (17m)

^{1}H-NMR

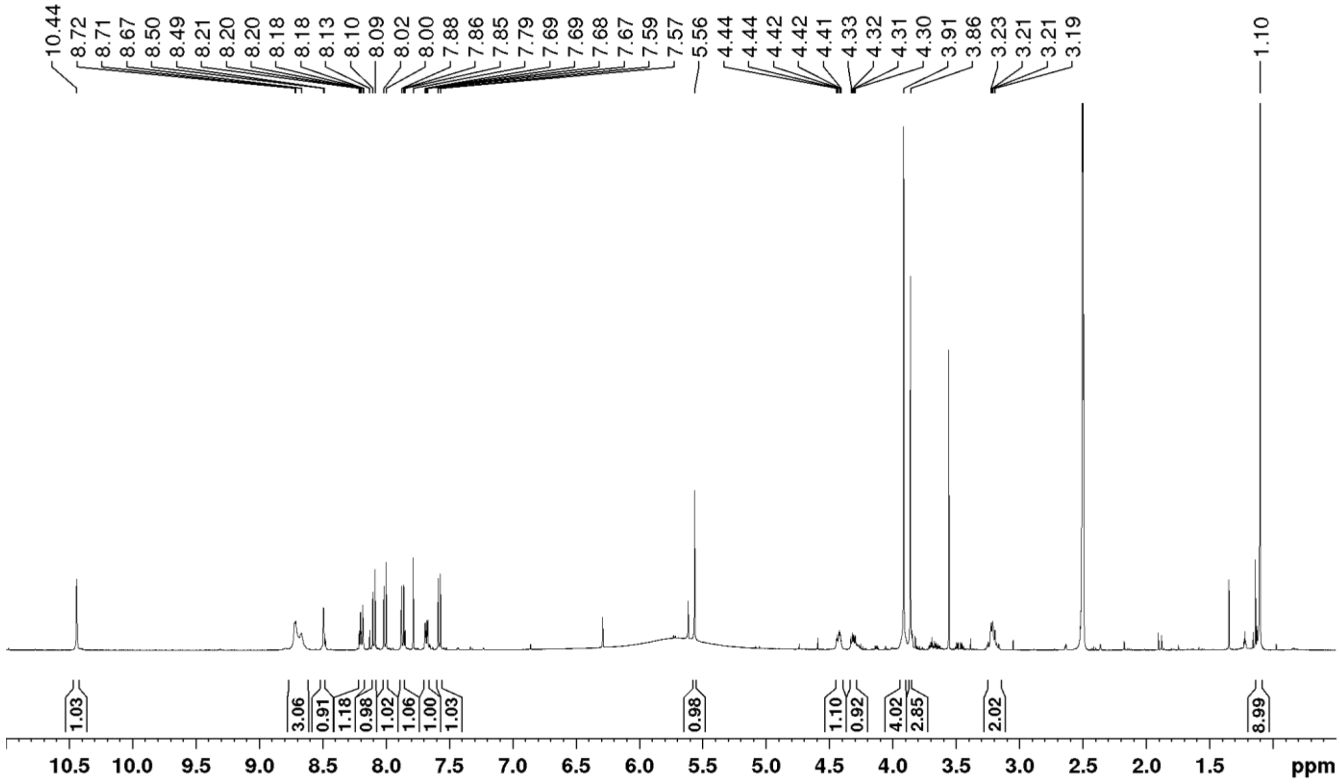

^{13}C-DEPT NMR

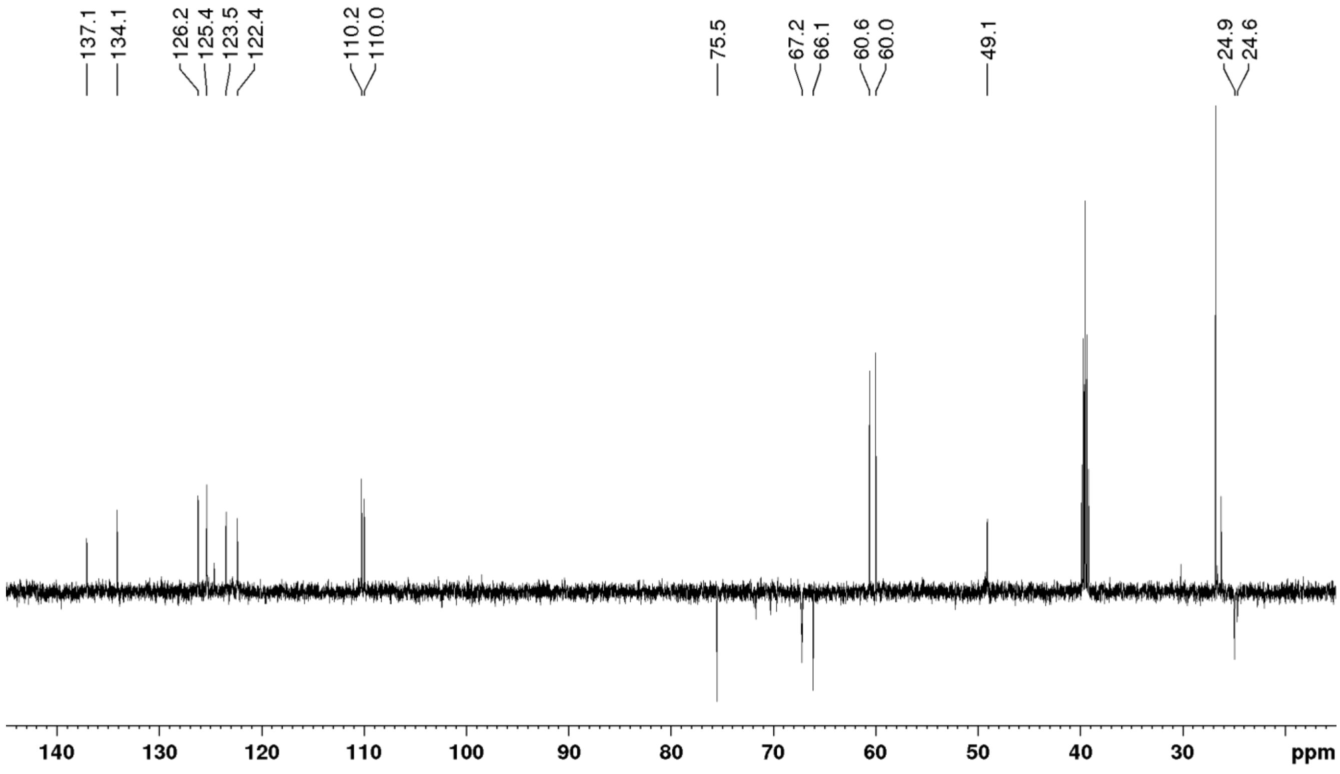

Ether-C-D-Isoster (17)

^{1}H-NMR

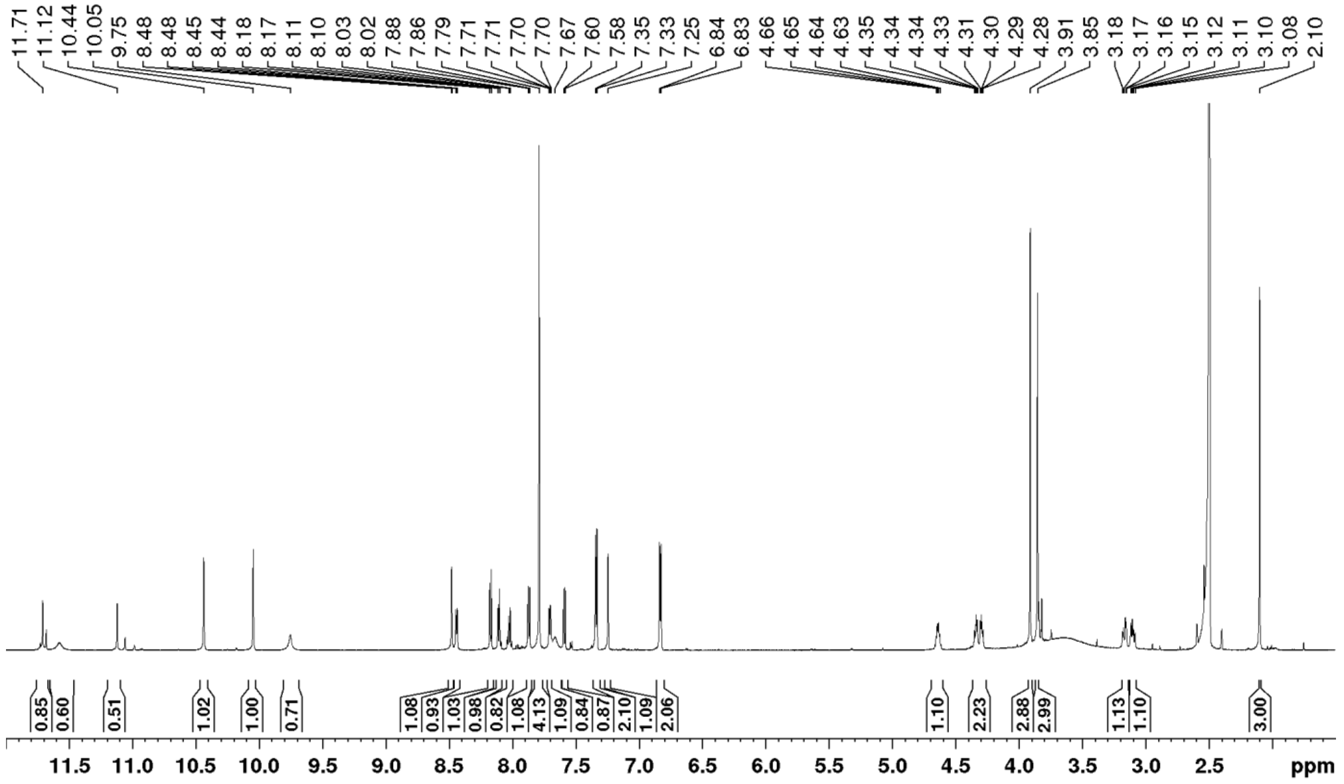

(^{1}H,^{13}C)-HSQC ed NMR

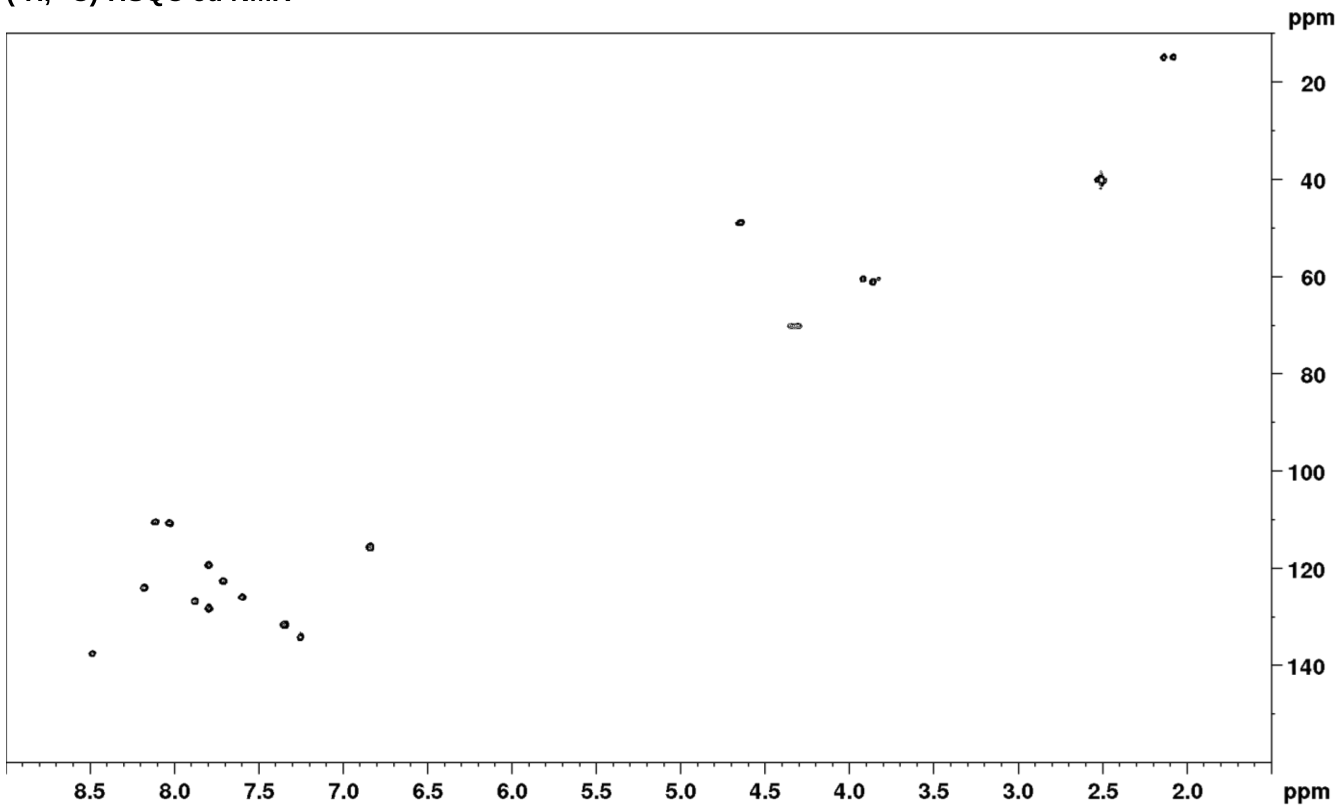

Cbz/Boc/Allyl-protected amino tetrapeptide precursor (20c)
^{1}H-NMR

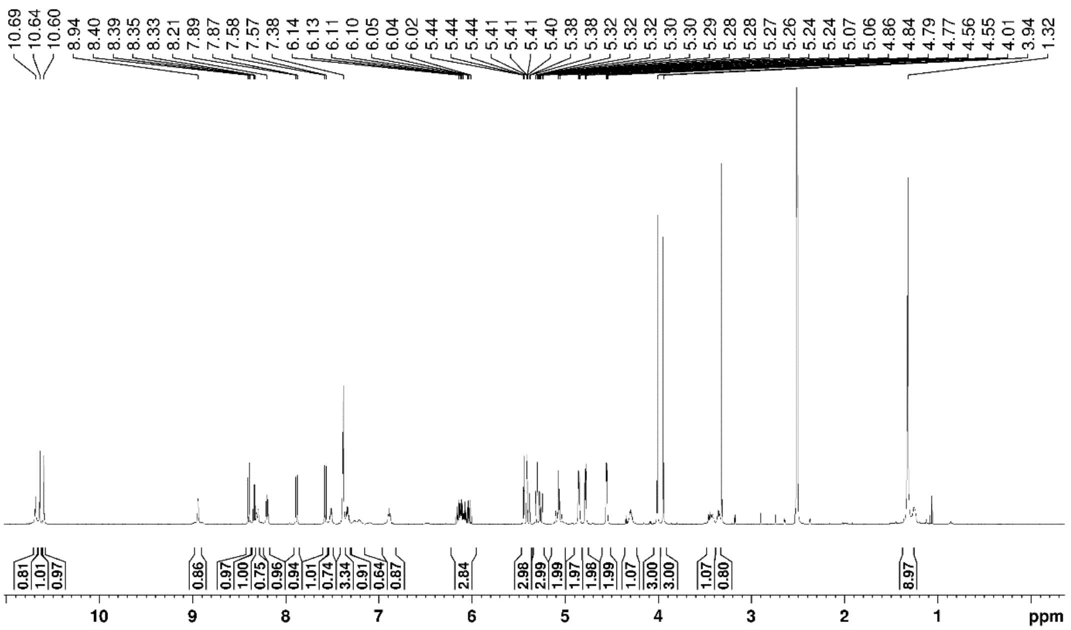

^{13}C-NMR

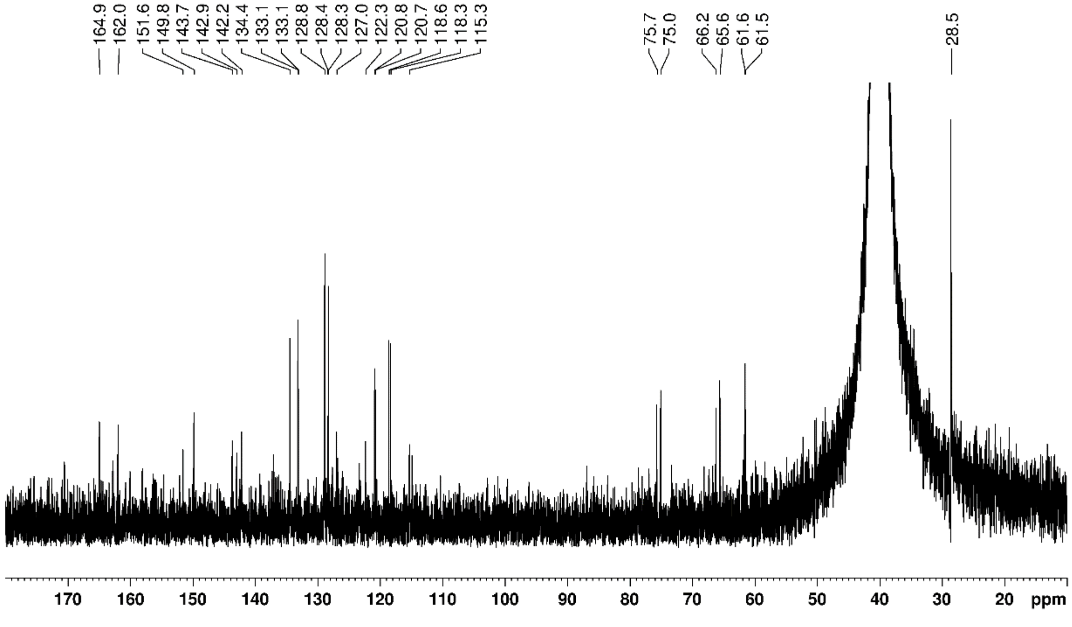

Cbz/Allyl-protected amino tetrapeptide precursor (20d)
^{1}H-NMR

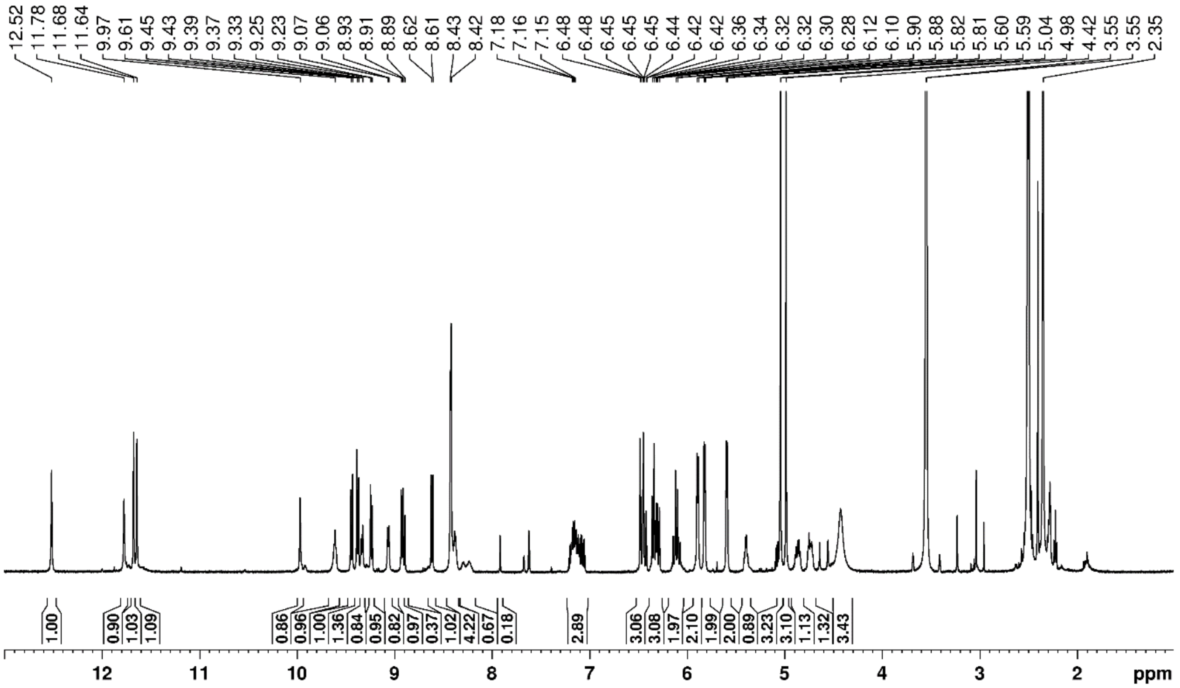

^{13}C-NMR

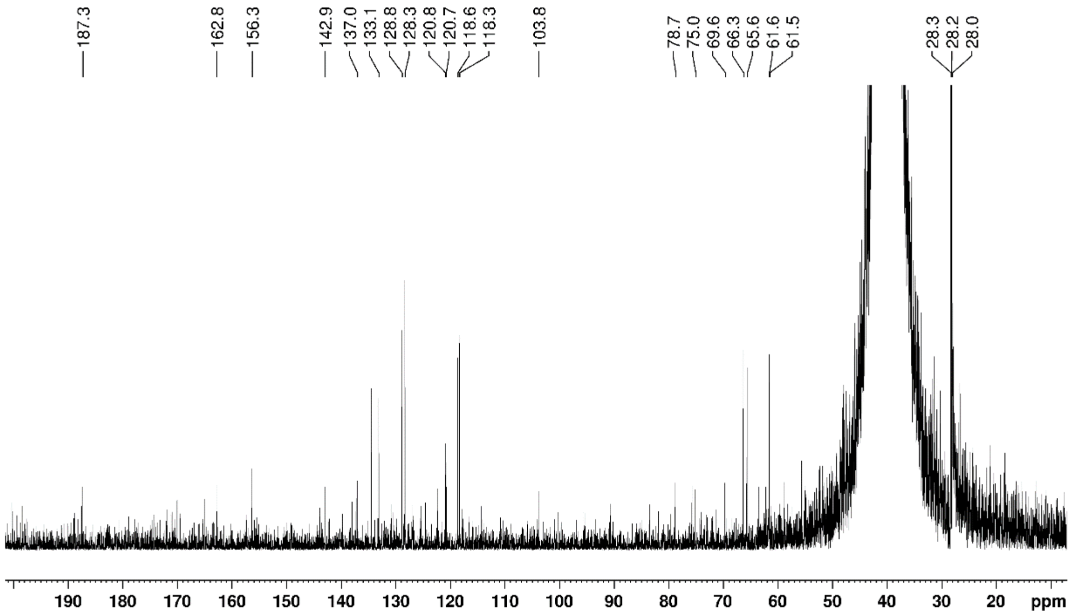

Cbz/Boc-protected guanidino tetrapeptide (20f)
^{1}H-NMR

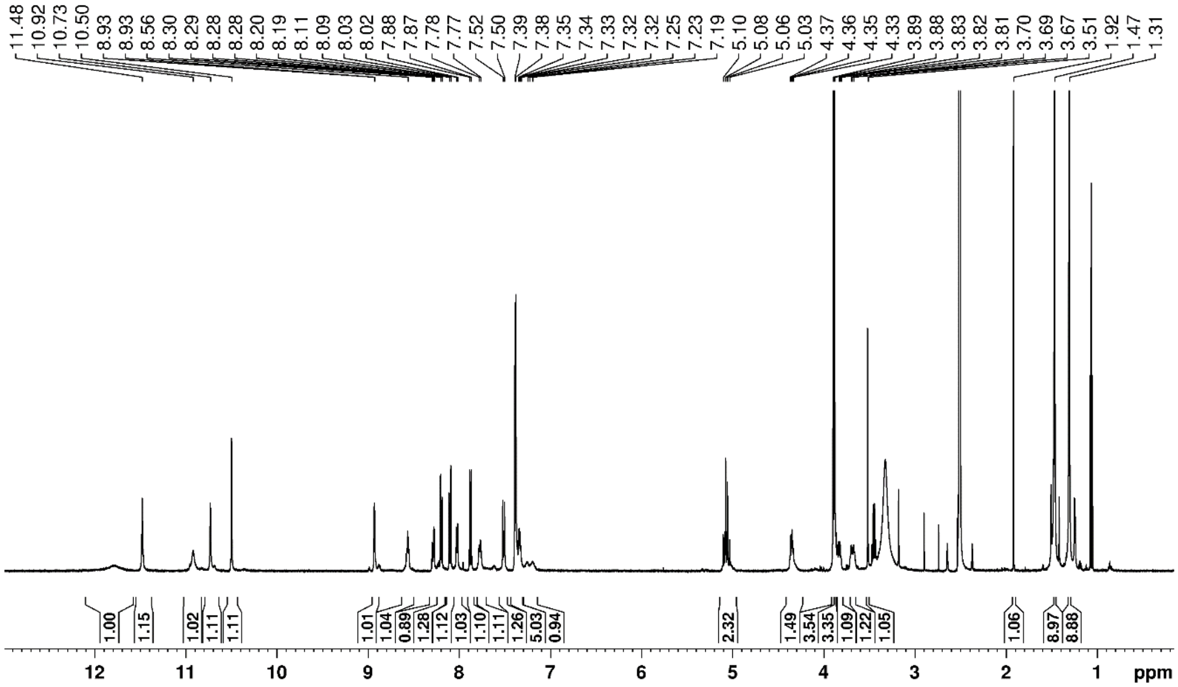

Guanidino-Albicidin (20)
^{1}H-NMR

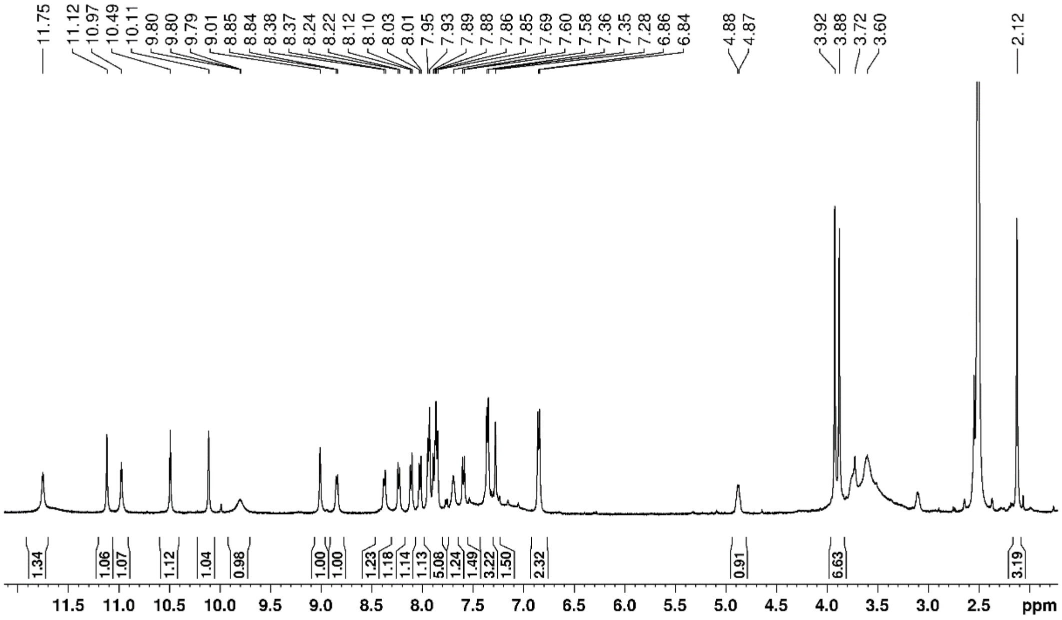

(^{1}H,^{13}C)-HSQC ed NMR

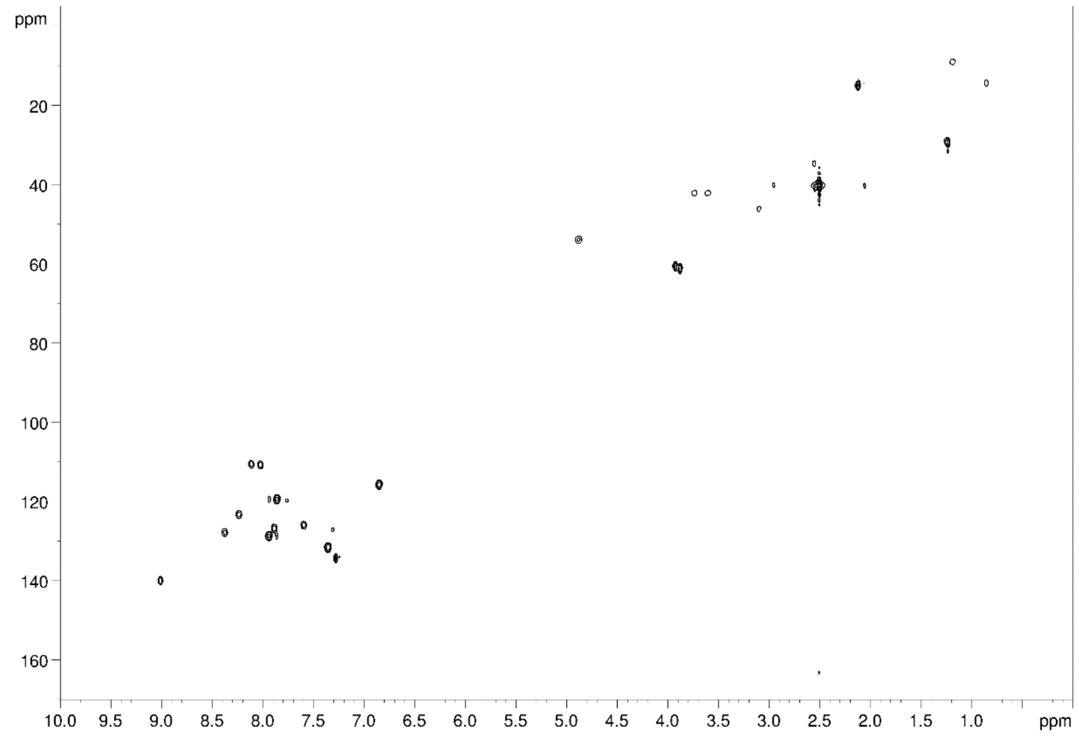

1-(*tert*-Butyl)-2-methyl (2*S*,4*R*)-4-hydroxypyrrolidin-1,2-dicarboxylat (22a)

^{1}H-NMR

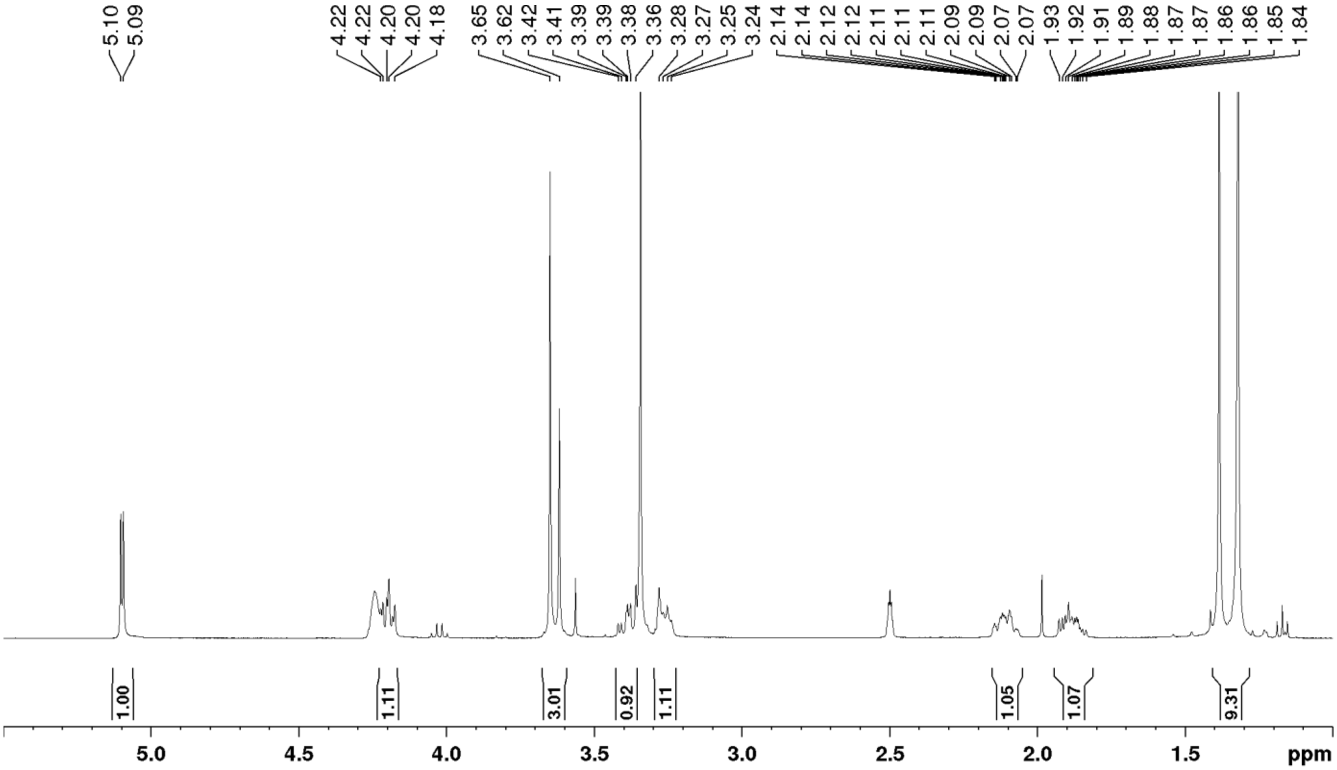

^{13}C-NMR

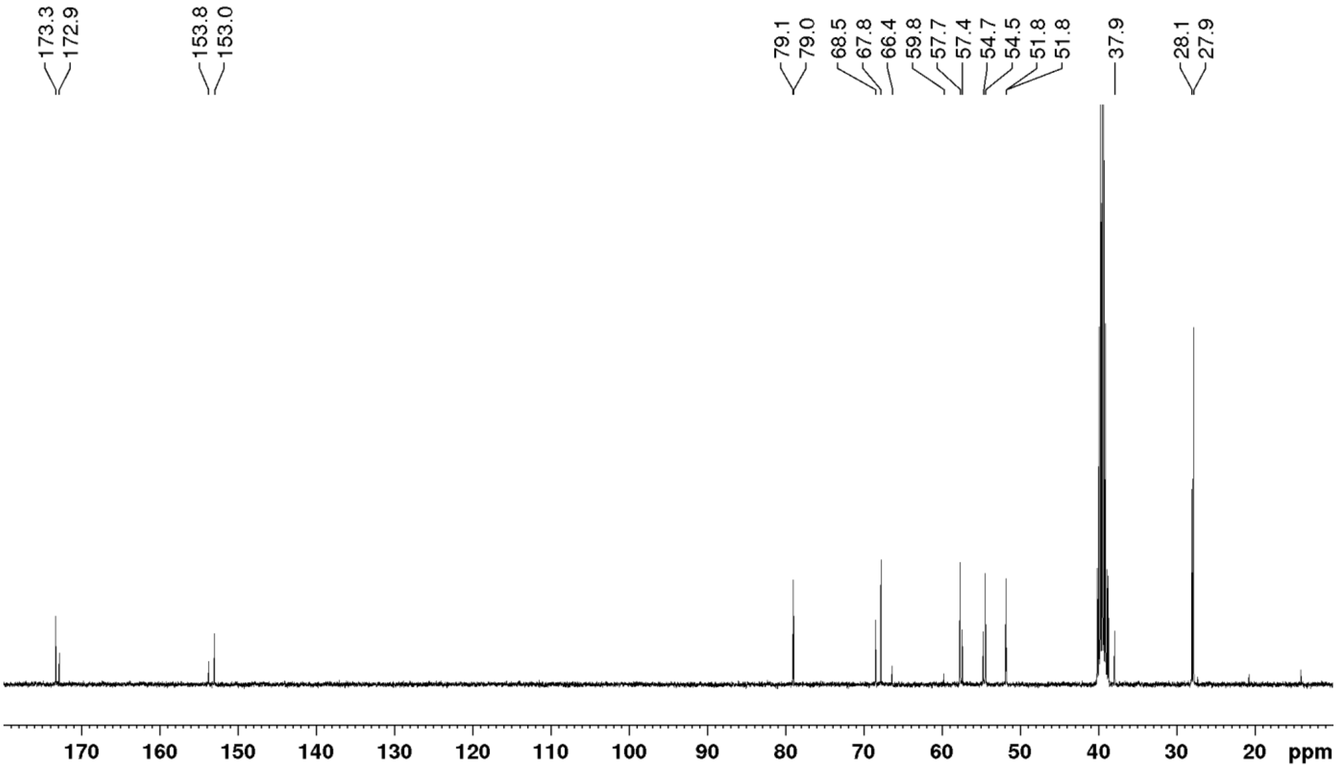

(2*S*,4*R*)-1-(*tert*-Butoxycarbonyl)-4-hydroxypyrrolidine-2-carbonic acid (22b)

^{1}H-NMR

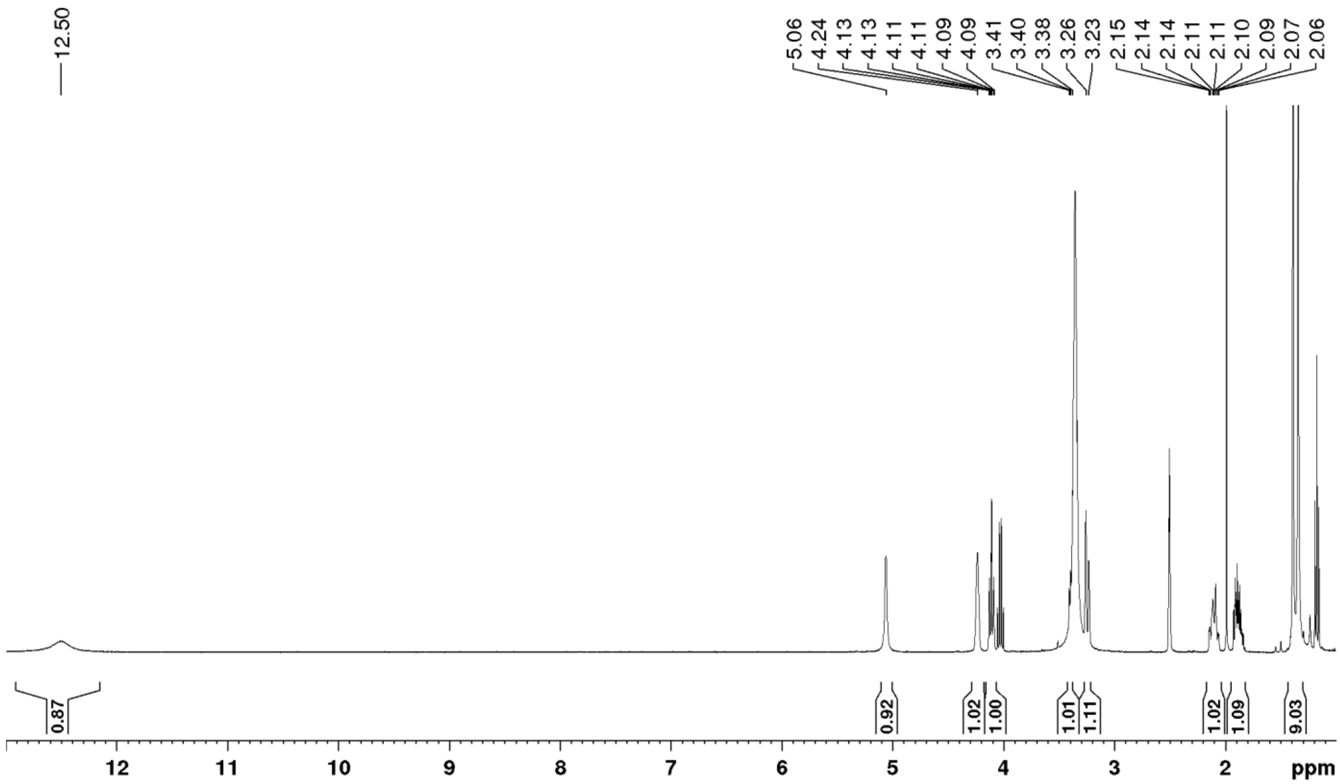

12.50
5.06
4.24
4.13
4.13
4.11
4.11
4.09
4.09
3.41
3.40
3.38
3.26
3.23
2.15
2.14
2.14
2.11
2.11
2.10
2.09
2.07
2.06
0.87
0.92
1.02
1.00
1.01
1.11
1.02
1.09
9.03
12
11
10
9
8
7
6
5
4
3
2
ppm

^{13}C-NMR

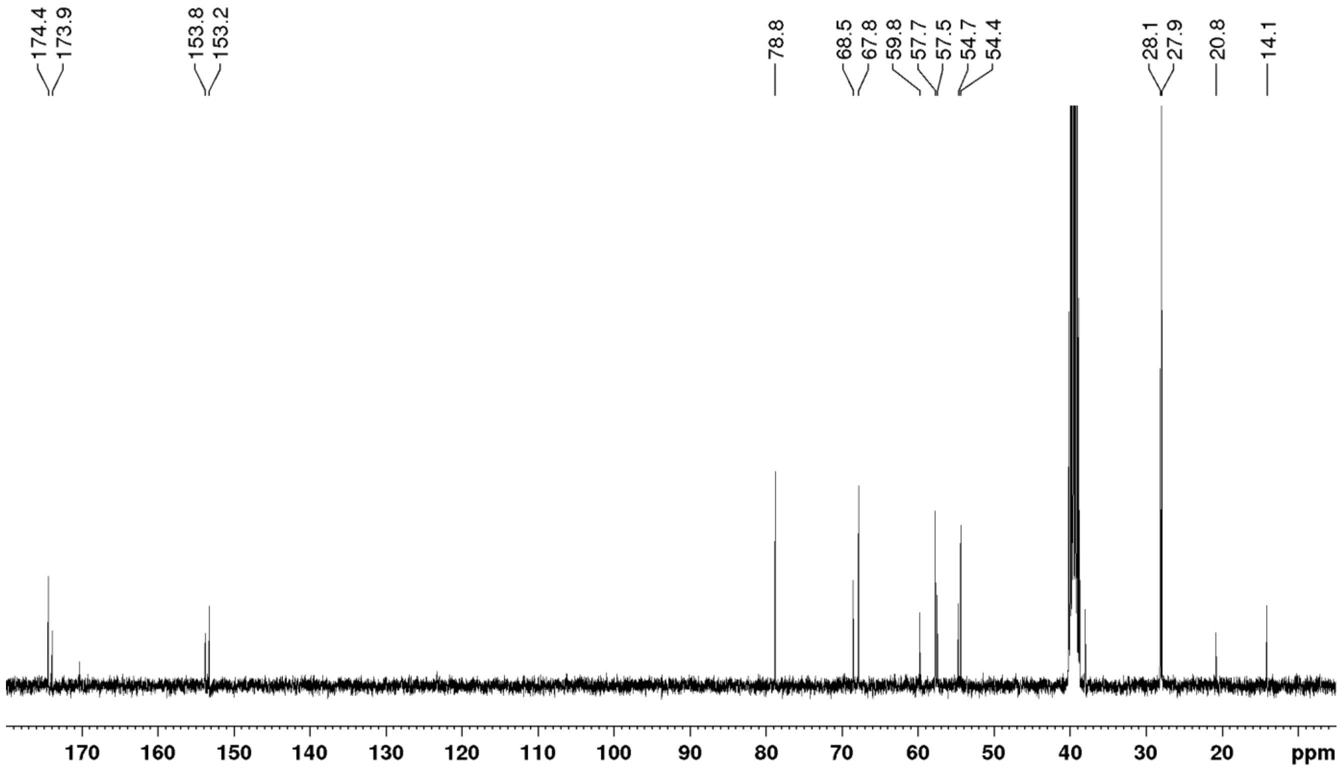

174.4
173.9
153.8
153.2
78.8
68.5
67.8
59.8
57.7
57.5
54.7
54.4
28.1
27.9
20.8
14.1
170
160
150
140
130
120
110
100
90
80
70
60
50
40
30
20
ppm

Boc-Hyp-pABA-HMpABA(Allyl)-HMpABA(Allyl)-OAllyl (22c)
^{1}H-NMR

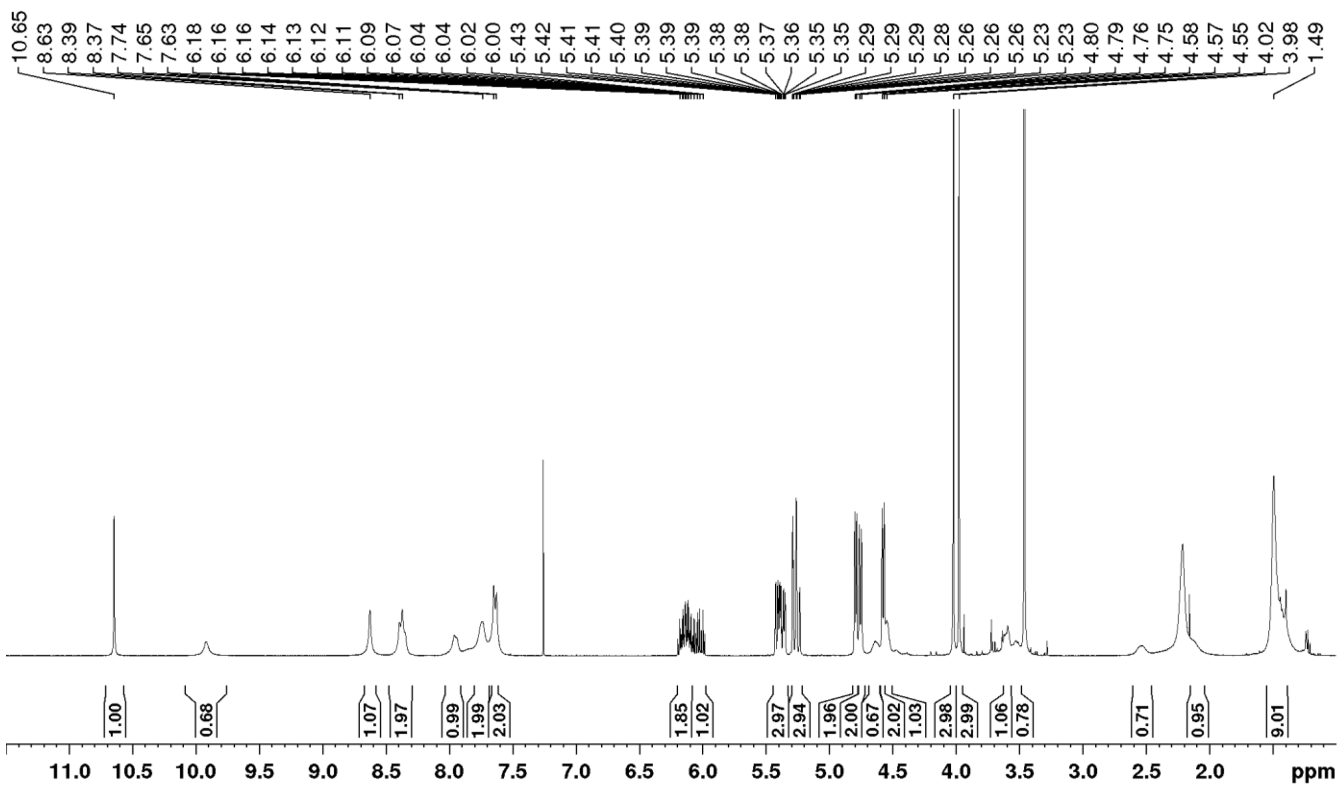

^{13}C-NMR

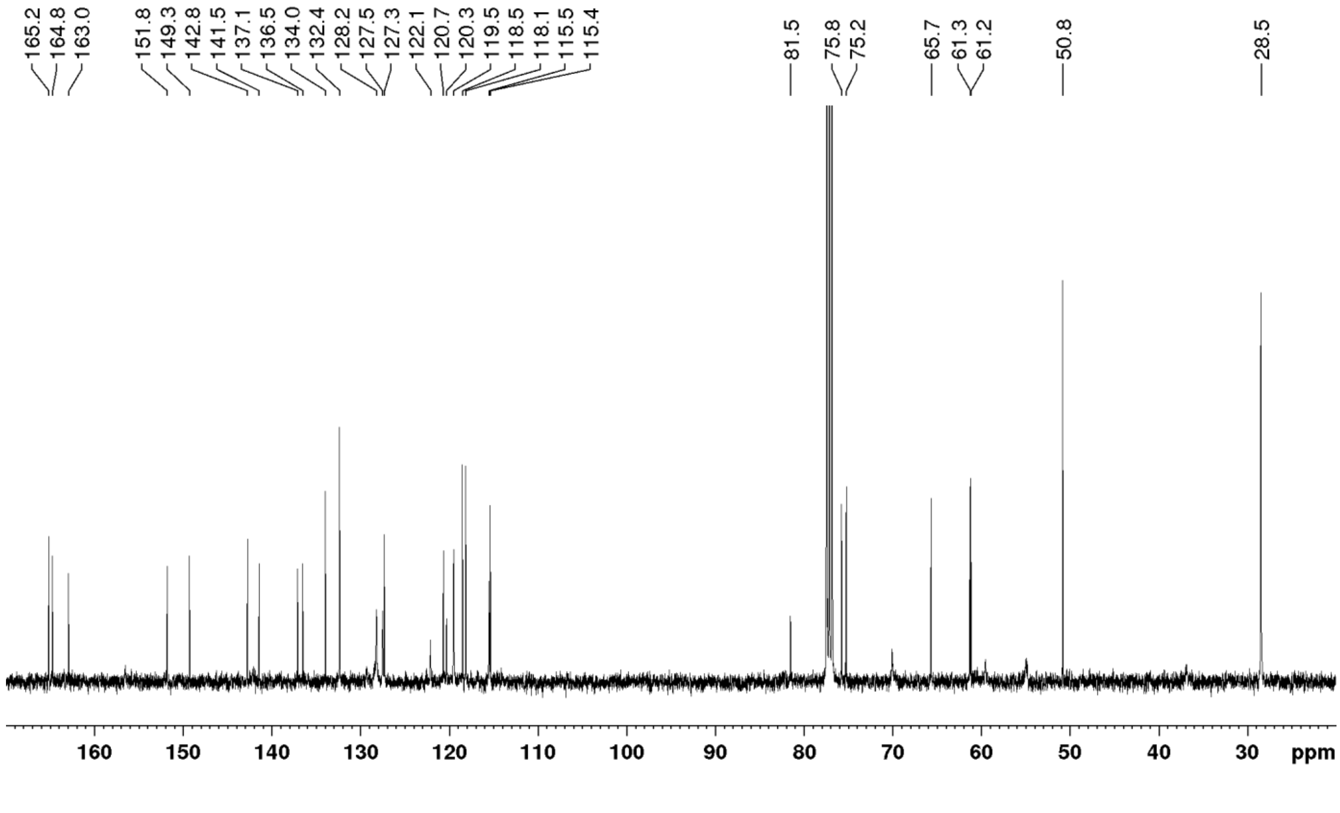

Boc-Hyp-pABA-HMpABA-HMpABA-OH (22d)
^{1}H-NMR

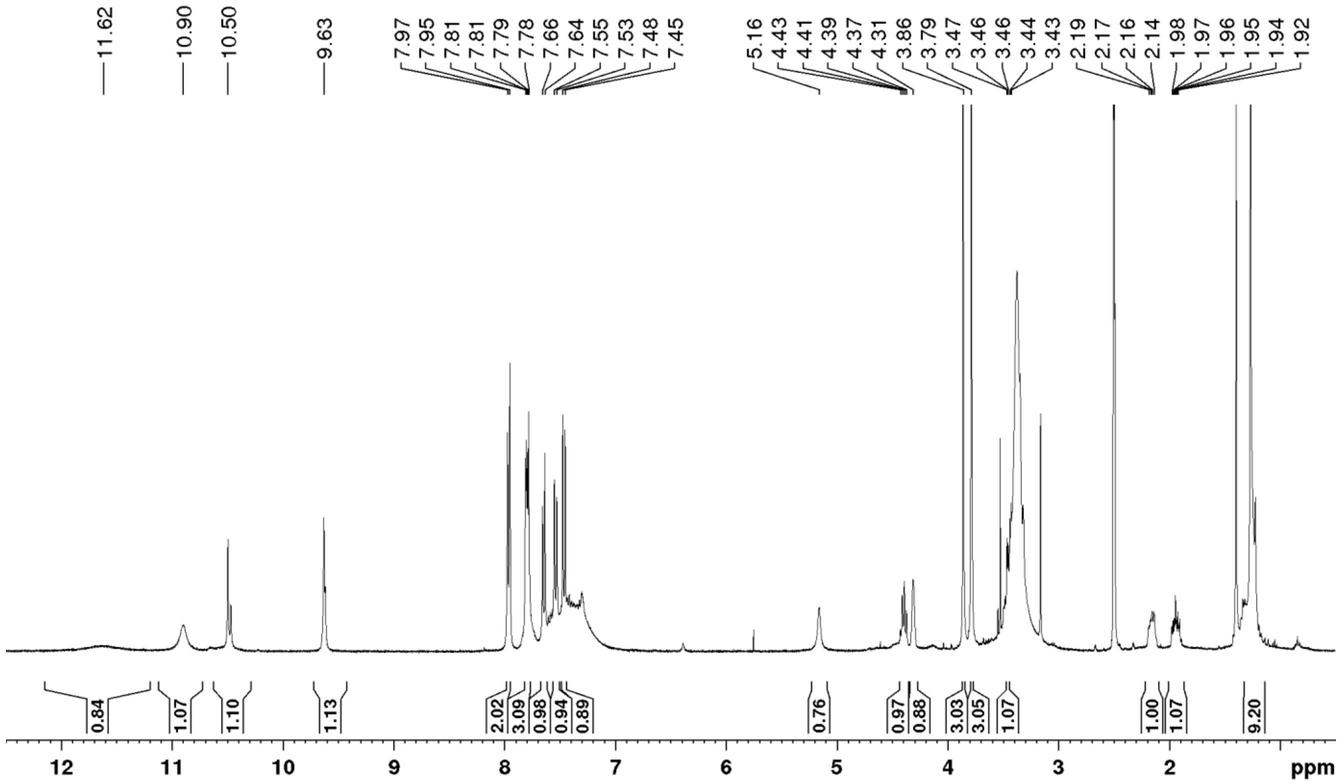

L-Hyp-Albicidin (22)
^{1}H-NMR

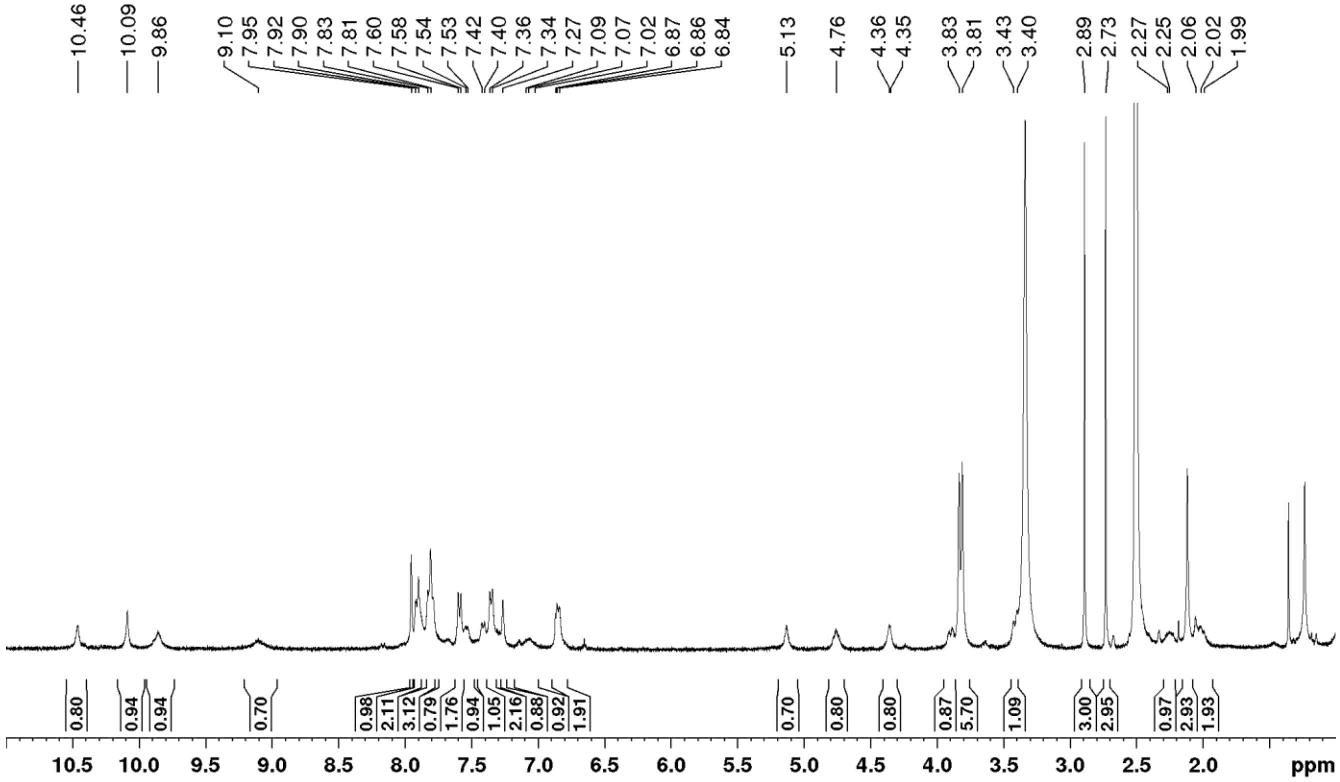

5. References

1 I. Behroz, P. Durkin, S. Grätz, M. Seidel, L. Rostock, M. Spinczyk, J. B. Weston and R. D. Süssmuth, *Chem. – A Eur. J.*, 2019, **25**, 16538–16543.

2 J. Shong, Y.-M. Huang, C. Bystroff and C. H. Collins, *ACS Chem. Biol.*, 2013, **8**, 789–795.

3 D. G. Gibson, L. Young, R.-Y. Chuang, J. C. Venter, C. A. Hutchison and H. O. Smith, *Nat. Methods*, 2009, **6**, 343–345.

4 C. Gregor, K. C. Gwosch, S. J. Sahl and S. W. Hell, *Proc. Natl. Acad. Sci.*, 2018, **115**, 962–967.

5 J. Chiu, *Nucleic Acids Res.*, 2004, **32**, e174–e174.

6 L. Rostock, R. Driller, S. Grätz, D. Kerwat, L. von Eckardstein, D. Petras, M. Kunert, C. Alings, F.-J. Schmitt, T. Friedrich, M. C. Wahl, B. Loll, A. Mainz and R. D. Süssmuth, *Nat. Commun.*, 2018, **9**, 3095.

7 E. Gasteiger, C. Hoogland, A. Gattiker, S. Duvaud, M. R. Wilkins, R. D. Appel and A. Bairoch, in *The Proteomics Protocols Handbook*, Humana Press, Totowa, NJ, 2005, pp. 571–607.

8 E. F. Pettersen, T. D. Goddard, C. C. Huang, G. S. Couch, D. M. Greenblatt, E. C. Meng and T. E. Ferrin, *J. Comput. Chem.*, 2004, **25**, 1605–1612.

9 O. Trott and A. J. Olson, *J. Comput. Chem.*, 2009, **31**, 455–461.

10 S. Kim, E. E. Bolton and S. H. Bryant, *J. Cheminform.*, 2013, **5**, 1.

11 M. Varadi, S. Anyango, M. Deshpande, S. Nair, C. Natassia, G. Yordanova, D. Yuan, O. Stroe, G. Wood, A. Laydon, A. Žídek, T. Green, K. Tunyasuvunakool, S. Petersen, J. Jumper, E. Clancy, R. Green, A. Vora, M. Lutfi, M. Figurnov, A. Cowie, N. Hobbs, P. Kohli, G. Kleywegt, E. Birney, D. Hassabis and S. Velankar, *Nucleic Acids Res.*, 2022, **50**, D439–D444.

12 J. Jumper, R. Evans, A. Pritzel, T. Green, M. Figurnov, O. Ronneberger, K. Tunyasuvunakool, R. Bates, A. Žídek, A. Potapenko, A. Bridgland, C. Meyer, S. A. A. Kohl, A. J. Ballard, A. Cowie, B. Romera-Paredes, S. Nikolov, R. Jain, J. Adler, T. Back, S. Petersen, D. Reiman, E. Clancy, M. Zielinski, M. Steinegger, M. Pacholska, T. Berghammer, S. Bodenstein, D. Silver, O. Vinyals, A. W. Senior, K. Kavukcuoglu, P. Kohli and D. Hassabis, *Nature*, 2021, **596**, 583–589.

13 M. Mirdita, K. Schütze, Y. Moriwaki, L. Heo, S. Ovchinnikov and M. Steinegger, *Nat. Methods*, 2022, **19**, 679–682.

14 J. Kretz, D. Kerwat, V. Schubert, S. Grätz, A. Pesic, S. Semsary, S. Cociancich, M. Royer and R. D. Süssmuth, *Angew. Chemie Int. Ed.*, 2015, **54**, 1969–1973.

15 S. Grätz, D. Kerwat, J. Kretz, L. von Eckardstein, S. Semsary, M. Seidel, M. Kunert, J. B. Weston and R. D. Süssmuth, *ChemMedChem*, 2016, **11**, 1499–1502.

16 L. Vieweg, J. Kretz, A. Pesic, D. Kerwat, S. Grätz, M. Royer, S. Cociancich, A. Mainz and R. D. Süssmuth, *J. Am. Chem. Soc.*, 2015, **137**, 7608–7611.

17 E. Michalczyk, K. Hommernick, I. Behroz, M. Kulike, Z. Pakosz-Stępień, L. Mazurek, M. Seidel, M. Kunert, K. Santos, H. von Moeller, B. Loll, J. B. Weston, A. Mainz, J. G. Heddle, R. D. Süssmuth and D. Ghilarov, *Nat. Catal.*, 2023, **6**, 52–67.

18 L. Zborovsky, L. Kleebauer, M. Seidel, A. Kostenko, L. von Eckardstein, F. O. Gombert, J. Weston and R. D. Süssmuth, *Chem. Sci.*, 2021, **12**, 14606–14617.

19 H. Lin, D. S. Yamashita, J. Zeng, R. Xie, W. Wang, S. Nidarmarthy, J. I. Luengo, N. Rhodes, V. B. Knick, A. E. Choudhry, Z. Lai, E. A. Minthorn, S. L. Strum, E. R. Wood, P. A. Elkins, N. O. Concha and D. A. Heerding, *Bioorg. Med. Chem. Lett.*, 2010, **20**, 673–678.

20 J. Weweler, S. L. Younas and J. Streuff, *Angew. Chemie Int. Ed.*, 2019, **58**, 17700–17703.

21 L. von Eckardstein, D. Petras, T. Dang, S. Cociancich, S. Sabri, S. Grätz, D. Kerwat, M. Seidel, A. Pesic, P. C. Dorrestein, M. Royer, J. B. Weston and R. D. Süssmuth, *Chem. - A Eur. J.*, 2017, **23**, 15316–15321.

22 L.-X. Weng, J.-L. Xu, Q. Li, R. G. Birch and L.-H. Zhang, *Microbiology*, 2003, **149**, 451–457.

23 L.-X. Weng, L.-H. Wang, J.-L. Xu, J.-E. Wu, Q. Li and L.-H. Zhang, *Appl. Environ. Microbiol.*, 2005, **71**, 1445–1452.

24 A. Sikandar, K. Cirnski, G. Testolin, C. Volz, M. Brönstrup, O. V. Kalinina, R. Müller and J. Koehnke, *J. Am. Chem. Soc.*, 2018, **140**, 16641–16649.
